# Inactive **β**_1_-integrin acts as a junctional scaffold for angiopoietin/TIE2/FOXO1 signaling

**DOI:** 10.1172/JCI190552

**Published:** 2026-06-15

**Authors:** Tuomas Sipilä, Srinivas Kumar Ponna, Abhinandan Venkatesha Murthy, Anne Pink, Giray Enkavi, Shraman Kumar Bohra, Klaudia Lewna, Keerthana Ganesh, Qina Liu, Mirka Korhonen, Tommi Kajander, Michael Potente, Johanna Ivaska, Ilpo Vattulainen, Veli-Matti Leppänen, Pipsa Saharinen

**Affiliations:** 1 Translational Cancer Medicine Program, Research Programs Unit,; 2 Department of Physics, and; 3 Institute of Biotechnology, University of Helsinki, Helsinki, Finland.; 4 Angiogenesis & Metabolism Laboratory, Center of Vascular Biomedicine, Berlin Institute of Health at Charité – Universitätsmedizin Berlin, Berlin, Germany & Max Delbrück Center for Molecular Medicine in the Helmholtz Association, Berlin, Germany.; 5 Turku Bioscience Centre, University of Turku and Åbo Akademi University, Turku, Finland.; 6 InFLAMES Research Flagship and Department of Life Technologies, University of Turku, Turku, Finland.; 7 Wihuri Research Institute, Biomedicum Helsinki, Helsinki, Finland.; 8 Department of Biochemistry and Developmental Biology, Faculty of Medicine, University of Helsinki, Finland.

**Keywords:** Angiogenesis, Cell biology, Vascular biology, Growth factors, Integrins

## Abstract

The blood and lymphatic vascular systems are regulated by angiopoietin (ANGPT) growth factors, which signal via endothelial TIE receptor tyrosine kinases and integrins. However, mechanistic understanding of how these receptors crosstalk is limited. Here, we show how β_1_-integrin inactivation regulates endothelial ANGPT/TIE2 signaling. By integrating biophysical analyses, X-ray crystallography, size-exclusion chromatography–small-angle X-ray scattering and atomistic molecular dynamics simulations, we show that ANGPT2 binds through its asymmetrically positioned C-terminal fibrinogen-like domains to both TIE2 and α_5_β_1_-integrin, forming a trimeric complex compatible with the inactive α_5_β_1_-integrin conformation. Inactive β_1_-integrin colocalizes with ANGPT-induced TIE2 in cell-cell junctions and stabilizing β_1_-integrin in its inactive state enhances junctional TIE2 accumulation and promotes nuclear exclusion of the TIE2 transcriptional effector FOXO1 in cultured endothelial cells. Endothelial-specific β_1_-integrin deletion in adult mice reduces venous TIE2 phosphorylation, whereas endotoxemia diminishes junctional β_1_-integrin along with decreased phosphorylated TIE2. In contrast, without TIE2, ANGPT2 uniquely engages active β_1_-integrin, via its N-terminal superclustering domain. Altogether, our results provide structural and mechanistic evidence of ANGPT signaling via α_5_β_1_-integrin and support a model in which inactive α_5_β_1_-integrin acts as a junctional scaffold for ANGPT/TIE2/FOXO1 signaling, explaining how integrin conformational switching spatially organizes growth factor signaling in the endothelium.

## Abstract 2

X-ray crystallography and SEC-SAXS reveal novel structures of angiopoietin-2 in complex with the TIE2 receptor tyrosine kinase and a5b1-integrin explaining receptor crosstalk in endothelium

## Graphical abstract

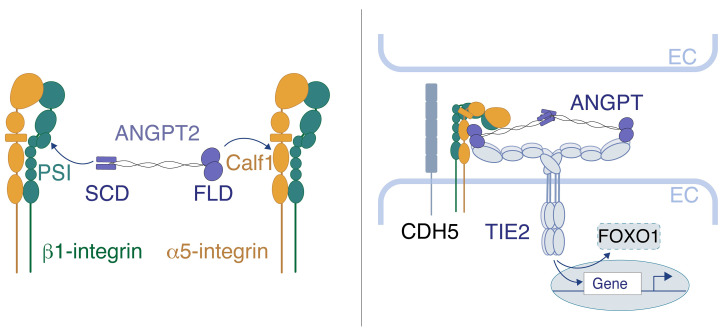

## Introduction

Vascular development and homeostasis are controlled by signaling through endothelial growth factor receptors and integrins (1, 2). These receptor systems co-operate to regulate endothelial cell (EC) functions (2–5); however, the molecular mechanisms of integrin–growth factor receptor crosstalk remain poorly understood (6).

ANGPT growth factors (ANGPT1 and ANGPT2) regulate both lymphatic and blood vascular development (7). Inactivating gene variants in the ANGPT receptor *TEK* (encoding the TIE2 RTK) and in *ANGPT1* are linked to defective Schlemm’s canal formation leading to glaucoma (8, 9), whereas activating *TEK* mutations cause venous malformations (10). Loss-of-function mutations in *ANGPT2* underlie primary human lymphedema, and *Angpt2*-deficient mice display defective lymphangiogenesis and impaired lymphatic EC (LEC) junctions (8, 11–13). Beyond development, the ANGPT/TIE pathway maintains vascular stability, whereas inflammation-induced endothelial ANGPT2 upregulation and reduced TIE signaling drive vascular leak and edema, such as in sepsis (14, 15). These mechanisms provide the rationale for ANGPT2-targeted therapies in edematous diseases, such as diabetic macular edema (7).

ANGPTs primarily signal through their C-terminal fibrinogen-like domain (FLD) by engaging the TIE2 RTK (16), which forms a complex with TIE1 (14) and is recruited to EC-EC junctions in *trans* (17, 18). ANGPT1 functions as an obligatory TIE2 agonist, activating the PI3K/AKT pathway and leading to inactivation of the Forkhead box protein O1 (FOXO1) transcription factor, among other downstream effectors. ANGPT2, by contrast, is a weaker agonist that can limit the vascular stabilizing ANGPT1/TIE2 pathway in a context-dependent manner (14, 15). In LECs, ANGPT2 is secreted in an autocrine manner and acts as a TIE2 agonist, highlighting the context-dependent function of ANGPT2 signaling across vascular beds (11, 19, 20). Evidence further suggests that ANGPTs signal via integrins in different pathophysiological processes, including angiogenesis, vascular destabilization, neuroinflammation, ischemic heart injury, adipocyte regulation, and insulin resistance (21–25). However, the mechanisms by which ANGPT signaling is coordinated through integrins are not well understood.

The activity of integrin heterodimers, composed of α and β subunits, is regulated via a conformational switch, whereby integrins alternate between an inactive closed, bent conformation and an active, extended, open conformation (26). This conformational activation correlates with increased binding of intracellular activators and extracellular matrix (ECM) proteins (27). Additionally, clustering of active integrins concentrates multiple binding affinities into defined locations, stabilizing different types of integrin adhesions such as focal complexes, focal adhesions, and fibrillar adhesions engaging in signaling and mechanotransduction (27). However, already more than 3 decades ago, it was noted that integrins in tissue localize to cell-cell junctions, indicative of previously unrecognized functions for integrins in cell-cell interactions (28, 29). Subsequently, a few studies have reported integrins in cell junctions (30–32). However, their junctional role has remained obscure.

Here, we reveal how integrin conformational switching regulates the ANGPT/TIE2/FOXO1 signaling in ECs. Using protein interaction analysis, crystallography, small-angle X-ray scattering (SAXS), and atomistic molecular dynamics (MD) simulations, we show that ANGPT2 simultaneously interacts through its asymmetric receptor-binding domains with both TIE2 and α_5_β_1_-integrin. Unexpectedly, TIE2 colocalizes with inactive β_1_-integrin in EC-EC junctions, where it drives FOXO1 inactivation, prompting us to model a trimeric complex, in which α_5_β_1_-integrin adopts an inactive conformation. In contrast, in the absence of TIE2, ANGPT2 engages active β_1_-integrins through the β_1_-integrin plexin-semaphorin-integrin (PSI) domain and the ANGPT2 superclustering domain (SCD). These results define a mode of receptor crosstalk regulated by active and inactive integrin conformations, whereby the inactive α_5_β_1_-integrin, long considered as signaling incompetent, serves as a cell junction–localized molecular scaffold coupling ANGPT2/TIE2 and FOXO1 signaling in ECs.

## Results

### ANGPT2 and TIE2 differentially interact with active and inactive β_1_-integrin at endothelial junctions.

**Figure 1:**
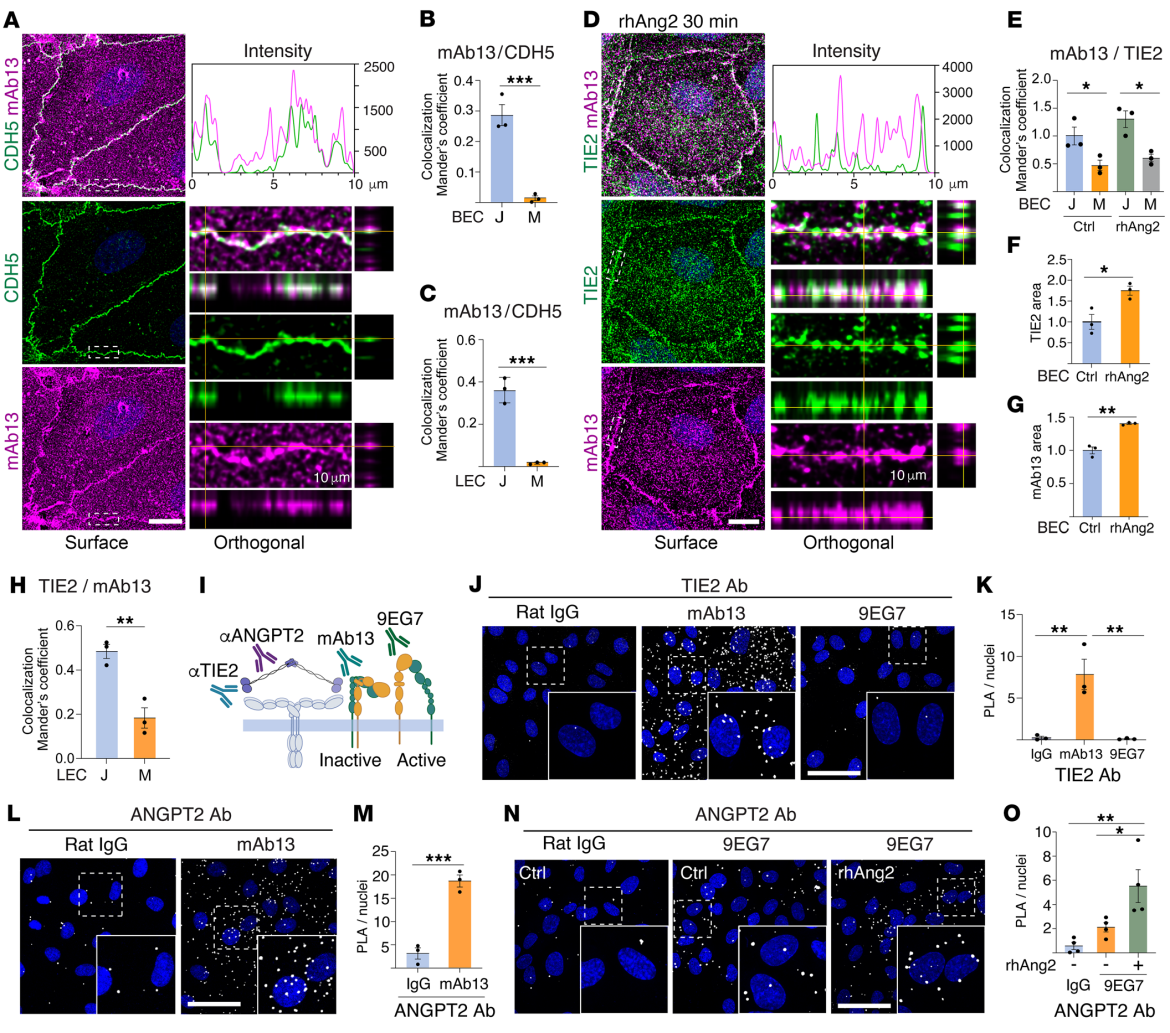
ANGPT2 and TIE2 differentially interact with active and inactive β_1_-integrin at endothelial junctions. (**A**) Surface staining of cultured BECs for inactive β_1_-integrin (mAb13) and VE-cadherin (CDH5). (**B** and **C**) Mander’s overlap coefficient (MOC) for mAb13/CDH5 colocalization in junctions (J) versus membrane (M) in BECs (**B**) and LEC (**C**) (image shown in Supplemental Figure 1B). (**D**) Surface staining of rhAng2-stimulated and control (Ctrl) (image shown in Supplemental Figure 1G) BECs for mAb13 and TIE2. (**E**) MOC for mAb13/TIE2 colocalization in junctions versus membrane in Ctrl and rhAng2-stimulated BECs, relative to Ctrl-treated BEC junctions. (**F** and **G**) Quantification of TIE2 (**F**) and mAb13 (**G**) in junctions in Ctrl and rhAng2-stimulated BECs. (**H**) MOC for TIE2/mAb13 colocalization in LECs. Image shown in Supplemental Figure 1F. (**I**) PLA design using antibodies for inactive β_1_-integrin (mAb13), active β_1_-integrin (9EG7), TIE2, and ANGPT2. Isotype-IgG as control. (**J**–**O**) PLA in HUVECs using indicated antibodies and quantification normalized to nuclei (**J** and **K**: TIE2/mAb13/9EG7; **L** and **M**: ANGPT2/mAb13; **N** and **O**: ANGPT2/9EG7 ± rhAng2 stimulation). Scale bars: 10 μm (**A** and **D**), 50 μm (**J**, **L**, and **N**). Confocal Z-stack projections are shown. *n* = 3 independent experiments (except in **O**, *n* = 4); 150 regions of interest per sample (**B**, **C**, and **E**–**H**). Statistical tests were a 2-tailed *t* test (**B**, **C**, **F**–**H**, and **M**), 1-way ANOVA with Tukey’s post hoc test (**K** and **O**), 2-way ANOVA with multiple correction (**E**). Data are presented as mean ± SEM. *P < 0.05, **P < 0.01, ***P < 0.001.

To investigate the spatial convergence of ANGPT2/TIE2 and integrin signaling pathways, we first analyzed the subcellular localization of β_1_-integrin, the predominant integrin subunit expressed in ECs. Whereas active ECM-bound β_1_-integrins are well characterized for their signaling via adhesion complexes on the basolateral cell surface, the localization of inactive β_1_-integrins in ECs remains less well understood (33). Using the conformation-specific antibody mAb13, which selectively recognizes the inactive, bent β_1_-integrin conformation (34), we detected inactive β_1_-integrin on the surface of both blood microvascular ECs (BECs) and dermal LECs, which were chosen as separate EC models. Interestingly, inactive β_1_-integrin localized to EC-EC junctions, where it colocalized with the adherens junction protein VE-cadherin (CDH5) (Figure 1, A–C, and Supplemental Figure 1, A and B; supplemental material available online with this article; https://doi.org/10.1172/JCI190552DS1), and with α_5_ integrin (Supplemental Figure 1, C–E).

Because ANGPTs bind TIE2 receptors across EC-EC junctions in *trans* (17, 18), we analyzed TIE2 and β_1_-integrin colocalization in LECs secreting endogenous ANGPT2 and in ECs stimulated with recombinant human Ang2 (rhAng2; R&D) or COMP-Ang1, a previously characterized recombinant ANGPT1 (35). Interestingly, both ANGPT1- and ANGPT2-induced TIE2 colocalized with inactive β_1_-integrin in EC-EC junctions, suggesting potential receptor crosstalk (Figure 1, D–H, and Supplemental Figure 1, F–K).

Because ANGPT2 is known to activate β_1_-integrin (21, 22), we further assessed the compartmentalization of ANGPT2 and TIE2 with the active and inactive β_1_-integrin conformers in ECs, using a proximity ligation assay (PLA) (36), which can detect proteins less than 40 nm from each other. We used antibodies that recognize ANGPT2 and the ectodomains (ECDs) of TIE2 and β_1_-integrin. In particular, whereas mAb13 detects β_1_-integrin in its inactive conformation, 9EG7 selectively recognizes the active β_1_-integrin conformation (Figure 1I). PLA signal was detected specifically with TIE2 and mAb13 antibodies, but not with TIE2 and 9EG7 antibodies (Figure 1, J and K), indicating preferential interaction of TIE2 with inactive rather than active β_1_-integrin. However, ANGPT2 interaction was detected with both inactive and active β_1_-integrin using PLA (Figure 1, L–O).

To determine whether ANGPT2 and TIE2 regulate junctional β1-integrin localization, we silenced them using shRNAs. Both shANGPT2 and shTIE2 reduced junctional inactive β_1_-integrin. shTIE2 further increased active β_1_-integrin in ECM adhesions while decreasing CDH5 (Supplemental Figure 2), consistent with ANGPT2-mediated β_1_-integrin activation in the absence of TIE2, as previously reported (21, 22). Taken together, ANGPT-induced TIE2 colocalizes with inactive β_1_-integrin in EC junctions, whereas ANGPT2, in the absence of TIE2, is additionally found near active β_1_-integrin.

### Junctional TIE2 phosphorylation is reduced by β_1_-integrin deletion and during endotoxemia.

**Figure 2:**
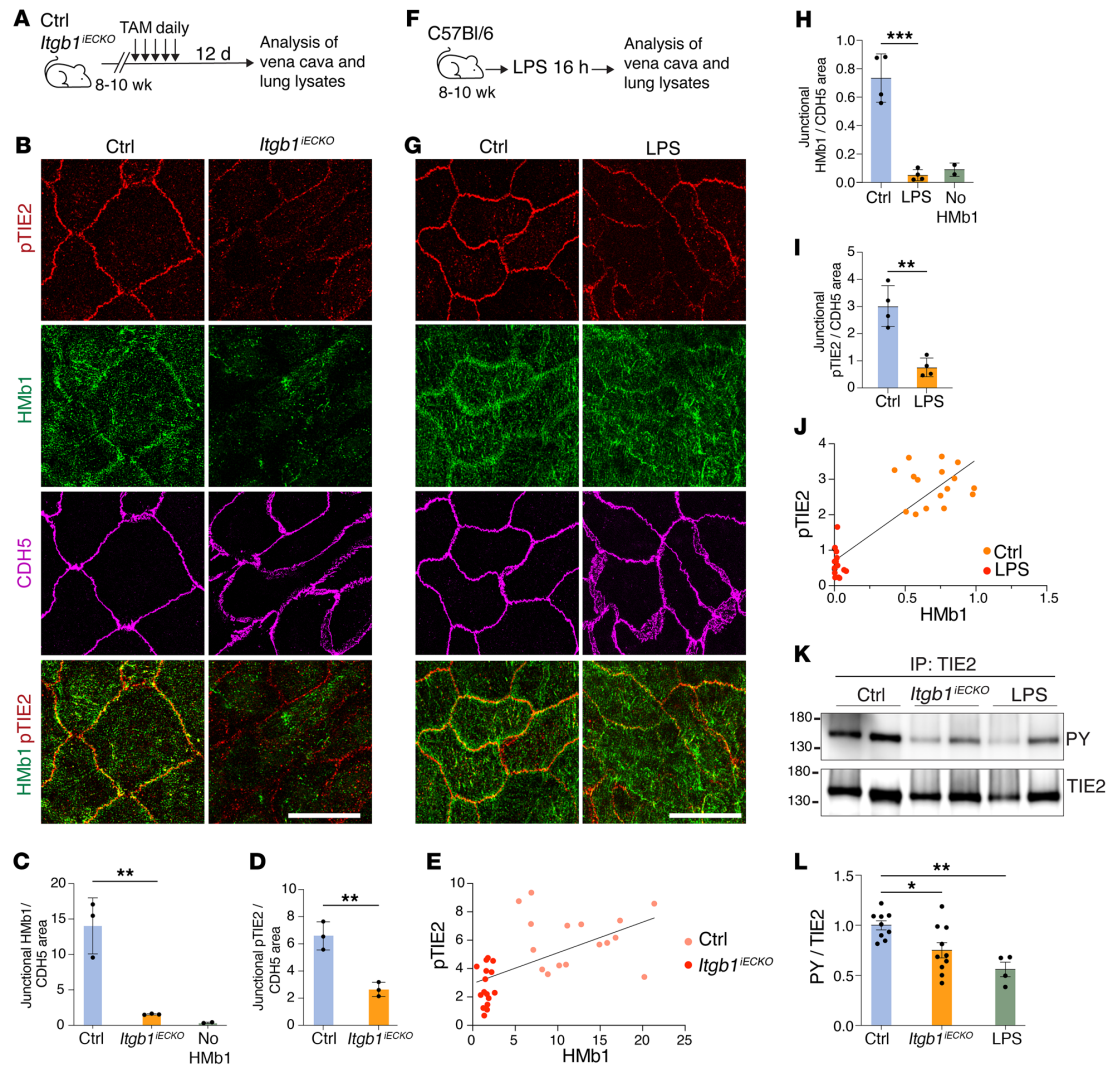
Regulation of phosphorylated TIE2 and β_1_-integrin in cell junctions in *Itgb1^iECKO^* mice and in endotoxemia. (**A** and **B**) Vena cava from *Itgb1^iECKO^* and control (Ctrl) mice were stained for β_1_-integrin (HMb1), pY992-TIE2 (pTIE2), and VE-cadherin (CDH5). Larger fields of view are shown in Supplemental Figure 3A. (**C** and **D**) Quantification of junctional HMb1 (**C**) and junctional pTIE2 (**D**) normalized to CDH5. *n* = 3 mice per group. (**E**) Correlation of junctional pTIE2 with HMb1 in Ctrl and *Itgb1^iECKO^* mice per image. There was a positive correlation between pTIE2 and HMb1 (Pearson’s *r* = 0.590 [95% CI 0.303–0.778]; *n* = 32 images; 2-tailed *P* = 0.0004). (**F** and **G**) Vena cava from control mice and after LPS administration for 16 hours were stained for inactive β_1_-integrin (HMb1), pTIE2, and CDH5. Larger fields of view are shown in Supplemental Figure 3E. (**H** and **I**) Quantification of junctional HMb1 (**H**) and junctional pTIE2 (**I**) normalized to CDH5. *n* = 4 mice per group. (**J**) Correlation of junctional pTIE2 vs. HMb1. There was a strong positive correlation between pTIE2 and HMb1 (Pearson’s *r* = 0.877 [95% CI 0.763–0.938]; *n* = 33; 2-tailed *P* < 0.0001). (**K** and **L**) Western blot for phosphotyrosine and TIE2 after TIE2 immunoprecipitation from lung lysates from control and *Itgb1^iECKO^* mice and from control mice after LPS administration for 16 hours (**K**). Quantification of phosphorylated TIE2/total TIE2, normalized to control (Ctrl) (**L**). *n* = 9, 10 (*Itgb1^iECKO^*), and 4 (LPS) mice per group. Scale bars: 20 μm (**B** and **G**). Confocal Z-stack projections are shown. Statistical tests were as follows: 2-tailed *t* test (**C**, **D**, **H**, and **I**); 1-way ANOVA with Tukey’s post hoc test (**L**); and Pearson’s correlation (**E** and **J**). Data are presented as mean ± SD (**C**, **D**, **H**, and **I**)and ± SEM (**L**). *P < 0.05, **P < 0.01, ***P < 0.001. Tamoxifen, TAM.

The junctional enrichment and colocalization of β_1_-integrin with TIE2 in cultured ECs prompted us to examine their distribution in vivo. Therefore, we analyzed whole-mount preparations of mouse inferior vena cava, in which tyrosine-992–phosphorylated TIE2 (pY992-TIE2) has been shown to localize to endothelial junctions, indicating homeostatic junctional TIE2 activation (37). Consistently, pY992-TIE2 was readily detected at EC junctions, and β_1_-integrin was enriched at junctional regions (Figure 2, B and G, and Supplemental Figure 3, A, B, and E), using the function-blocking β_1_-integrin antibody HMb1 (38).

We hypothesized that β_1_-integrin in junctions might regulate TIE2 and, therefore, deleted endothelial β_1_-integrin (*Itgb1*) in adult mice (*Itgb1^iECKO^*) (Figure 2A). As expected, β_1_-integrin was decreased in vena cava and, notably, total TIE2 and pY992-TIE2 were decreased in *Itgb1^iECKO^* mice (Figure 2, B–E, and Supplemental Figure 3, A–D). Phosphorylated TIE2 was also slightly reduced in lung lysates from *Itgb1^iECKO^* mice when compared with controls, suggesting decreased homeostatic TIE2 activity in *Itgb1^iECKO^* mice (Figure 2, K and L).

Given that endothelial β_1_-integrin deletion impairs TIE2 phosphorylation in vivo, we next examined whether inflammatory suppression of TIE2 signaling is associated with altered junctional β_1_-integrin. Reduced TIE2 activity during endotoxemia has been linked to compromised junctional stability (39, 40). Accordingly, mice were administered LPS for 16 hours (Figure 2F). Consistent with previous reports, TIE2 phosphorylation was reduced in lung lysates, and additionally, at vena cava junctions (Figure 2, G–L, and Supplemental Figure 3E). Notably, junctional β_1_-integrin was also reduced and closely correlated with pY992-TIE2 (Figure 2, H and J). Both endotoxemic and *Itgb1^iECKO^* mice had widened, serrated CDH5-positive junctions compared with controls (Figure 2, B and G, and Supplemental Figure 3, A and E), indicative of impaired barrier integrity, as previously reported in the retina of newborn *Itgb1^iECKO^* mice (41).

In the lymphatic vasculature, β_1_-integrin expression was low along dermal capillaries and enriched at lymphatic valves, limiting quantitative analysis of junctional localization in vivo (Supplemental Figure 3F). We therefore used LEC cultures to assess β_1_-integrin regulation during junctional destabilization. Thrombin stimulation disrupted the continuous CDH5 junctional pattern and reduced colocalization of inactive β_1_-integrin with TIE2 (Supplemental Figure 4, A–C). These findings indicate that β_1_-integrin is enriched at venous EC junctions in vivo, correlating with phosphorylated TIE2, and that junctional β_1_-integrin decreases during inflammation-induced endothelial destabilization.

### Oligomeric ANGPT2 activates TIE2/FOXO1 signaling facilitated by inactive β_1_-integrin.

**Figure 3:**
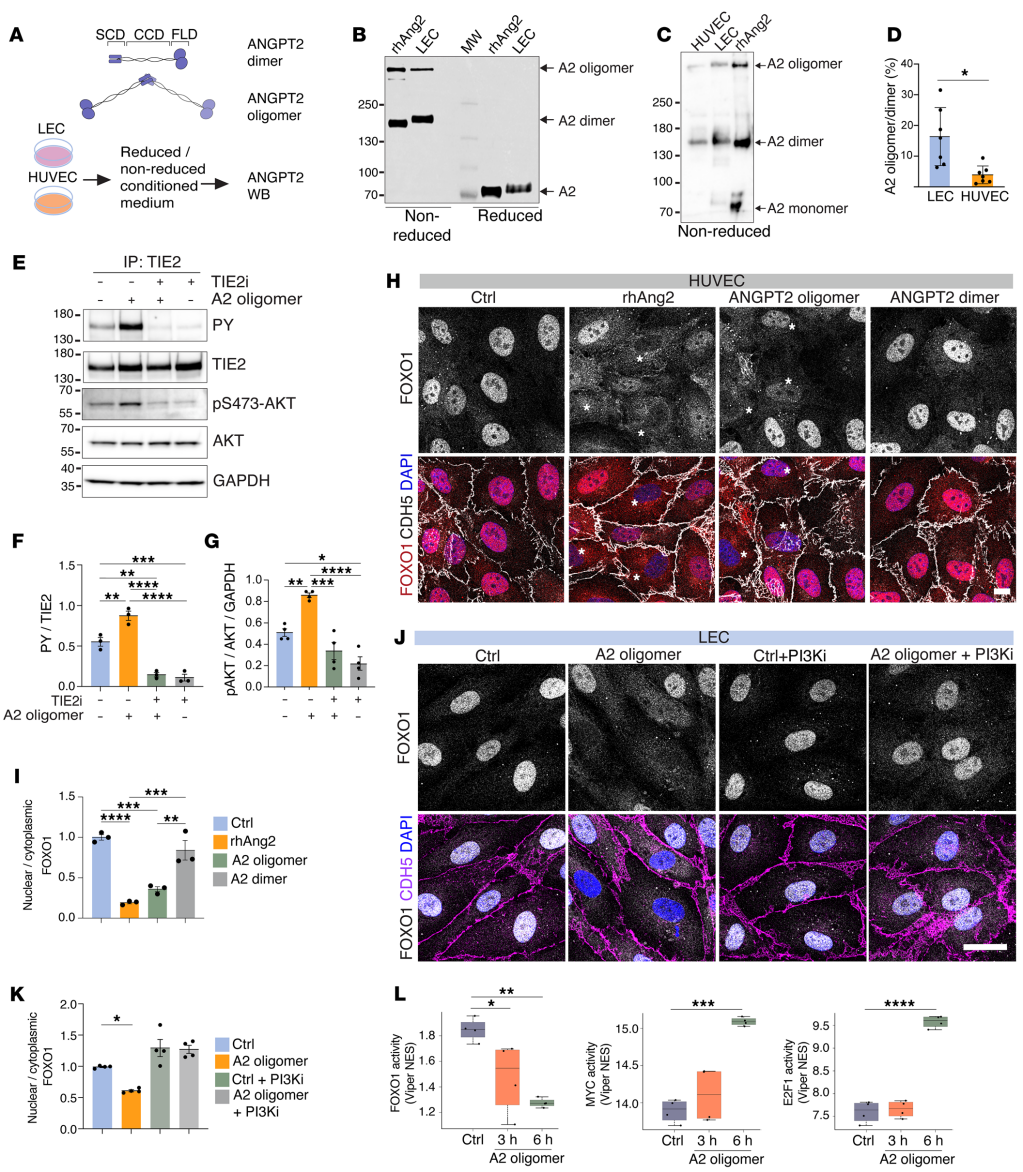
Oligomeric ANGPT2 induces TIE2/FOXO1 signaling in ECs. (**A**) Schematic representation of analysis of ANGPT2 dimers and oligomers in cell culture medium. (**B**–**D**) Representative immunoblots and quantification of oligomeric and dimeric ANGPT2 in 10× concentrated LEC- (**B**–**D**) and HUVEC-conditioned medium (**C** and **D**) under nonreducing and reducing conditions; rhAng2 was used as a control. *n* = 7 independent experiments. (**E**–**G**) Representative immunoblots (**E**) and quantification of phosphorylated (PY) and total TIE2 following immunoprecipitation from LECs stimulated with ANGPT2 (A2) oligomer ± TIE2 inhibitor (TIE2i, BAY-826) (*n* = 3 independent experiments) (**F**), and phospho-Ser473 AKT in whole-cell lysates from the same conditions (*n* = 4 independent experiments) (**G**). (**H** and **I**) Staining of HUVECs stimulated with oligomeric or dimeric ANGPT2 purified from CHO cells, or rhAng2, for CDH5 and FOXO1 (**H**). Quantification of nuclear/cytoplasmic FOXO1 ratio (**I**). *n* = 3 independent experiments. Asterisks mark cells with decreased nuclear FOXO1. (**J** and **K**) Staining of FOXO1 and VE-cadherin (CDH5) in LECs treated with A2 oligomer in the presence of PI3Ki (LY294002) or control (Ctrl) (**J**). Quantification of nuclear vs cytoplasmic FOXO1 ratio (**K**). (**L**) Bulk mRNA sequencing of LECs stimulated with A2 oligomer for 3 or 6 hours. Inferred transcription factor activity is shown as normalized VIPER enrichment scores (NES) for FOXO1, MYC, and E2F1 across treatments. Scale bars: 10 μm (**H**), 25 μm (**J**). Two-tailed unpaired *t* test (**D**), 1-way ANOVA with Tukey’s post hoc test (**F**, **G**, **I**, and **K**). NES values were compared across conditions by 1-way ANOVA with Tukey’s multiple comparison, and with Benjamini-Hochberg FDR correction across transcription factors (**L**). Data are presented as mean ± SEM. *P < 0.05, **P < 0.01, ***P < 0.001, ****P < 0.0001.

To investigate how junctional β_1_-integrin regulates ligand-induced TIE2 signaling, we focused on ANGPT2, which is endogenously expressed by both LECs and venous ECs (42) and, therefore, provides a suitable model for studying endogenous ANGPT2/TIE2 signaling. ANGPTs form coiled-coil domain–mediated (CCD-mediated) dimers that assemble into higher-order oligomers through the SCD (Figure 3A). Whereas ANGPT1 oligomers potently activate TIE2, ANGPT2 can exist as dimers, contributing to weaker TIE2 activation (43). Therefore, we examined ANGPT2 oligomerization in EC-conditioned medium under reducing and nonreducing conditions (Figure 3, A–D). Both dimeric and oligomeric ANGPT2 were detected in LECs and in HUVECs; however, ANGPT2, and particularly its oligomeric form, was enriched in LECs when compared with HUVECs (Figure 3, C and D). rhAng2 migrated as a mixture of oligomers, dimers, and monomers (Figure 3, B and C), in line with a previous report (43).

To test whether ANGPT2 abundance influences oligomerization, ANGPT2 expression was increased in HUVECs, using a doxycycline-inducible, constitutively active FOXO1 mutant (44) that potently induces *ANGPT2* transcription. Treating transduced cells with doxycycline elevated *ANGPT2* mRNA and protein levels, and increased the fraction of oligomeric ANGPT2, suggesting that ANGPT2 oligomerization is partly influenced by overall ANGPT2 abundance (Supplemental Figure 4, D–F).

To investigate the functions of dimeric versus oligomeric ANGPT2, we expressed ANGPT2 in CHO cells, purified the distinct species, and, using size-exclusion chromatography with multi-angle light scattering (SEC-MALS), determined their molecular weights, which corresponded to a 141 kDa dimer and a 383 kDa oligomer (potentially a glycosylated tetramer; Supplemental Figure 4G and Supplemental Tables 1 and 2). Oligomeric ANGPT2 induced TIE2 and downstream AKT phosphorylation in LECs, which were abolished by TIE2-inhibitor treatment (BAY-826) (Figure 3, E–G). Consistently, oligomeric, but not dimeric, ANGPT2 decreased nuclear FOXO1 in HUVECs and in LECs, an effect blocked by PI3K inhibition (LY294002) (Figure 3, H–K). Thus, oligomeric ANGPT2 served as a TIE2 agonist, signaling through PI3K/AKT to decrease nuclear FOXO1.

To define the direct downstream effects of oligomeric ANGPT2, we performed bulk RNA-Seq in LECs 3 and 6 hours after stimulation with ANGPT2 oligomer and compared responses with rhAng2 (Supplemental Figure 5). The number of differentially expressed genes (DEGs) increased at 6 hours and oligomeric ANGPT2 induced more DEGs than did rhAng2 (*P* < 0.01; log_2_ fold change > 0.5) (Supplemental Figure 5, A and E). Consistent with FOXO1 inhibition, DEGs induced by oligomeric ANGPT2 overlapped with genes downregulated by constitutively active FOXO1 in HUVECs (42) (*P* < 0.00001) (Supplemental Figure 5B). Virtual Inference of Protein-Activity by Enriched Regulon (VIPER) analysis indicated reduced FOXO1 activity following ANGPT2 stimulation, whereas inferred activity of MYC, a FOXO1-repressed target (45) as well as the E2F1 transcription factor, were increased (Figure 3L and Supplemental Figure 5, C and F). Gene set enrichment analysis further supported an ANGPT2-induced shift toward cell proliferation (Supplemental Figure 5, D and G). Together, these data indicate that oligomeric ANGPT2, enriched in LECs, can act as a TIE2 agonist to suppress FOXO1 activity and induce a proliferative transcriptional signature in LECs.

**Figure 4:**
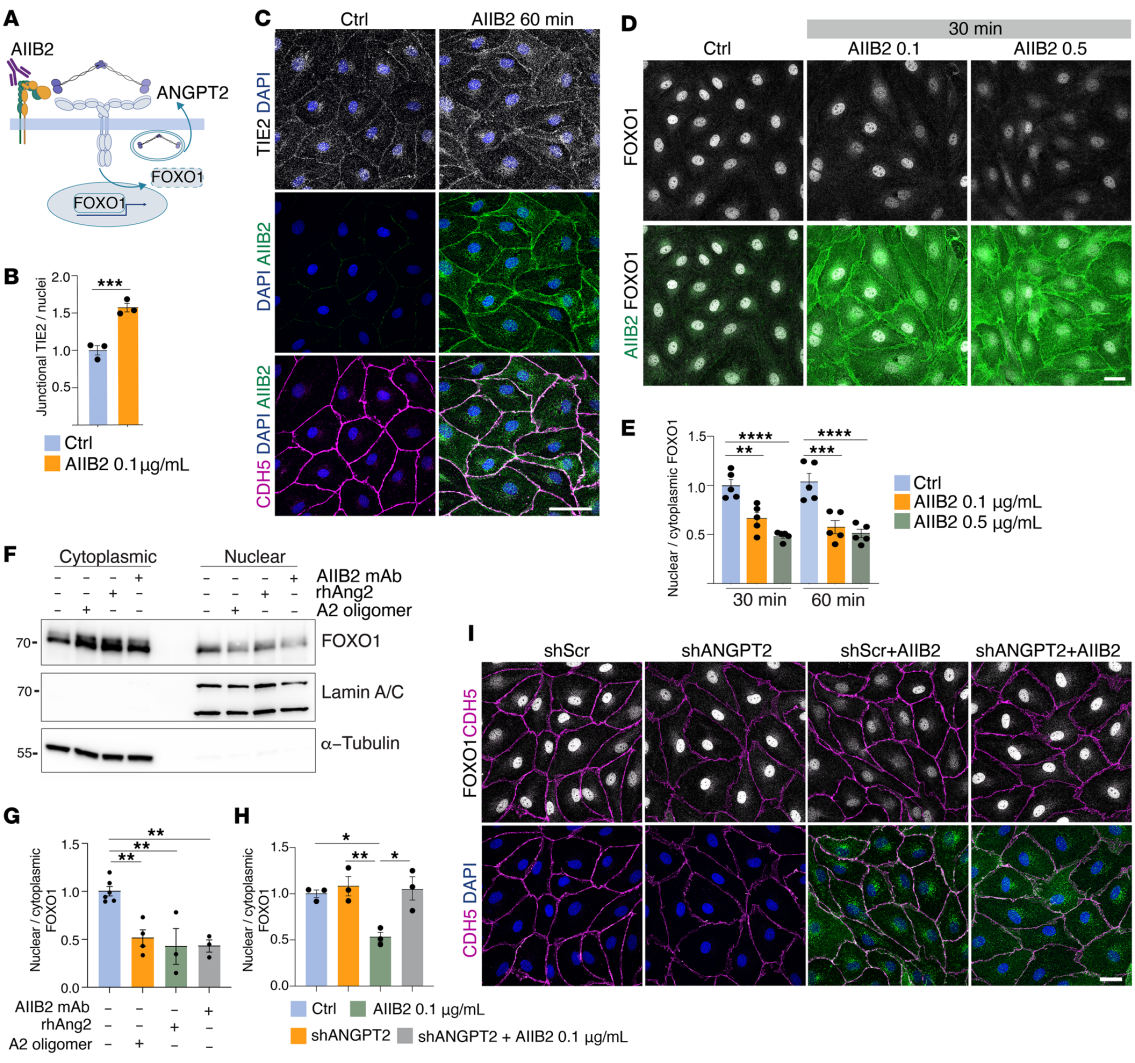
Inactive β_1_-integrin facilitates ANGPT2/TIE2/FOXO1 signaling in LECs. (**A**) Schematic representation of ANGPT2/TIE2/FOXO1 pathway and AIIB2 antibody recognizing non–ECM-bound β_1_-integrin. (**B** and **C**) LECs stimulated with control or AIIB2 antibody for 60 minutes were stained for surface TIE2, CDH5, and AIIB2 (using secondary antibodies) (**C**). Quantification of junctional TIE2 normalized to number of nuclei (**B**). (**D** and **E**) LECs were stimulated with AIIB2 antibody for 30 and 60 minutes and stained for FOXO1 and AIIB2 (see Supplemental Figure 6A for staining at 60 minutes) (**D**). Quantification of nuclear/cytoplasmic FOXO1 ratio normalized to nuclei (**E**). (**F** and **G**) LECs were stimulated with ANGPT2 (A2) oligomer, rhAng2, or AIIB2 antibody and FOXO1 were analyzed using immunoblotting from nuclear and cytoplasmic fractions, using lamin A/C and α-tubulin markers, respectively. (**H** and **I**) shANGPT2- and shScr-treated LECs were stimulated with AIIB2 antibody for 60 minutes and stained for FOXO1, TIE2, and AIIB2. Nuclear/cytoplasmic FOXO1 ratio was quantified normalized to nuclei. *n* = 3 (**B**, **E**, **G** [*n* = 5 for control, *n* = 4 for A2 oligomer], and **H**) or *n* = 5 (**E**) independent experiments. Statistical tests included 2-tailed *t* test (**B**) and 1-way ANOVA with Tukey’s post hoc test (**E**, **G**, and **H**). Data are presented as mean ± SEM. Scale bars: 50 μm (**C**), 25 μm (**D** and **I**). Confocal Z-stack projections are shown. *P < 0.05, **P < 0.01, ***P < 0.001, ****P < 0.0001.

To assess the necessity of ECM ligand binding for junctional localization of β_1_-integrin, we treated LECs with the β_1_-integrin monoclonal antibody AIIB2, which competes with ligand binding, recognizing nonligand-bound β_1_-integrins (46). AIIB2 localized to cell-cell junctions when incubated with live LECs at +37°C, indicating ligand-unbound β_1_-integrin in junctions (Figure 4, A and C). Interestingly, AIIB2 treatment increased junctional TIE2 (Figure 4, B and C) and decreased nuclear FOXO1 in LECs (Figure 4, D–G, and Supplemental Figure 6A), whereas this effect was lost upon shANGPT2 silencing or TIE2 inhibition (BAY-826) (Figure 4, H and I, and Supplemental Figure 6, B and C), suggesting that β_1_-integrin inhibition enhances junctional ANGPT2/TIE2 signaling and decreases nuclear FOXO1. Although β_1_-integrin silencing also reduced nuclear FOXO1, it attenuated ANGPT2-induced reduction in nuclear FOXO1 (Supplemental Figure 6, D and E), supporting a role for β_1_-integrin in ANGPT2/FOXO1 signaling.

### ANGPT2 engages α_5_β_1_-integrin through its N- and C-terminal domains.

**Figure 5:**
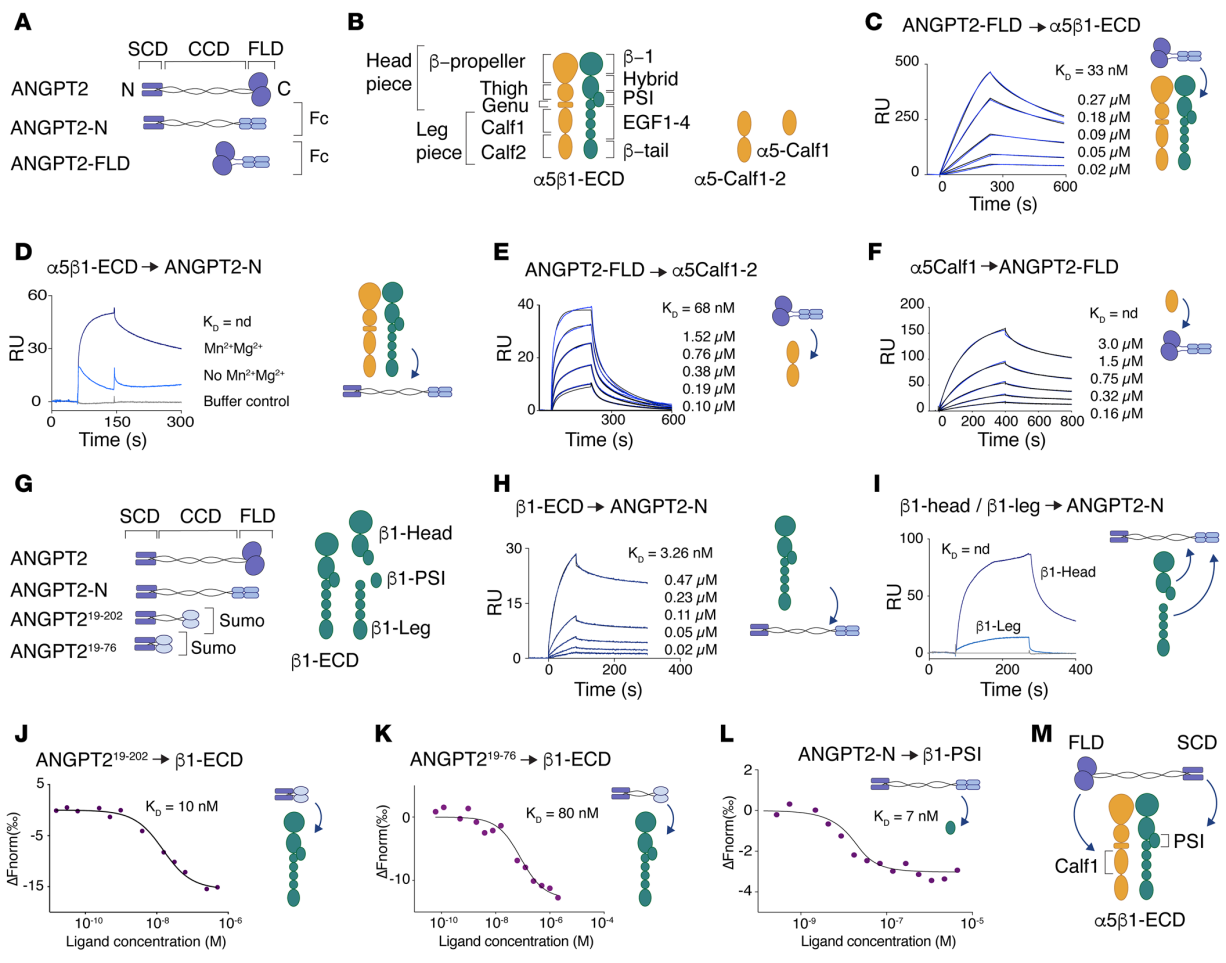
Direct interactions of α_5_β_1_-integrin with N- and C-terminal ANGPT2 domains. (**A** and **B**) Schematic representation of ANGPT2 (**A**) and α_5_β_1_-ECD (**B**) recombinant proteins used. (**C**) Binding of ANGPT2-FLD-Fc to immobilized α_5_β_1_-ECD in SPR. Two-state reaction kinetics were fitted to the original data. (**D**) Binding of α_5_β_1_-ECD (1.3 μM) to immobilized ANGPT2-N-Fc in SPR in the presence and absence of Mn^2+^ and Mg^2+^. (**E**) Binding of ANGPT2-FLD-Fc to immobilized α_5_Calf1-2 in SPR. (**F**) Binding of α_5_Calf1 to immobilized ANGPT2-FLD-Fc in SPR. (**G**) Schematic representation of N-terminal ANGPT2 and β_1_-ECD constructs. (**H**) Binding of β_1_-ECD to immobilized ANGPT2-N-Fc in SPR. (**I**) Binding of β_1_-head and -leg piece (β_1_-leg) to immobilized ANGPT2-N-Fc in SPR. (**J** and **K**) Binding of ANGPT2^19–202^–SUMO (**J**) and ANGPT2^19–76^-SUMO (**K**) to fluorescently labelled β_1_-ECD (2 nM) in MST. (**L**) Binding of ANGPT2-N-Fc to fluorescently labelled β_1_-PSI (20 nM) in MST. (**M**) Schematic representation of ANGPT2 binding through FLD and SCD domains to Calf1 and PSI domains of α_5_ and β_1_-integrins, respectively. Representative experiments with average K_D_ are shown. *n* = 3 independent experiments (**C**–**F** and **H**–**L**). Nd, not determined.

These results prompted us to ask whether the effects of inactive β_1_-integrin on ANGPT/TIE/FOXO1 signaling reflect a direct ligand–integrin interaction, leading us to investigate the interaction of ANGPT2 with α_5_β_1_-integrin. Using rhAng2, we detected its binding to the ECD of α_5_β_1_-integrin (α_5_β_1_-ECD) in surface plasmon resonance (SPR) (Supplemental Figure 7A). To further define this interaction, we generated a panel of recombinant ANGPT2 and α_5_β_1_-integrin proteins (Supplemental Table 1 and Supplemental Figure 8). We first generated the N- and C-terminal parts of ANGPT2 as human IgG Fc fusion proteins to induce dimerization, mimicking the native coiled-coil–mediated dimers of the CCD/SCD (ANGPT2-N-Fc) and FLD (ANGPT2-FLD-Fc) (Figure 5, A and B). Notably, both ANGPT2 fragments bound α_5_β_1_-ECD in SPR (Figure 5, C and D), indicating that α_5_β_1_-integrin engages both the ANGPT2 N- and C-terminal domains.

### α_5_-Integrin Calf1 and the β_1_-integrin headpiece mediate ANGPT2 binding.

Because both the FLD and the N-terminal CCD/SCD have been implicated in ANGPT2 signaling (22, 47), we next delineated the α_5_β_1_-integrin regions responsible for ANGPT2 binding. The binding of ANGPT2 FLD to α_5_β_1_-ECD was independent of fibronectin binding, suggesting interaction outside of the α_5_β_1_-integrin headpiece, and FLD also did not bind β_1_-integrin ECD (β_1_-ECD) (Supplemental Figure 7, B and C). Therefore, we assessed FLD binding to the membrane-proximal α_5_-integrin leg, consisting of the Calf1-2 domains. The isolated Calf1-2 domain of α_5_ integrin (α_5_Calf1-2) bound ANGPT2-FLD-Fc with a *K_d_* of 50–90 nM (Figure 5E). Because ANGPT2-FLD-Fc forms dimers through the Fc region, we cleaved it proteolytically and found that the monomeric FLD retained binding to α_5_Calf1-2 (Supplemental Figure 7D). Moreover, we created the isolated α_5_Calf1 monomer, which bound to ANGPT2 FLD (Figure 5F), whereas α_5_Calf2 did not (Supplemental Figure 7, E and F), narrowing the ANGPT2 FLD interaction to Calf1 of α_5_ integrin. The highly homologous ANGPT1 FLD also bound to α_5_Calf1-2 (Supplemental Figure 9, A and B).

Because we previously found that the ANGPT2, but not ANGPT1, N-terminus (CCD/SCD) activates α_5_β_1_-integrin (22), we further characterized ANGPT2-N-Fc binding and found a high-affinity interaction with the β_1_-integrin ECD (β_1_-ECD) (*K_d_* 2-5 nM) (Figure 5, G and H) and with the isolated β_1_-integrin headpiece (β_1_-head) but not the leg piece (Figure 5I). ANGPT1-N-Fc did not bind β_1_-ECD under similar conditions (Supplemental Figure 9, C and D).

To narrow down the binding site, we created N-terminal ANGPT2 fragments, encompassing the SCD (ANGPT2^19–76^) and also parts of CCD (ANGPT2^19–202^), and attached a C-terminal monomeric SUMOStar tag to increase their solubility (Supplemental Figures 8J and 9E). ANGPT2^19–76^ and ANGPT2^19–202^ bound to the β_1_-ECD with affinity of *K_d_* 80 nM and 10 nM, respectively (Figure 5, J and K), suggesting that the ANGPT2 SCD (ANGPT2^19–76^) is sufficient for binding to β_1_-ECD, but additional amino acids (ANGPT2^19–202^) contribute to high affinity binding. Furthermore, we precisely localized the ANGPT2 interaction to the PSI domain within the β_1_-ECD (Figure 5L). In conclusion, the ANGPT1 and ANGPT2 FLD bind to α_5_-integrin Calf1 domain, whereas the SCD of ANGPT2, but not of ANGPT1, binds to the β_1_-integrin PSI domain (Figure 5M).

### Structural and MD analyses delineate the ANGPT2–α_5_-integrin interaction interface.

Having identified the ANGPT2 regions that engage α_5_β_1_-integrin, we next sought to define their interaction at atomic resolution. To investigate how ANGPT2 FLD interacts with α_5_ integrin, we assessed an ANGPT2-derived peptide (corresponding to residues E352–Q366 in human ANGPT2), previously reported to bind α_5_ integrin (47). When added in a molar excess, the peptide inhibited binding of ANGPT2 FLD to α_5_Calf1-2 (Supplemental Figure 10A), suggesting it forms part of the binding interface.

**Figure 6:**
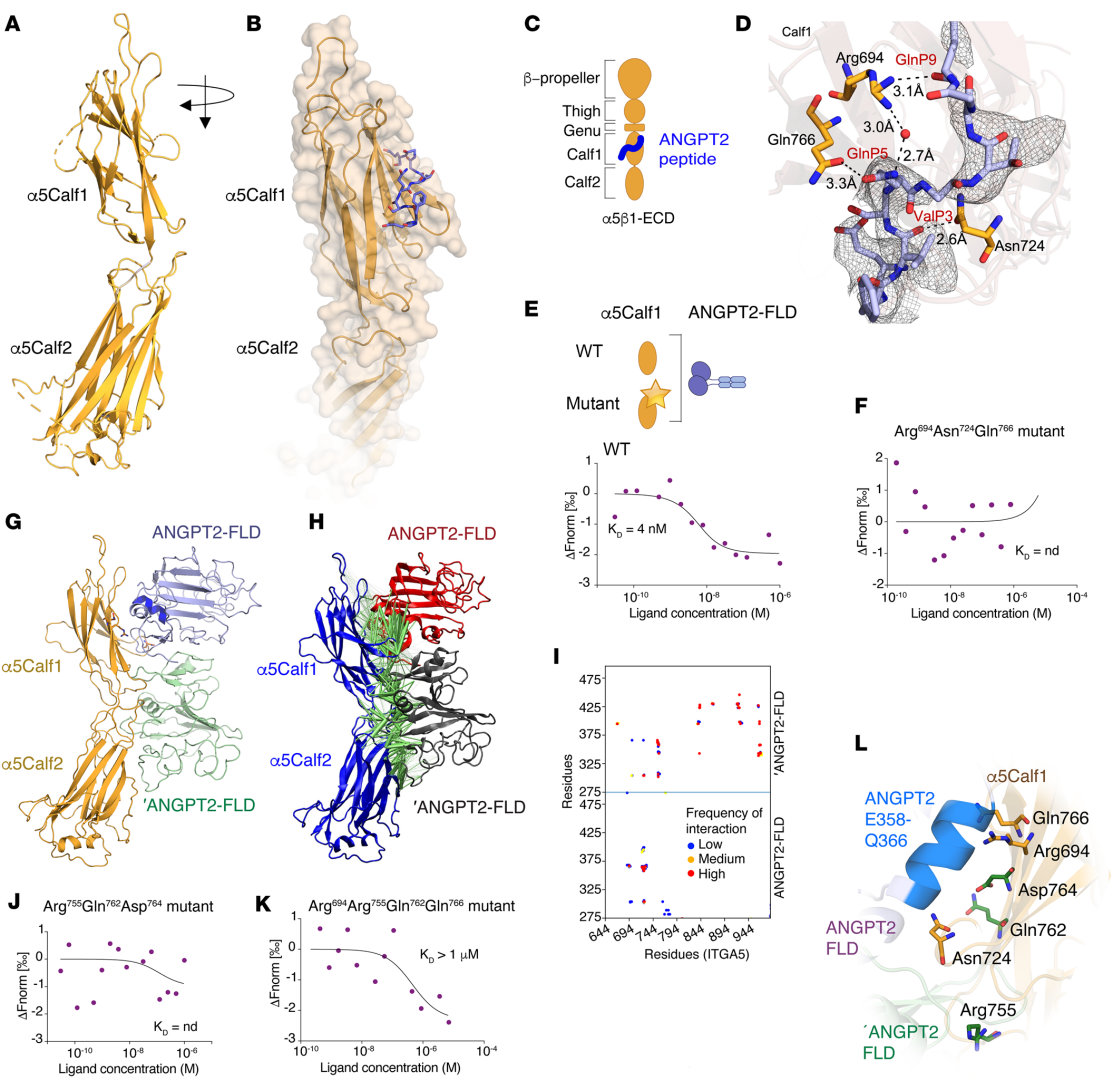
Crystal structure and MD simulations reveal the interface of ANGPT2 FLD–α_5_Calf1-2 complex. (**A**) Crystal structure of apo α_5_Calf1-2. (**B**) Crystal structure of α_5_Calf1-2 in complex with the ANGPT2 peptide (blue) [residue E358–Q366, P(1)–P(9)]. (**C**) Schematic representation of ANGPT2 peptide (blue) bound to α_5_Calf1. (**D**) Electron density of the ANGPT2 peptide bound to α_5_Calf1-2 visible in the co-crystal structure in (**B**). The peptide is seen interacting with several residues in Calf1; see text for details. (**E** and **F**) Binding of WT (**E**) and mutant α_5_Calf1 (Arg694Trp, Asn724Val, Gln766Glu) (**F**, no binding is detected) to fluorescently labelled ANGPT2-FLD-Fc in MST. (**G**) An optimized docked model of an ANGPT2-FLD dimer in complex with α_5_Calf1-2. Residue E358–Q366, corresponding to the ANGPT2 peptide used in the crystal structure in (**B**), is indicated in bright blue. (**H** and **I**) Atom-scale MD simulation of ANGPT2-FLD dimer in complex with α_5_Calf12 (*n* = 3). Thickness of the line indicates frequency of interaction (**H**). Heatmap of interaction frequencies of amino acid residues of α_5_Calf1-2 (*x* axis) and ANGPT2-FLD dimer (*y* axis) from MD simulation (**I**). (**J** and **K**) Binding of mutant α_5_Calf1 (Arg755Glu, Gln762Ala, Asp764Asn (**J**) and Arg694Trp, Arg755Glu, Gln762Ala, Gln766Glu) (**K**) to fluorescently labelled ANGPT2-FLD-Fc in MST. No binding is detected in (**K**), and binding is decreased to a micromolar scale in (**J**) as compared with wt in (**E**). (**L**) Substituted amino acid residues shown in a close-up of the docked model in **G**. *n* = 3 independent experiments (**E**, **F**, **J**, and **K**).

Therefore, we crystallized the α_5_Calf1-2 domain, which had not been reported previously to our knowledge, and its complex with the 9-amino acid ANGPT2 peptide ligand Glu (P1)-Gln(P9), corresponding to E358–Q366 (Figure 6, A and B, and Supplemental Table 3). The structure of the unliganded α_5_Calf1-2 at 1.92 Å resolution showed that Calf1 (residues 644–787) and Calf2 domains (residues 792–989) both form immunoglobulin-like β sandwich folds separated by a linker (residues 788–791), similar to Calf domains of other α-integrins (48) (Figure 6A and Supplemental Figure 10B).

The structure of the α_5_Calf1-2–ANGPT2 peptide complex was solved by molecular replacement using the unliganded α_5_Calf1-2 structure (Figure 6, B–D). In the resulting complex structure, the electron density for 6 residues of the ANGPT2 peptide was well defined (Figure 6D). This density was also present in a simulated omit map, generated by excluding the peptide from the model, confirming that the observed density is not model biased (Figure 6D).

The peptide interacts with the β sheet that is formed by β-strands 4, 6, 7, 9, and 10 in the Calf1 domain, fitting in a cavity surrounded by loops between β-strands 6 and 7. The structure revealed that Gln(P5), which corresponds to Gln362 in ANGPT2, a residue previously reported to be crucial in mediating binding to α_5_ integrin (47), interacts with the OH-group of Gln766 in α_5_Calf1 (Figure 6D). The NH groups of Gln P5 and of Arg694 in α_5_Calf1 form a hydrogen bond with a water molecule located at the interface (Figure 6D). Additionally, Gln(P9) forms a hydrogen bond with the NH group of Arg694 in α_5_Calf1, and the carboxyl groups of Phe(P2) and Val(P3) sandwich the side chain of Asn724 in α_5_Calf1 (Figure 6D).

To confirm the binding interface, we introduced 3 amino acid substitutions in the binding site of the ANGPT2 peptide in α_5_Calf1 at positions identified in the crystal structure (Arg694Trp, Asn724Val, and Gln766Glu) (Figure 6, E and F). The substitutions were predicted to interfere with ANGPT2 binding while preserving the overall fold of α_5_Calf1 when tested by short MD simulations (Supplemental Table 4) and circular dichroism analysis (Supplemental Figure 11A). Wild-type α_5_Calf1 bound to ANGPT2-FLD with 4 nM affinity in microscale thermophoresis (MST), whereas binding of the mutant was undetectable (Figure 6, E and F). These results validate the α_5_Calf1 surface identified in our crystal structure as the binding site for ANGPT2-FLD (Figure 6, B and D).

Having identified the binding site in α_5_Calf1 for ANGPT2, we investigated how the full ANGPT2 FLD and α_5_Calf1-2 domains interact, using molecular modeling and atom-scale MD simulations. For these, we docked dimeric ANGPT2 FLD structure (13) with our α_5_Calf1-2 crystal structure, preserving the contacts observed in the ANGPT2 peptide–α5Calf1-2 co-crystal complex (Figure 6G, Supplemental Video 1, and Supplemental Methods). The resulting model revealed an extended interaction interface between α_5_Calf1 and ANGPT2, involving residues from both proteins (Figure 6G). In addition to the helical region corresponding to the ANGPT2 peptide (residues E358–Q366), a β-hairpin on the ANGPT2 FLD (residues F302–K309) interacts with a β-hairpin on α_5_Calf1 (residues A722–V729), and a short N-terminal β-strand of ANGPT2 FLD (residues I279–283R) interacts with a β-strand on α_5_Calf1 (residues T760–F765). The second protomer also forms a shape complementary to α_5_Calf1-2.

Upon subjecting the dimeric ANGPT2 FLD–α_5_Calf1-2 complex to atomistic MD simulations (*n* = 3 repeats of 1 μs each), a slight separation and repositioning of the ANGPT2 monomers (Figure 6, H and I) was observed. However, both protomers (ANGPT2 FLD and ′ANGPT2 FLD) retain their essential interactions with α_5_Calf1, including the region covering the ANGPT2 peptide (residues E358–Q366). We also noted additional interactions of the second ′ANGPT2 FLD protomer with Calf2, which may facilitate binding (Figure 6, H and I). Because Calf2 alone was not sufficient to mediate binding to ANGPT2 FLD (Supplemental Figure 7, E and F), we focused on Calf1. To experimentally validate the model, we introduced amino acid substitutions at Calf1 residues predicted to mediate ANGPT2 binding, identified using MD simulations (Arg755Glu, Gln762Ala, and Asp764Asn) and both simulations and crystal structure (Arg694Trp, Arg755Glu, Gln762Ala, and Gln766Glu) (Figure 6, J–L). The mutants retained their structure as confirmed by circular dichroism spectroscopy and MD simulation analyses (Supplemental Figure 11A and Supplemental Table 4) but showed a reduction of ANGPT2 FLD binding affinity in MST assays compared with wild-type Calf1 (Figure 6, J and K). Together, these results support the structural model of the α_5_Calf1–ANGPT2 FLD complex and demonstrate that the interaction interface identified by crystallography, MD simulations, and mutagenesis (Figure 6) is functionally relevant. Moreover, conservation of this interface among several α integrins suggests broader relevance of this interaction mode (Supplemental Figure 11B).

### ANGPT2 SCD binds extended β_1_-integrin.

**Figure 7:**
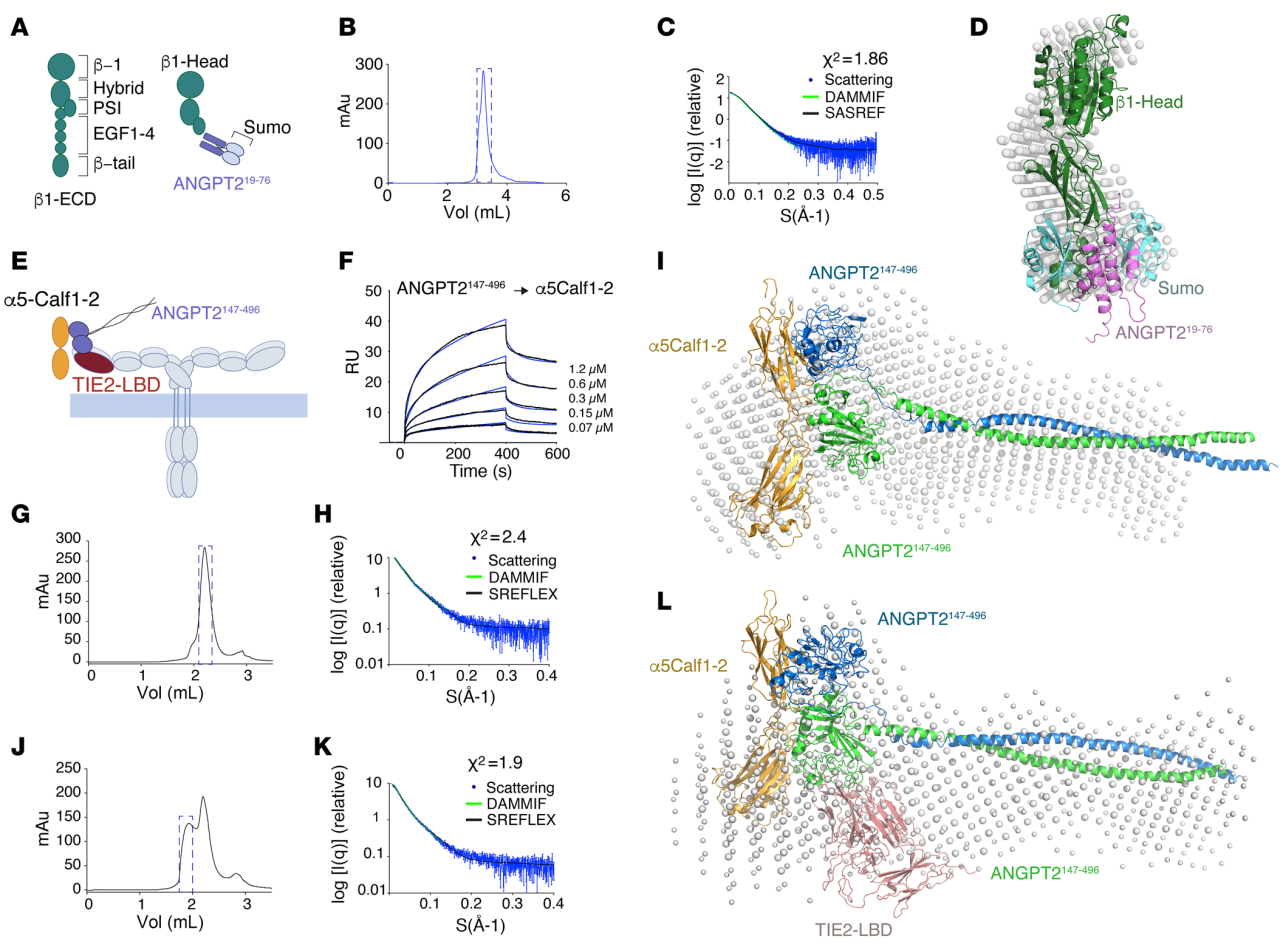
SAXS models of N-terminal and C-terminal ANGPT2 complexes with α_5_ and β_1_-integrin. (**A**) Schematic representation of ANGPT2-SCD and β_1_-integrin recombinant proteins used for SAXS. (**B**–**D**) SEC-SAXS of ANGPT2^19–76^–SUMO in complex with β_1_-head. The SEC elution profile (**B**); fit of ab initio using DAMMIF (green) and homology model, rigid body refined using SASREF (black), with SAXS data (blue) (**C**); and overlay of ab initio and homology models (**D**). (**E**) Schematic representation of the N-terminal truncated dimeric ANGPT2^147–496^, α_5_Calf1-2 and TIE2 (LBD, red) used for SPR and SAXS. (**F**) Binding of ANGPT2^147–496^ to α_5_Calf1-2 in SPR. (**G**–**I**) SEC-SAXS analysis of ANGPT2^147–496^ in complex with α_5_Calf1-2. The SEC elution profile (**G**), fit of ab initio (green) and homology model (black) with SAXS data (blue) (**H**), ab initio (DAMMIF) and homology models (SREFLEX) (**I**). (**J**–**L**) SEC-SAXS analysis of ANGPT2^147–496^ in complex with α_5_Calf1-2 and TIE2 LBD. The SEC elution profile (**J**), fit of ab initio using DAMMIF (green) and homology model rigid body refined using SREFLEX (black) with SAXS data (blue) (**K**), and overlay of ab initio and homology models (**L**). The χ² value is presented as a measure of the model’s goodness of fit and statistical confidence (**C**, **H**, and **K**). For homology model, ANGPT2^19–76^ (Supplemental Figure 6A), ANGPT2^147–496^ (Supplemental Figure 7A), β_1_-head (PDB ID: 7NXD), TIE2 LBD (PDB ID: 2GY7), and our crystal structure of α_5_Calf1-2 were used.

To gain insight into the topological assembly of ANGPT2 and α_5_β_1_-integrin domains, we used SAXS to generate 3D models of their complexes. We first examined whether the SCD (as a SUMOStar fusion protein) engages specific regions of the β_1_-ECD (Figure 7A). Because the SCD structure has not been reported, we computationally modelled it (using modweb, ref. 49; and hdock, ref. 50) and refined it against experimental SAXS data for ANGPT2^19–76^–SUMO using rigid body modelling (SASREF) (51), showing a good fit (χ² = 1.85) (Supplemental Figure 12, A and B). We then used SEC-SAXS to determine the complex between SCD-SUMO and the β_1_-head. SASREF modelling revealed a configuration consistent with SCD binding to the β_1_-integrin PSI domain (χ² = 1.86) (Figure 7, B–D). Analysis of the full-length β_1_-ECD using SAXS, both alone and in complex with ANGPT2^19–76^–SUMO, indicated an elongated shape (DAMMIF reconstruction; ref. 52) consistent with the extended integrin conformation. Rigid-body modeling using SASREF supported this architecture (χ² = 2.30 for β_1_-ECD and χ² = 3.30 for the complex) (Supplemental Figure 12, C–G). The molecular weights of the SCD–β_1_-integrin complexes, determined based on Porod volume as 89.5 kDa and 124.4 kDa, closely matched the theoretical values, suggesting a 2:1 complex formation (Supplemental Table 5). Because the hybrid/PSI domain is known to undergo a major conformational shift during integrin activation from bent to extended state (26), our results suggest ANGPT2 may facilitate integrin activation via binding to PSI through SCD.

### SAXS and multiprotein binding analyses reveal a 2:1 ANGPT2–α_5_-integrin complex and simultaneous TIE2 engagement.

To visualize the complex of ANGPT2 with α_5_Calf1-2 in SAXS, we used an N-terminally truncated ANGPT2^147–496^, which includes FLD and part of CCD responsible for dimerization and likely positioning of FLD domains as in full-length ANGPT2 (Figure 7E) (13). A recent report of SAXS structure of ANGPT2^147–496^ revealed it is an asymmetric dimer that binds to TIE2 in 2:1 stoichiometry (13). We found that ANGPT2^147–496^ also bound to α_5_Calf1-2 (Figure 7F). SAXS ab initio models of ANGPT2^147–496^ (generated using DAMMIF; ref. 52) showed an elongated molecule with globular structure at one end and rod-shaped structures at the other end, consistent with a prior report (13) (Supplemental Figure 13, A and B). A multi-subunit SEC-SAXS complex containing ANGPT2^147–496^ and α_5_Calf1-2 was constructed (using SREFLEX; ref. 53), based on molecular contacts identified in our co-crystal and MD simulation studies (χ^2^ =2.4) (Figure 7, G–I). The determined molecular weights of ANGPT2^147–496^ dimer and its complex with α_5_Calf1-2 were consistent with their theoretical molecular weights (Supplemental Table 5), suggesting that the dimeric ANGPT2^147–496^ binds to α_5_Calf1-2 using 2:1 stoichiometry (Figure 7I). Similar results were obtained through SAXS analysis in batch mode (Supplemental Figure 13, C and D).

Notably, the previously identified TIE2 binding site in ANGPT2 FLD (16) is accessible in our ANGPT2^147–496^–α_5_Calf1-2 SAXS model. Therefore, we tested whether ANGPT2^147–496^, α_5_Calf1-2, and TIE2 ligand-binding domain (LBD) (Figure 7E) formed a complex using SEC-SAXS (Figure 7, J–L, Supplemental Figure 8, M and N). The molecular weight of the complex was 165.6 kDa, corresponding to the estimated theoretical molecular weight of the trimeric ANGPT2^147–496^–α_5_Calf1-2–TIE2 LBD complex (177.5 kDa). Using the information on the interaction interface identified for ANGPT2 FLD and α_5_Calf1-2, and of the crystal structure of ANGPT2 FLD and TIE2 LBD (16), we generated all possible models of the 3 proteins (using SREFLEX; ref. 53) with respect to TIE2 LBD–ANGPT2 FLD interaction (Figure 7L and Supplemental Figure 13, E–H). Interestingly, the rigid body modeling presented in Figure 7L agrees with the SAXS experimental data (χ^2^ = 1.9 for model presented in Figure 7L, compared with χ^2^ = 6.2 and χ^2^ = 8.9 for models presented in Supplemental Figure 13, E and G, respectively), favoring the geometry of that model. These results indicate that the ANGPT2^147–496^ dimer can simultaneously interact with both α_5_Calf1-2 and TIE2 LBD via its asymmetric FLD domains following 2:1 stoichiometry. Supporting this, α_5_Calf1-2 immunoprecipitation pulled down TIE2 LBD only in the presence of the dimeric ANGPT2 FLD, whereas α_5_Calf1-2 and TIE2 LBD did not directly interact (Supplemental Figure 13, I and J).

To test whether α_5_β_1_-integrin functions as a coreceptor for ANGPT2/TIE2 through α_5_Calf1-mediated interaction, we generated an α_5_Calf1–Fc fusion protein and treated LECs with oligomeric ANGPT2 in its presence (Supplemental Figure 14). α_5_Calf1-Fc decreased ANGPT2-induced FOXO1 nuclear exclusion, similar to ANGPT2-FLD-Fc, which competes for TIE2 binding, yet it cannot activate the receptor, due to its dimeric configuration. These results support a role for the α_5_Calf1 in ANGPT2-induced FOXO1 signaling (Supplemental Figure 14).

### Proposed model of the ANGPT2, TIE2, and inactive α_5_β_1_-integrin complex.

**Figure 8:**
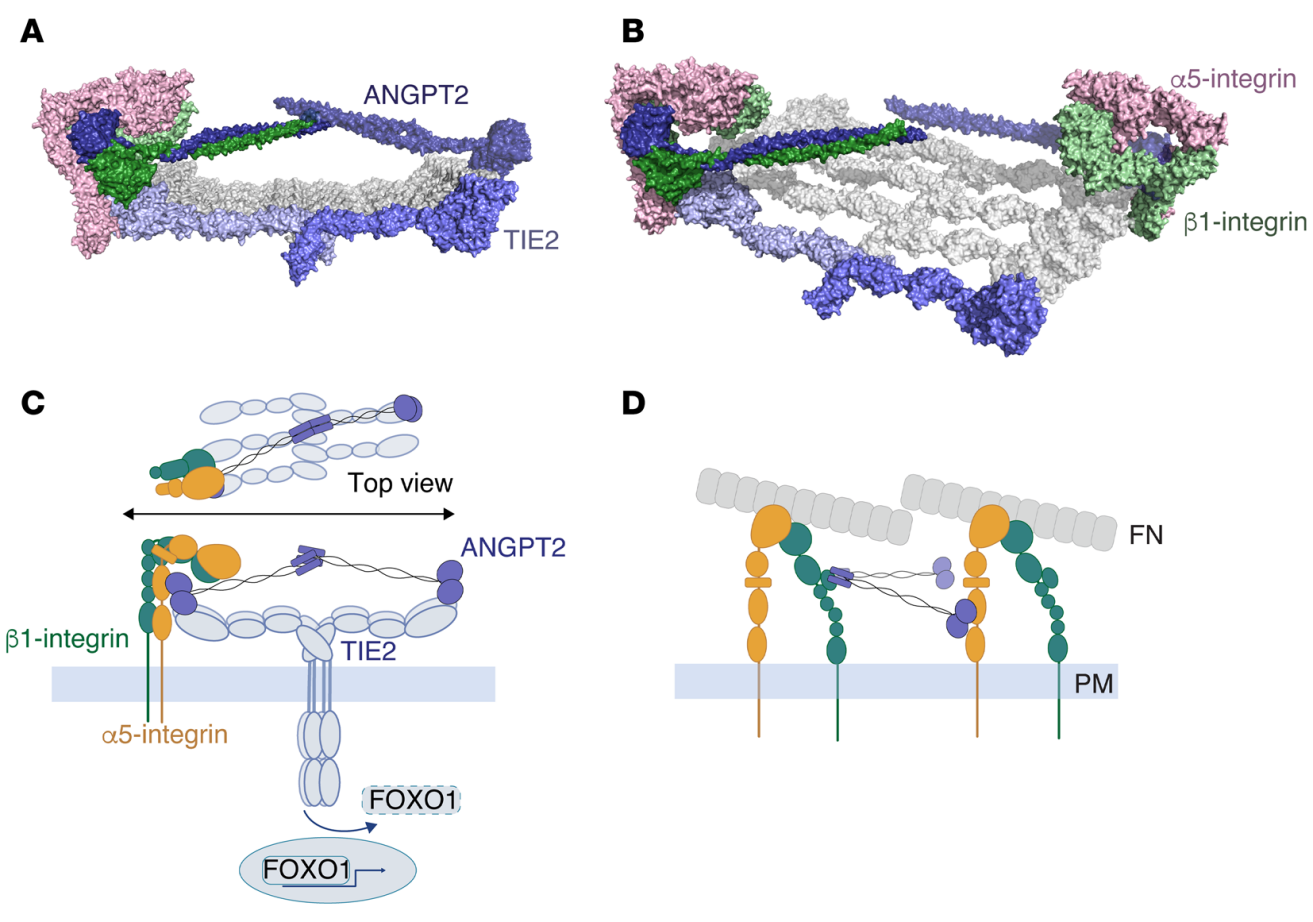
Proposed models of ANGPT2 complexes with α_5_β_1_-integrin and TIE2. (**A** and **B**) Surface model of dimeric ANGPT2^147–496^ in complex with TIE2 ECD and inactive conformation of α_5_β_1_-integrin ECD in 2 orientations with 1 (**A**) or 2 (**B**) integrin heterodimers. ANGPT2^147–496^ dimer in complex with the TIE2 LBD and Calf1-2 domains of α_5_ integrin are derived from the SAXS data in this report. The TIE2 LBD–ANGPT2-FLD interaction is consistent with the report of Barton et al. (16). An array of TIE2 ECD dimers (blue and gray) is based on interactions of fibronectin type III domain 2 (Fn2) and Fn3 of TIE2, according to Moore et al. (54) and Leppänen et al. (13, 55) and the inactive conformation of the α_5_β_1_-ECD according to Schumacher et al. (56). (**C** and **D**) Proposed mode of multimeric ANGPT2 signaling as a TIE2 agonist, in complex with inactive α_5_β_1_-integrin, leading to inactivation of FOXO1 (**C**). ANGPT2 acts as an α_5_β_1_-integrin agonist in the absence of TIE2, involving its unique N-terminal SCD interacting with β_1_-integrin PSI (**D**). This may involve clustering of integrin heterodimers. Image is not to scale.

Our results showed that ANGPT2, α_5_ integrin, and TIE2 can simultaneously interact, and that TIE2 in ECs preferentially colocalizes with inactive, but not active, β_1_-integrin. Therefore, to gain insight into the ANGPT2–α_5_β_1_-integrin–TIE2 complex, we created a model consisting of the dimeric ANGPT2^147–496^ and the ECDs of α_5_β_1_-integrin and TIE2 (Figure 8, A and B, and Supplemental Video 2). We used previously published models of the clustered TIE2-ECD homodimers and their complex with ANGPT2^147–496^ (13, 54, 55), as well as the structure for the inactive, half-bent conformation of the α_5_β_1_-ECD, obtained from a previously published, single-particle cryo–electron microscopy structure (Protein Data Bank identifier [PDB ID]: 7NXD) (56). These structures were superimposed on our SAXS complex consisting of α_5_Calf1-2, ANGPT2^147–496^, and TIE2 LBD, maintaining the interactions of ANGPT2 FLD with the TIE2 LBD (16) and with the α_5_Calf1-2 (reported in the present study). The flexible region between TIE2 FN1 and TIE2 LBD was used to rotate and adjust the orientation of the protein domains while the interfaces among the 3 proteins were maintained. Interestingly, the model of the dimeric ANGPT2^147–496^ in complex with the inactive, half-bent α_5_β_1_-integrin and TIE2 ECDs could be created without steric clashes while maintaining the overall geometry and interactions of the existing structures found in this study and previously (13, 16, 54–56) (Figure 8, A and B, and Supplemental Video 2). The model suggests, therefore, that ANGPT2 can simultaneously interact via its asymmetrically oriented FLDs with TIE2 and inactive α_5_β_1_-integrin. Taken together, our results support a model where inactive β_1_-integrin acts as a molecular scaffold in EC junctions for oligomeric ANGPT/TIE/FOXO signaling, whereas ANGPT2 acts as α_5_β_1_-integrin agonist independently of TIE2 (Figure 8, C and D).

## Discussion

Apart from their roles as cell adhesion receptors, integrins are recognized for their capacity to influence the signaling of growth factor receptors within the vascular system (2–6). While integrin engagement with EC-ECM adhesions is known to involve transitioning of integrins to an extended open conformation and reinforcing intracellular connections to the cytoskeleton, mechanistic insight into integrin signaling in the context of growth factor receptors remains limited. Here, we found that the inactive conformation of β_1_-integrin is present in EC-EC junctions, where it colocalizes with ANGPT-activated TIE RTKs. Using biophysical interaction, crystal structure, and SAXS analysis combined with MD simulations, we introduce a model of the ANGPT2/TIE2/α_5_β_1_-integrin signaling complex in which the α_5_β_1_-integrin adopts its inactive, half-bent conformation. Results using conformation-specific β_1_-integrin antibodies suggest that in this complex, inactive β_1_-integrin acts as a scaffold for ANGPT2/TIE2 signaling to regulate FOXO1 transcription factor. This function for inactive α_5_β_1_-integrin conformation is independent of ECM binding, providing a unique example of integrin noncanonical signaling in EC-EC junctions and explaining the long-standing puzzle of how integrins inhabit cell-cell junctions irrespective of matrix ligand binding (28, 29).

Recent studies have demonstrated that beyond the primary signaling mode through TIE2, ANGPTs can also signal via integrins to modulate vascular responses (13, 21–25). However, the mechanisms of ANGPT–integrin crosstalk have remained elusive, whereas ANGPT signaling via TIE2, involving the C-terminal FLD domain, has been thoroughly investigated (13, 16, 54, 55). Here, we found that the ANGPT FLD binds the α_5_Calf1 domain with high affinity and report the crystal structure of the α_5_Calf1-2 domain alone and in complex with the peptide derived from the ANGPT2 FLD (47). Guided by the co-crystal structure, which defined contacts between the ANGPT2 peptide and Calf1, we used molecular modeling and MD simulations to predict the ANGPT2 FLD–α_5_Calf1-2 interaction interface, which was validated by targeted mutagenesis. Although oligomeric ANGPT2 was required for TIE2 activation and FOXO1 nuclear exclusion in our functional studies, in our simulations we studied only the dimeric ANGPT2 FLD, because the FLD–α_5_Calf1 interaction is mediated by the C-terminal FLD independently of the N-terminal SCD that drives higher-order oligomerization.

Through our SEC-SAXS model, we discovered the topology of the binding and showed that the first FLD in the asymmetric ANGPT2 dimer (13) binds to the Calf1 domain of α_5_ integrin, and the second FLD binds TIE2. This indicates a 2:1 stoichiometry for binding of ANGPT2 to both TIE2 and α_5_ integrin in the trimeric complex. Therefore, we constructed a model of the complex comprising the ANGPT2 dimer, ECDs of TIE2, and inactive α_5_β_1_-integrin. This model was generated using the structures obtained from our study, along with prior models from Moore et al. (54) and Leppänen et al. (13, 55), as well as the cryo–electron microscopy structure of α_5_β_1_-ECD in its inactive, half-bent conformation (56). The model maintains structural integrity, avoiding steric clashes or distortions to the constituent structures, supporting the conclusion that ANGPT2 simultaneously interacts with both TIE2 and α_5_β_1_-integrin. These results support integrins as additional coreceptors for ANGPT2, alongside its classical TIE2 RTK, providing structural insights into the mechanisms of this signaling crosstalk.

We found that 3 residues in α_5_Calf1 that mediate its interaction with ANGPT2 are conserved across several α integrins. Comparative analysis of the predicted interface in the known Calf1 domain structures of αvβ3 (PDB ID: 4g1m) and αIIbβ3 (PDB ID: 3fcs) suggest that although the conserved residues are accessible, the β-propeller domain in the fully bent, inactive conformation of αvβ3 and αIIbβ3 would clash with dimeric ANGPT2^147–496^. This suggests that additional conformational changes would be required to accommodate the binding of dimeric ANGPT2^147–496^ to these integrin α-subunits. Given the structural similarity of the Calf domains, additional residues than those identified for α_5_ integrin in our study may contribute to the interactions in other integrin subunits.

Interestingly, we found that the SCD of ANGPT2 additionally interacted with β_1_-integrin. The interaction was mediated through the hybrid/PSI domain of β_1_-integrin, which is known to undergo conformational changes during the transition of α_5_β_1_-integrin from an inactive, half-bent conformation to an active extended conformation (57). Because we previously found that the ANGPT2 N-terminus, but not that of ANGPT1, activated α_5_β_1_-integrin (22), and here we further show that the ANGPT1 N-terminus does not bind β_1_-integrin, these data suggest ANGPT2 may facilitate α_5_β_1_-integrin activation by interacting with the hybrid/PSI domain in a unique manner not mediated by ANGPT1. Based on the SAXS data, ANGPT2^147–496^ adopts an elongated rod-like shape, with an estimated length of 15 nm, which is longer than the previously measured 9.5 nm distance between the upper legs of α_5_ and β_1_-integrins in the open active conformation of the α_5_β_1_-integrin heterodimer (56). Therefore, it is unlikely that ANGPT2 would simultaneously bind to α_5_ and β_1_-integrins within a single heterodimer. Alternatively, ANGPT2 may bind between 2 heterodimers, potentially clustering integrin heterodimers for increased avidity. Because ECM ligand spacing is known to regulate cell adhesion, ANGPT2 might act as a molecular ruler to spatially organize integrin heterodimers in cell adhesions (58).

The weaker TIE2 agonist activity of ANGPT2, in comparison with ANGPT1, has been attributed in part to its lower oligomeric forms (43, 59). We found that ANGPT2 oligomers were enriched in LECs and that biochemically isolated ANGPT2 oligomers activated TIE2. Moreover unlike dimers, the oligomers induced FOXO1 nuclear exclusion. Oligomeric ANGPT2 also induced a proliferative transcriptional signature in LECs, consistent with the established role of FOXO1 in suppressing EC proliferation (45). Using LECs as a model, we found that stabilizing the inactive β_1_-integrin conformation enhanced junctional TIE2 localization and nuclear exclusion of FOXO1 in an ANGPT2- and TIE2-dependent manner. Together with the colocalization of TIE2 and inactive β_1_-integrin and the inhibition of ANGPT2/FOXO1 signaling by soluble α_5_Calf1, these results support a model in which inactive α_5_β_1_-integrin can sustain junctional ANGPT2/TIE2 signaling via direct interactions, although indirect mechanisms may also contribute. Although the signaling role of inactive β_1_-integrin in this model is unexpected, because inactive integrins are classically viewed as lacking signaling capacity, they have nevertheless been identified as receptors for specific viruses, toxins, and glycan-binding galectin-3, preferentially coupling to the inactive conformer of the receptor for endocytic uptake and interacting with binding sites distal to the integrin ligand–binding site (60–62).

Our study has limitations because it primarily focuses on cell-based assays and protein–protein interactions and, therefore, does not comprehensively capture all in vivo contexts of β_1_-integrin function in ANGPT/TIE signaling. ANGPT2 signaling modes may also overlap in ECs, with ANGPT2 engaging 1 or both receptors, depending on their relative bioavailability, which is further influenced by ANGPT1 occupancy of TIE2, underscoring the context-dependent regulation of ANGPT2 signaling. In vivo, β_1_-integrin was enriched in lymphatic valves and in venous ECs together with junctional pY992–TIE2, whereas junctional TIE2 was decreased in *Itgb1^iECKO^* mice, suggesting regulation through β_1_-integrin. Although ANGPT2 is expressed both by LECs and venous ECs (20, 42), under homeostatic conditions ANGPT1 may be the primary ligand maintaining junctional pY992-TIE2 in the vena cava. We did not assess ANGPT2 oligomerization in vivo, which may further influence TIE2-mediated vascular/lymphatic remodeling or stability (14, 15). Notably, an ANGPT2 mutation associated with human lymphedema showed reduced α_5_-integrin binding, inducing lymphatic hyperplasia and dilation in mouse skin (13). Although α_9_ integrin is highly expressed in lymphatic vessels and required for lymphatic valve development (63), its structure remains unresolved; therefore, we used α_5_β_1_-integrin as a tractable model to interrogate ANGPT2/integrin/TIE2 crosstalk.

In conclusion, our results support a model in which switching between active and inactive α_5_β_1_-integrin conformations regulates the ANGPT/TIE/FOXO1 pathway, supporting ANGPT2 as a TIE2 or integrin agonist. These structural and functional analyses elucidate receptor crosstalk linking ANGPT/TIE and integrin pathways, providing a framework for future in vivo and translational studies.

## Methods

### Sex as a biological variable.

This study examined both male and female mice. The findings were similar for both sexes.

### Cell culture and reagents.

HUVECs (Cell Applications, 200P-05N; or Lonza, C2519A), human dermal BECs (HMVEC-dBl-Neo; Lonza, CC-2813), and LECs (HDLEC; PromoCell, C12216) were maintained in EBM-2 supplemented with EGM2-MV Microvascular Endothelial Cell Growth Medium Single Quots bullet kit (CC-3202, Lonza). HUVECs were cultured on 0.1% gelatin-coated culture plates and BECs and LECs on 1 μg/mL fibronectin-coated culture plates. For recombinant baculovirus production and for protein production, *Spodoptera frugiperda* (Sf9, ATCC) and *Trichoplusia ni* (Tn5, Invitrogen), respectively, insect cells were grown at +26°C in serum-free Insect-Express medium (Lonza) supplemented with 100 μg/mL penicillin-streptomycin (Sigma-Aldrich), using a shaker at 110 rpm during protein production. CHO (CHO-KI, ATCC) and HEK293FT (Thermo Fisher Scientific, R70007) cells were maintained in DMEM (Sigma-Aldrich) supplemented with 10% FBS, 4 mM glutamine and penicillin-streptomycin, and HEK293FT additionally with 4.5 g/L glucose. The following reagents were used: rhAng2 (632-AN-CF) and α_5_β_1_-ECD (3230-A5-050), both from R&D Systems. ANGPT2 peptides consisting of amino acids E358–Q366 (EFVSQLTNQ) and E352–to Q366 (EYWLGNEFVSQLTNQ) (GenScript; purity > 95%). Confluent HUVEC and LEC monolayers were starved in conditioned medium diluted 1:10 in culture medium without supplements for 3 hours, treated for 30–60 minutes with BAY826 (TIE2i; Tocris 6579; 2 μM, according to ref. 42), and stimulated from 30 minutes to 6 hours at +37°C with rhAng2 (500 ng/mL), rat AIIB2 antibody (0.1 or 0.5 μg/mL), or dimeric or oligomeric ANGPT2 (500 ng/mL) produced in CHO cells. Further information on methods can be found in the Supplemental Methods.

### Statistics.

Statistical significance was tested using GraphPad Prism 9, using a 2-tailed unpaired *t* test, or 1-way or 2-way ANOVA followed by post hoc testing using Tukey’s multiple comparison after normality testing for independent experiments. Data are presented as mean ± SEM, or as indicated in figure legends. *P* values less than 0.05 were considered statistically significant.

### Study approval.

Experimental procedures involving mice were approved by the Project Authorization Board, Regional State Administrative Agency for Southern Finland. The number of mice per group is indicated in the figures. *Itgb1^flox/flox^* (The Jackson Laboratory, 004605) and *Cdh5-CreERT2* mice [*Tg(Cdh5-cre/ERT2)Ykub*] (64) on a pure C57BL/6 background (*n* = 10 backcrosses) were housed in individually ventilated cages with enrichment materials in a specific pathogen–free facility, following Federation of European Laboratory Animal Science Associations guidelines.

### Data availability.

Crystal structures of the Calf domains of α_5_ integrin (PDB ID: 9HMH) and of the Calf domains of α_5_ integrin in complex with ANGPT2 peptide (PDB ID: 9HMI) are deposited with the Worldwide Protein Databank. RNA-Seq data from LECs stimulated with ANGPT2 oligomer and rhAng2 are deposited in the Gene Expression Omnibus (accession GSE315536 and GSE316082, respectively). Biological material is available upon request. Values for all data points in graphs are reported in the Supporting Data Values file. Uncropped Western blot images are provided in a separate supplemental file.

## Author contributions

TS, SKP, and AVM conceived the study, designed and conducted experiments, and analyzed and interpreted results. AP and SKB designed, conducted, and analyzed experiments using ECs and mouse tissues. KL analyzed RNA-Seq data. KG and QL performed mouse experiments; MK conducted the cell experiments. GE and IV designed the simulations, and GE performed and analyzed the molecular modeling and dynamics studies. TK participated in SEC-MALS. MP provided reagents. JI edited the manuscript and designed experiments. VML designed experiments, provided reagents, and interpreted the results. IV and PS supervised the research. PS conceived the study, designed experiments, and analyzed and interpreted the results. All authors wrote the manuscript. TS, SKP, AP, and AVM mutually agreed on the order of authors based on their contribution to experiments. Priority application: FI20253939 (PS, SKP, AP, AVM, TS, and SKB).

## Conflict of interest

The authors have declared that no conflict of interest exists.

## Funding support

- The Wihuri Foundation (to PS and VML).
- European Research Council under the EU Horizon 2020 research and innovation program (grant agreement 773076 to PS).
- Sigrid Jusélius Foundation (to PS and IV).
- Cancer Foundation Finland (to PS).
- Research Council of Finland Centre of Excellence program (346131 [to JI], 346134
- [to PS], 346135 and 364185 [to IV]).
- Research Council of Finland (grants 310075 [to PS], 347771 [to SKP], and 331349 and 336234 [to IV]).
- Research Council of Finland’s Flagship InFLAMES (337530 and 357910 to JI).
- Helsinki Institute of Life Science Fellow Program (PS and IV).
- Human Frontier Science Program (grant RGP0059/2019 to IV).
- Deutsche Forschungsgemeinschaft (SFB 1531 – 456687919 to MP).
- German Center for Cardiovascular Research (DZHK, partner site Berlin to MP).
- Open access funded by Helsinki University Library.

## Supplementary Material

Supplemental data

Unedited blot and gel images

Supplemental video 1

Supplemental video 2

Supporting data values

## Version 1 06/15/2026

Electronic publication
