# Supplementary material for: Inactive β1-integrin acts as a junctional scaffold for angiopoietin/TIE2/FOXO1 signaling
Source: J Clin Invest. 2026 Jun 15;136(12):e190552. doi: 10.1172/JCI190552 (PMC13262729; doi:10.1172/JCI190552)
Supplement: Supplemental data [file jci-136-190552-s309.pdf]

Supplemental Material for

Inactive β1-integrin acts as a junctional scaffold for Angiopoietin / TIE2 / FOXO1 signaling

Tuomas Sipilä[1^], Srinivas Kumar Ponna [1^], Abhinandan Venkatesha Murthy [1]#, Anne Pink #[1], Giray Enkavi[2], Shraman Kumar Bohra[1], Klaudia Lewna[1], Keerthana Ganesh[1], Qina Liu[1], Mirka Korhonen[1], Tommi Kajander[3], Michael Potente[4], Johanna Ivaska[5,6], Ilpo Vattulainen [2], Veli-Matti Leppänen[5, 7] and Pipsa Saharinen[1,7,8*]

Supplemental Methods

Cell stimulations

Confluent HUVEC and LEC monolayers were starved in conditioned media diluted 1:10 in culture media without supplements for 3 h, treated for 30-60 min with BAY826 (TIE2i; Tocris 6579, 2 μM (1)), LY294002 (PI3Ki; Cell signalling 9901, 10 μM) or Fc fusion proteins (α5Calf1-Fc, ANGPT2-FLD-Fc or control Fc, all 10 μgml^{-1}) and stimulated for 30 min - 6 hours at +37°C with rhAng2 (500 $ngml^{-1}$), Comp-Ang1 (200 $ngml^{-1}$) (2), rat AIIB2 antibody (0.1 or 0.5 μgml^{-1}) or dimeric or oligomeric ANGPT2 (500 $ngml^{-1}$) produced in CHO cells.

Immunofluorescence staining of cells

ECs on coverslips were fixed in 4% PFA for 10 min at RT, washed with PBS, permeabilized for 5 min with 0.1% Triton X-100 in PBS, blocked in 1% BSA-PBS for 1 h, and stained using primary antibodies for 60 min at RT, washed with PBS, stained with secondary fluorescently conjugated antibodies (Life Technologies) for 30 min, and mounted in DAPI mounting medium (Abcam, ab104139). For cell surface staining on ice, HUVECs on coverslips were blocked with 1% BSA-EBM-2 for 10 min, incubated with primary antibodies for 30 min, washed with cold DPBS and fixed in 4% PFA-PBS for 20 min. The following antibodies were used: active β1-integrin (mAb12G10, Abcam, ab30394), inactive β1-integrin (mAb13, 552828, BD), TIE2 (AF313, R&D), Ang2 (AF623,

R&D), FOXO1 (Cell Signaling, 2880S), VE-cadherin (CDH5, D87F2, Cell Signaling, 2500S), CD144 (CDH5, Pharmingen, 555661).

Proximity ligation assay

Proximity ligation assay was performed using the Duolink® Fluorescence protocol for the in Situ Detection Reagent Red kit (Sigma-Aldrich, Duo92008). Donkey anti-rat secondary antibody (Invitrogen, A18747) was labeled using Duolink® In situ Probemaker Plus kit (DUO92009). The cells on coverslips were starved for 2 h in starvation media and stimulated with rhAng2 or HBS buffer, as a control, for 30 min. The coverslips were fixed with 4% PFA for 10 min at RT, permeabilized using 0.1% Triton X-100 in PBS for 5 min, and probed using mAb13 (1.6 μgml^{-1}, BD, 552828, rat anti-human) or 9EG7 (1.6 μgml^{-1}, BD, 553715, rat anti-human) together with polyclonal goat anti-Tie2 antibody (40 $ngml^{-1}$ R&D Systems, AF313) or goat anti-Ang2 antibody (1.1 μgml^{-1} R&D Systems, AF623) for 1 h RT. As an isotype control rat IgGa2 k antibody (1.6 μgml^{-1} Biolegend, 1038080) was used. Secondary antibodies were anti-goat minus (1:5 diluted DUO92006) and anti-rat plus (1.2 μgml^{-1}).

Analysis of ANGPT2 in cell culture media

Conditioned media was collected from HUVECs and LECs cultured for 48 h, concentrated 10x using Amicon ultra 10k MWCO centrifugal filters (Millipore) and equal volumes of media were analyzed under reducing and non-reducing conditions using Novex WedgeWell 8% TRIS-Glycine gel

(Invitrogen) and ANGPT2 was analyzed using immunoblotting and quantified-using AzureSpot Pro software (Azure Biosystems). Oligomer to dimer ratio was calculated by dividing the background-substracted peak areas of dimer and oligomer bands.

Subcellular fractionation and phosphoprotein analysis

For analysis of phosphoproteins, LECs were starved overnight in culture media without supplements, treated with BAY826 (30 min, 2 μM according to (1)), stimulated with oligomeric ANGPT2 (30 min, 500 $ngml^{-1}$) and lysed in IP buffer (50 mM Tris pH 7.4, 150 mM NaCl, 1% Igepal CA-630, 1 mM EGTA, 10% glycerol, 2 mM $MgCl_2$) supplemented with protease and phosphatase inhibitors (COMPLETE mini: 04693159001, PhosSTOP: 4906845001, Roche, 2 mM Na_3VO_4). Mouse lungs were lysed in RIPA lysis buffer (50 mM Tris-HCl pH7.4, 1% Igepal CA-630, 150 mM NaCl, 1 mM EDTA, 0.25% Na-DOC) supplemented with protease and phosphatase inhibitors. TIE2 was immunoprecipitated from LEC lysates using anti-human TIE2 (AF313, R&D) or from mouse lung lysates using anti-mouse TIE2 (AF762, R&D) and captured using G Dynabeads (10004D, Thermo Scientific). Nuclear and cytoplasmic fractions were isolated using NE-PER™ reagents (Thermo Fisher, 78833) from LECs on culture plates starved for 3 h and stimulated for 30 min with rhAng2, rat AIIB2 antibody or oligomeric ANGPT2.

Immunofluorescence staining of mouse tissues

Itgb1$^{flox/flox}$ (Jackson Laboratory, stock #004605) and Cdh5-CreERT2 mice (Tg(Cdh5-cre/ERT2)Ykub) (3) on a pure C57BL/6 background were housed in individually ventilated cages with enrichment materials in a specific pathogen-free facility, following FELASA guidelines. Experimental procedures were approved by the Project Authorization Board, Regional State Administrative Agency for Southern Finland. Both sexes were used. The number of mice per group is indicated in the figures. Gene deletions were induced in 8–16-week-old mice using subcutaneous administration of tamoxifen for 5 consecutive days (Sigma T5648, 2 mg/mouse/day in corn oil, Sigma C8267) and analyzed 7 days after the last dose. Tamoxifen-treated Itgb1$^{flox/flox}$;Cdh5-CreERT2 mice (*Itgb1*^{iECKO}) were compared to similarly treated controls. LPS was administered in wild-type C57BL/6 mice (O55:B5, Sigma L2880, 11 mg/kg intraperitoneally) for 16 h. Ears and vena cava were collected for immunostaining and immediately fixed (ears: 4% PFA in PBS for 1 h; vena cava: 1% PFA in PBS for 30 min). Ventral ear skin was separated from the dorsal side for staining. Tissues were blocked in donkey immunomix (DIM: 5% donkey serum, 1% BSA, 0.25% Triton X-100 in Dulbecco's PBS) supplemented with PhosSTOP (Sigma, 4906837001), incubated with primary antibodies in DIM for 24 h at 4°C, washed with 0.3% Triton X-100 in PBS, incubated with fluorescently labeled secondary antibodies overnight at room temperature and washed with 0.3% Triton X-100 in PBS. Whole mounts were post-fixed in 4% PFA in PBS for 10 min, rinsed in PBS, and mounted in Vectashield with DAPI (H-1200, Vector Labs). Primary antibodies included rat anti-

mouse VE-cadherin (BD 555289; eBioscience 14-1441), rabbit anti-mouse Lyve1 (Millipore AB2988), rabbit anti-mouse/human pY992-TIE2 (R&D AF2720), and armenian hamster anti-mouse inactive integrin β1 (HMb1, BioLegend 102210). Secondary antibodies were AF488-, AF568-, AF594-, and AF647-conjugated (Thermo Scientific).

Lentiviral vector delivery in cells

For production of lentiviral vectors HEK293FT cells were transiently transfected with packaging plasmids pCMVg and pCMVdelta8.9, and the following previously validated shRNA clones from TRC1 library: *TIE2*: GCTTCTATACAAACCCGTTAA (TRCN0000000415), ITGB1: GCCTTGCATTACTGCTGATAT (TRCN0000029645), *ANGPT2:* GATGATAGAAATAGGGACAAA (TRCN0000059227) (4) or doxycycline-inducible lentiviral expression of a constitutively nuclear human FOXO1 (FOXO1^{A3}) (5). LECs were transfected with shRNA lentiviral vectors, filtered through a 0.45-μm filter in the presence of 0.1% Polybrene (Sigma-Aldrich) for 5 h - o/n, and subsequently cultured in new growth media for 48 h. FOXO1 or empty vector transfected LECs were selected with EBM containing 1 μg ml^{-1} puromycin (Sigma, P8833) and induced using doxycycline (Sigma, D5207) for 48-72 hours before analysis.

Immunoblotting

For immunoblotting, equal amounts of protein determined by the BCA assay were separated on Novex WedgeWell 8% or 4–12% Tris-Glycine gels (Invitrogen) and transferred to PVDF membrane

(Merck Millipore). The membranes were blocked in 5% BSA or milk in TBST (TBS - 0.1%Tween-20), incubated with primary antibodies overnight at 4°C, washed with TBST, and probed with HRP conjugated secondary antibodies (1:4000, Dako) in 5% BSA-TBST for 60 min at RT. Chemiluminescence was detected using SuperSignal West Pico Plus or Femto Maximum Luminol reagent (Thermo Scientific, 34577, 34096) and Azure 500 imaging system (Azure Biosystems), and quantified using Fiji ImageJ (v1.52n, NIH). Normalization was performed as follows: phospho-AKT to total AKT and GAPDH; phospho-TIE2 to immunoprecipitated TIE2. To calculate the nuclear-to-cytoplasmic FOXO1 ratio, FOXO1 in nuclear extracts was normalized to lamin A/C, FOXO1 in cytoplasmic extracts was normalized to α-tubulin, and the ratio was obtained by dividing the normalized nuclear value by the normalized cytoplasmic value. Primary antibodies used: goat anti-human TIE2 (AF313, R&D), goat anti-mouse TIE2 (AF762, R&D), mouse anti-phosphotyrosine 4G10 (Millipore, 05-321), rabbit anti-FOXO1 (Cell Signaling, 2880), rabbit anti-lamin A/C (Novus, NBP2-19324), mouse anti-α-tubulin (Santa Cruz, sc-32293), rabbit anti-phospho-AKT(Ser473) (Cell Signaling, 9271), mouse anti-AKT (Cell Signaling, 9272), and rabbit anti-GAPDH (Cell Signaling, 97166). Secondary antibodies were swine anti-rabbit (P0217) and rabbit anti-goat (P0499) from Dako, and goat anti-mouse (115-035-003) from Jackson ImmunoResearch.

RT-qPCR analysis

RNA isolation was performed using RNeasy Micro Kit (Qiagen, 74004) or Nucleospin RNA isolation kit (Macherey-Nagel, 740955.50) and cDNA was synthesized using High Capacity cDNA Reverse

Transcription Kit (Thermo Fisher Scientific, 4368813). Real-time qPCR was performed with DyNAmo HS SYBR Green qPCR kit (Thermo Scientific, F410-XL) in a BIO-RAD C1000 Thermal cycler (Bio-Rad). The data were analyzed using the ddCt method and GAPDH as a housekeeping gene. Primers used: *GAPDH* (TGCACCACCAACTGCTTAGC, GGCATGGACTGTGGTCATGAG), *ANGPT2* (CAGATTTTGGACCAGACCAGTGA, TCAATGATGGAATTTTGCTTGGA).

Bulk RNA-Seq

RNA-seq libraries were sequenced on an Illumina NextSeq 500 platform to an average depth of 65 million paired-end reads per sample (n = 4 biological replicates per condition; read length, 2×75 bp). Reads were quality-checked with FastQC; no adapter or quality trimming was performed. Reads were aligned to the human reference genome (GRCh38) using STAR v2.7.6a with gene annotation from GENCODE v49 (GTF) and summarized to gene-level counts with featureCounts v2.0.1 (paired-end, reverse-stranded, counting reads overlapping exons per gene). Differential expression was analyzed in R using DESeq2 v1.44.0 (6), with the design formula ~ condition after filtering to retain genes with ≥10 counts in at least 4 samples. Gene set enrichment analysis (GSEA) was performed using the fgsea package on Hallmark and Gene Ontology (GO) Biological Process gene sets; genes were ranked by DESeq2 $\log_2$ fold change, and enriched terms with Bonferroni-adjusted $P < 0.05$ were considered significant. Variance-stabilizing transformation (VST)–transformed expression was used to infer transcription factor activity with DoRothEA regulons A–E and VIPER (7), yielding relative

regulatory activity scores per sample. VIPER scores were compared across conditions by one-way ANOVA with Tukey's multiple-comparisons test, followed by Benjamini–Hochberg FDR correction across TFs.

Protein production in insect cells

Recombinant proteins were produced using the Baculovirus expression vector pFastBac1 (Invitrogen), with a mellitin signal peptide and a C-terminal Fc-tag, cleavable with Factor Xa protease, or twin-streptag and an N-terminal hexahistidine (His)-tag (Supplemental Table 1). Recombinant baculoviruses, produced in Sf9 cells, were harvested 72 h post-transfection by centrifugation for 5 min at 4000 x g. For protein expression, Tn5 cells were transfected with recombinant baculoviruses at high multiplicity and at three days post infection, the supernatant was harvested by centrifugation for 20 min at 8000 x g. Fc fusion proteins were purified from the insect cell supernatants using Protein A sepharose column (4CLB, GE Healthcare). After extensive washing with HEPES buffered saline (HBS, 20 mM HEPES, pH7.5, 150 mM NaCl), the Fc fusion proteins were eluted using 0.1 M Glycine, pH 3.0. His-tagged proteins were purified from supernatants supplemented with 2 mM Imidazole and 1 mM $NiCl_2$ using Ni^{2+}-charged resin columns (Ni sepharose 6 Fast Flow; GE Healthcare) under gravity flow or using a peristaltic pump. After extensive washing with the washing buffer 20 mM imidazole in HBS, the proteins were eluted with 0.4 M imidazole in washing buffer. Proteins containing both His-tag and twin-strep-tags were further loaded onto a strep-tactin®XT Superflow® high-capacity resin, washed using HBS and eluted using 50 mM Biotin in

HBS. All eluates were further purified by size exclusion chromatography using a Superdex 200 increase 10/300 (GE Healthcare) column in HBS and concentrated to desired concentration by centrifugal filter units (Amicon Ultra-15 MWCO 10 kDa or 30kDa, Millipore).

Production and purification of ANGPT2 in CHO cells

Human full-length *ANGPT2* cDNA was cloned into the pcDNA mammalian cell expression vector with a C-terminal Strep-tag and transfected using Fugene HD (Promega, E2311) into CHO cells, selected using 150 μgml^{-1} zeocin. ANGPT2 was purified from cell supernatant using streptactin sepharose affinity resin (IBA) and eluted with 50 mM biotin (Sigma-Aldrich) in HBS. The eluted protein was concentrated using a 100 kDa MWCO centrifugal filter (Millipore) and the different oligomeric forms of ANGPT2 were separated using size exclusion chromatography using Superose6 3.2/300 GL column.

Surface plasmon resonance

SPR experiments were performed using Biacore™ T100 system (GE Healthcare). Proteins were immobilized on chips using amine coupling chemistry according to the Biacore™ T100 system protocol (Supplemental Table 5). Samples were diluted and experiments performed using HBS-P (Cytiva, BR100671) or HBS-P supplemented with 2 mM $MgCl_2$ and 2 mM $MnCl_2$ (Sigma Aldrich) and flow rate 30 μls^{-1}. Kinetic reactions were performed at +25 °C. Chips were regenerated using 2 M $MgCl_2$, which was injected for 30 s. For ANGPT injections, Biacore Series S Sensor Chip C1

(Biacore GE Healthcare 29104944) were used. For immobilization of ANGPTs, Series S Sensor Chip CM3 or CM5 (Biacore GE Healthcare 29104990) were used. Kinetic modelling and data handling was performed using Biacore Bioevaluation 0.1 (GE Healthcare) software, using two-state reaction kinetics (8) due to the dimeric structure of ANGPT-Fc molecules, multiple binding sites and flexible nature of the integrin ectodomains.

Microscale thermophoresis

For MST, proteins were labeled using second-generation red amine-reactive NT-650-NHS fluorescent dye and the Nanotemper labeling kit (MO-L011), using 1:3 protein:dye concentration ratio. Excess dye was removed by centrifugation using Amicon ultra 10k MWCO centrifugal filters (Millipore). Protein concentration was determined using NanoDrop Microvolume Spectrophotometer after correcting for the extinction coefficient of the proteins. Binding experiments were carried out in 10 mM HEPES, pH 7.4, 150 mM NaCl, 0.05% Tween-20, using a fixed concentration (5 nM) of the fluorescently labeled proteins (target) and two-fold serially diluted decreasing concentrations of the unlabeled proteins or peptides (ligand). 16 serial dilutions of the ligand and labelled protein were loaded into the capillaries (Nanotemper, MO-K022) and analyzed using the Monolith NT.115 instrument. All experiments were carried out at +25 °C using a medium MST power and 20% LED power in triplicates for each experimental setup.

Protein crystallization and structure determination

α5Calf1-2 was crystallized in a space group $P2_12_12_1$, and the crystal structure was determined at 1.92-Å resolution using molecular replacement (Supplemental Table 2). For crystallization, α5Calf1-2 was concentrated to 3.5 mg/mL in HBS. Crystallization conditions were screened using the sitting-drop vapor diffusion technique. Apo α5Calf1-2 crystals grew in a few days at room temperature over a reservoir solution of 0.1M Bistris pH 5.5, 0.2M MgCl2, 25% PEG 3350 (wt/vol). α5Calf1-2 in complex with a the ANGPT2 ligand peptide (E358 to Q366) was crystallized also in a space group $P2_12_12_1$. Crystals were grown in 0.2M ammonium formate; 20% polyethylene glycol 3350 (wt/vol). Both crystals contained one monomer of α5Calf1-2 in the asymmetric unit. For data collection at 100 K, the crystals were frozen in liquid nitrogen in the reservoir solution supplemented with 5% glycerol for the unliganded α5Calf1-2 crystals and no cryo protectant for the peptide complex crystals. Complete datasets with 1.9 Å and 1.6 Å resolution were collected from native crystals of the unliganded and peptide complexed α5Calf1-2, respectively, at the European Synchrotron Radiation Facility (ESRF) beamlines in Grenoble, France (Supplemental Table 2). Data was processed with XDS (9) and the CCP4 (10) suite of programs. The unliganded structure was solved by molecular replacement using the α_V-integrin Calf1-2 domains as the model (PDB:ID-1L5G). Automated model building in PHENIX (11) was used to generate an initial model and improve the phases. The model was completed by iterative refinement in Refmac and model building in Coot (12). A subset of 5% of the diffraction data was omitted from refinement for calculating the free R factor (Rfree). The

α5Calf1 domain is an all-β structure with 10 β-strands separated by 10 loops, whereas the α5Calf2 domain consists of 12 β-strands separated by 8 loops and 2 short α-helices. Further, all cysteine residues in α5Calf1-2 formed disulfide bonds. The crystal structure of the α5Calf1-2 in complex with the ANGPT2 peptide was solved by molecular replacement using the unliganded structure of α5Calf1-2 and omitting the peptide in the model. The electron density for the peptide is clearly visible in the omit map, confirming the co-crystal structure. Modeling of the peptide in this structure revealed the side chains for 8 residues, with three residues forming interactions with Calf1. As, the side chain of the first residue (P1) in the peptide was not visible, it was replaced by alanine in the model submitted to PDB. The final model contains residues 543– 568 in chain A, N-linked glucosamine moieties in Asn560 and 45 solvent molecules. All molecular graphics figures were generated with PyMOL (W. Delano; www. pymol.org/).

SAXS solution structure determination of the TIE2 LBD, ANGPT2$^{147\text{-}496}$ and α5-integrin (Calf1-2) complexes.

Dimeric ANGPT2$^{147\text{-}496}$, α5Calf1-2 and an equimolar complex of ANGPT2$^{147\text{-}496}$ and α5Calf1-2 at concentrations 24, 29, 12 μM respectively, were used for the SAXS data collection in batch mode at ESRF (Grenoble, France). Data were processed using the ATSAS software PRIMUS (13) and the Scatter software (14), processed output files were used to generate the *ab initio* models with the ATSAS package programs. Using SASREF, rigid body models were generated, and CRYSOL was used to fit the models with the scattering intensities. The radius of gyration (R_g) and the maximum

dimensions (Dmax) were calculated from the scattering data using the AUTOGNOM program. SEC-SAXS data for both ANGPT2$^{147\text{-}496}$ - α5Calf1-2 complex and ANGPT2$^{147\text{-}496}$ - α5Calf1-2 --TIE2-LDB were collected on the ESRF beamline BM29 using BioSEC 300 4.6x300 in Shimatzu HPLC system with an auto-sampler. The buffer-subtracted, averaged scattering data were analyzed using the ATSAS software package. For rigid body modelling of the complexes, the crystal structure of the Calf1-2 from this study, the TIE-LBD crystal structure (PDB:2GY7) and the previous ANGPT2$^{147\text{-}496}$-FLD SAXS model (15) were used. Rigid body models were refined and the Ab initio 3D bead model reconstructions were generated from the scattering data using SREFLEX and DAMMIF in ATSAS. Multiple independent models were calculated and averaged to obtain a representative final model.

ANGPT2$^{19\text{-}76}$ - SUMO-β1-integrin complexes. The SAXS structure of ANGPT2$^{19\text{-}76}$-SUMO was obtained in batch mode at Diamond Light Source (DLS), Oxford, UK using B21 BioSAXS beamline. Since the structure of the SCD is not known, we used computational tools to generate a model of ANGPT2$^{19\text{-}76}$-SUMO monomer using modweb. Subsequently, since ANGPT2$^{19\text{-}76}$-SUMO was purified as a dimer we modelled dimeric ANGPT2$^{19\text{-}76}$-SUMO, using hdock (16), and used this dimeric structure for modeling using SASREF, against the experimental SAXS data.

Since we found that the ANGPT2 N-terminus bound to the β1-integrin PSI domain, we collected SEC-SAXS data of ANGPT2$^{19\text{-}76}$-SUMO complexes with β1-ECD and β1-head, using BioSEC 300 4.6x300 at ESRF Grenoble. To model the complexes, we used the model of ANGPT2$^{19\text{-}76}$-SUMO

together with a 3D solution structure of β1-ECD in an extended conformation or β1-integrin head, based on β1-integrin cryoEM structure (PDB id: 7NXD) using SASREF rigid body modelling.

Pulldown assay

Strep-tagged Integrin α5–CALF12 (10 µg) was incubated with 30 µL of Dynabeads MyOne Streptavidin T1 (Invitrogen, 65601) for 30 min at 4 °C, followed by incubation with Angpt2–FLD–Fc and TIE2-LBD–His for 1 h at 4 °C with gentle mixing. Beads were then washed six times with 100 µL HBS buffer to remove unbound proteins. Bound protein complexes were eluted by adding 30 µL of 1× Laemmli buffer supplemented with 10% β-mercaptoethanol in HBS, and samples were boiled at 95 °C for 7 min prior to analysis by Western blotting.

Multi-angle laser light scattering

SEC-MALS measurements were run in a BioSEC 300 4.6x300 in HBS with an Agilent HPLC with UV and fluorescence detectors, Wyatt Technology miniDAWN TREOS and Optilab REX detectors. Samples were loaded at about 1mg/ml concentrations, and all experiments were performed at room temperature (25 °C). Data collection and SEC-MALS analysis were performed with ASTRA 6.1 software (Wyatt Technology). All the samples were run with 50mM Hepes, 150 mM NaCl buffer. Data was analyzed with ASTRA 6 software (Wyatt Technology Corporation).

Image analysis

Images were acquired using Leica Stellaris 8 Falcon confocal microscope (HC PL APO 63x /NA 1.40 oil objective) or Leica Stellaris 5 confocal microscope equipped with a 63× glycerol immersion objective. All confocal images represent maximum intensity projections of Z-stacks unless indicated otherwise and were analysed using Fiji ImageJ software (version 1.52n, NIH). PLA signal was quantified using binary masks of PLA channel and Analyze Particles command in Fiji ImageJ from 3 - 4 20x images (n = 200-350 cells per experiment) and normalized to the total cell number (nuclei), from 3 independent experiments. For FOXO1 subcellular localization analysis DAPI binary mask was generated and applied to the FOXO1 channel and the integrated density of FOXO1 signal under the DAPI mask was measured (nuclear FOXO1). FOXO1 staining outside the DAPI mask was considered cytoplasmic FOXO1. The ratio of nuclear vs. cytoplasmic FOXO1 was calculated and normalized to the total cell number (nuclei). In HUVEC, FOXO1 was quantified from 3 - 4 20x images per coverslip, and in LEC, from 5 63x images. For junctional HMb1, phospho-TIE2, mAb13 and TIE2 quantification, binary junctional mask was created based on CDH5 staining and the integrated density under junctional mask was measured and normalized to the total cell number (nuclei) or CDH5 area. Colocalization was measured using Mander's overlap coefficient (MOC) (17) and was quantified from Z-stack images using ImageJ and BIOP JaCOP plugin (18). Total TIE2, phospho-TIE2 and CDH5 were quantified using thresholding and the integrated density was normalized to the total cell number (nuclei) or CDH5 area. Quantification was performed from 2-5

63x images per coverslip. Experiments were independently repeated minimum of three times, as indicated in Figure legends.

Modelling of the dimeric ANGPT2-FLD - α5-integrin Calf1-2 complex

To model the interaction between ANGPT2 and α5-integrin at the minimal functional interface, we focused on the dimeric ANGPT2 FLD, which is sufficient and responsible for binding to α5Calf1-2. We used a custom local docking approach to dock dimeric ANGPT2 FLD structure (15) on to α5Calf1-2, based on knowledge of the interaction of the ANGPT2 peptide ligand with the α5-integrin Calf1-2 domain in our co-crystal structure. We started by manually docking the E358-Q366 helical segment of the FLD, which corresponds to the peptide ligand, into the corresponding position in the co-complex of ANGPT2-FLD and α5Calf1-2. To explore systematically other ANGPT2-FLD and α5Calf1-2 interfaces, we applied incremental rigid body rotations of the major principal axis of the E358-Q366 helical segment in 1° intervals in clockwise and counterclockwise directions to create 100 distinctly oriented structures of the two proteins. Each of these models maintains the position of the helical peptide segment with respect to the experimental structure. All 100 structures were then subjected to the constrained relax protocol of the Rosetta Software Suite to resolve clashes. The relaxed complex structures were rescored using the Rosetta's InterfaceAnalyzer (19). The complex with the best packstat score without any knots was further refined using Phenix's model_idealization tool (11) and used for saturation mutagenesis analysis and MD simulations.

Construction of the atom-scale molecular dynamics simulation systems

All simulation systems were prepared based on the structure of the Calf1-2 domains of α5-integrin (this study) and/or the dimeric structure of the FLD domain of ANGPT2 (15). The dimeric ANGPT2-FLD structure was used, as it mediates binding to α5-Calf1 independently of the N-terminal domain responsible for higher-order oligomerization. All mutations were made using MODELLER 10.2 (20). Protonation states of the residues were assigned at pH 7 using the PDB2PQR webserver (21). The protein was placed in a dodecahedron simulation box whose size was chosen to maintain a minimum distance of about 1.5 nm to the edges. Each system was solvated and neutralized with 0.15 M KCl solution. The topologies for each molecule were initially prepared with the LeAP program distributed with AmberTools20 (22). Using the ParmED tool distributed with AmberTools20, Hydrogen Mass Repartitioning (HMR)(23) was applied to the protein, and all topologies were converted to the GROMACS format.

Simulation protocols

All atom-scale MD simulations were carried out using GROMACS 2020 (24) employing the Amber ff19SB (25) force field for the proteins, the OPC model for water (26), and a complementary ion parameter set (27). A leap-frog algorithm was used to integrate the equations of motion. All bonds were constrained using the LINCS algorithm (28). Periodic boundary conditions were used in all three dimensions. A short-range interaction cutoff of 1.0 nm was used for both electrostatic and van der Waals interactions. The long-range electrostatic interactions were calculated using the smooth

particle mesh Ewald method (29) with a Fourier spacing of 0.12 nm and a fourth-order interpolation. Van der Waals interactions were treated with the Lennard–Jones potential, and long-range dispersion corrections were applied to both energy and pressure.

Prior to production runs, each system was subjected to steepest descent minimization and successive 100 ps and 1 ns long equilibration simulations in the NVT and NpT ensembles. In the equilibration simulations, a 2 fs time step, and the Berendsen thermostat and barostat (30) were employed. Harmonic position restraints with a force constant of 1000 kJ/mol/nm^2 were applied to the heavy atoms of the protein. The production simulations of the ANGPT2-FLD and α5Calf1-2 co-complex were 500 ns - 1 μs long, repeated 3 times. The Calf1-2 mutants were simulated for 500 ns.

All production runs were performed in the NpT ensemble using an integration time step of 4 fs by applying HMR (23) to the protein. The protein and the solvent (water and 0.15 M KCl) were coupled to separate temperature baths at 310 K using the Nosé-Hoover thermostat (31, 32) with a time constant of 1.0 ps. Pressure was maintained isotropically at 1 atm using the Parrinello–Rahman barostat (33) with a time constant of 5 ps and a compressibility of 4.5×10^{-5} bar^{-1}.

Saturation mutagenesis analysis

To predict the effect of all possible amino acid substitutions of the following Calf1 residues: R694, N724, R755, Q762, D764, Q766 on the energy of binding to ANGPT2-FLD dimer, we performed single site saturation mutagenesis using Flex ddG (34). For experimental mutagenesis, amino acid

substitutions predicted to reduce the binding affinity, without distorting the overall structure were selected, and confirmed using MD simulations and CD.

Circular Dichroism

Circular dichroism (CD) spectroscopy was performed using a Chirascan CD spectrometer (Applied Photophysics, Leatherhead, UK). CD data were collected between 280 and 190 nm at +20°C using a 0.1 cm path-length quartz cuvette. CD measurements were acquired every 1 nm with 0.5 s as an integration time and repeated three times with baseline correction. Data were processed using Chirascan Pro-Data Viewer (Applied Photophysics), and CDNN (1), or BeStSel (2). The direct CD measurements (θ; mdeg) were converted into mean residue molar ellipticity ([θ]MR) by Pro-Data Viewer.

Supplemental Movie Legends

Supplemental Movie 1. Molecular modeling of the interaction between dimeric ANGPT2 FLD and Calf1-2 of α5-integrin. Dimeric ANGPT2 FLD structure ($ANGPT2^{147\text{-}496}$) (15) was docked on to α5Calf1-2 structure determined in this study, using a previously published docking approach (35). In short, we first docked ANGPT2-FLD onto α5Calf1-2 based on the peptide position. We then applied rigid body rotations to ANGPT2-FLD around the helical segment that corresponds to the peptide (E358-Q366). 100 possible ANGPT2-FLD-α5Calf1-2 interfaces were explored while maintaining the peptide–α5Calf1-2 interface (see Supplemental Methods for details), allowing systematic analysis of ANGPT2-FLD - α5Calf1-2 interfaces compatible with our experimental crystal structure.

Supplemental Movie 2. Surface model of dimeric $ANGPT2^{147\text{-}496}$ in complex with TIE2 ECD and inactive conformation of α5β1-integrin ECD. $ANGPT2^{147\text{-}496}$ dimer in complex with the TIE2 LBD and Calf1-2 domains of α5-integrin is derived from the SAXS data in this manuscript. The TIE2 LBD-ANGPT2-FLD interaction is consistent with Barton et al. (36). An array of TIE2 ECD dimers is based on interactions of fibronectin type III domain 2 (Fn2) and Fn3 of TIE2, according to Moore et al. and Leppänen et al. (15, 37, 38) and the inactive conformation of the α5β1-ectodomain according to (39).

Supplemental References

1. Kraft M, et al. Angiopoietin-TIE2 feedforward circuit promotes PIK3CA-driven venous malformations. *Nat Cardiovasc Res.* 2025;4(7):801-20.
2. Cho CH, et al. COMP-Ang1: a designed angiopoietin-1 variant with nonleaky angiogenic activity. *Proc Natl Acad Sci U S A.* 2004;101(15):5547-52.
3. Okabe K, et al. Neurons limit angiogenesis by titrating VEGF in retina. *Cell.* 2014;159(3):584-96.
4. Hakanpaa L, et al. Endothelial destabilization by angiopoietin-2 via integrin beta1 activation. *Nat Commun.* 2015;6:5962.
5. Andrade J, et al. Control of endothelial quiescence by FOXO-regulated metabolites. *Nat Cell Biol.* 2021;23(4):413-23.
6. Love MI, et al. Moderated estimation of fold change and dispersion for RNA-seq data with DESeq2. *Genome Biol.* 2014;15(12):550.
7. Garcia-Alonso L, et al. Benchmark and integration of resources for the estimation of human transcription factor activities. *Genome Res.* 2019;29(8):1363-75.
8. Tiwari PB, et al. Analyzing surface plasmon resonance data: choosing a correct biphasic model for interpretation. *Rev Sci Instrum.* 2015;86(3):035001.
9. Kabsch W. Xds. *Acta Crystallogr D Biol Crystallogr.* 2010;66(Pt 2):125-32.
10. Agirre J, et al. The CCP4 suite: integrative software for macromolecular crystallography. *Acta Crystallogr D Struct Biol.* 2023;79(Pt 6):449-61.
11. Liebschner D, et al. Macromolecular structure determination using X-rays, neutrons and electrons: recent developments in Phenix. *Acta Crystallogr D Struct Biol.* 2019;75(Pt 10):861-77.
12. Emsley P, et al. Features and development of Coot. *Acta Crystallogr D Biol Crystallogr.* 2010;66(Pt 4):486-501.
13. Manalastas-Cantos K, et al. ATSAS 3.0: expanded functionality and new tools for small-angle scattering data analysis. *J Appl Crystallogr.* 2021;54(Pt 1):343-55.
14. Forster S, et al. Scatter: software for the analysis of nano- and mesoscale small-angle scattering. *Journal of Applied Crystallography.* 2010;43(3):639-46.
15. Leppanen VM, et al. Characterization of ANGPT2 mutations associated with primary lymphedema. *Sci Transl Med.* 2020;12(560).

16. Yan Y, et al. The HDOCK server for integrated protein-protein docking. *Nat Protoc.* 2020;15(5):1829-52.

17. Manders EMM, et al. Measurement of co-localization of objects in dual-colour confocal images. *J Microsc.* 1993;169(3):375-82.

18. Bolte S, and Cordelieres FP. A guided tour into subcellular colocalization analysis in light microscopy. *J Microsc.* 2006;224(Pt 3):213-32.

19. Leman JK, et al. Macromolecular modeling and design in Rosetta: recent methods and frameworks. *Nat Methods.* 2020;17(7):665-80.

20. Webb B, and Sali A. Comparative Protein Structure Modeling Using MODELLER. *Curr Protoc Protein Sci.* 2016;86:2 9 1-2 9 37.

21. Jurrus E, et al. Improvements to the APBS biomolecular solvation software suite. *Protein Sci.* 2018;27(1):112-28.

22. Case DA, et al. The Amber biomolecular simulation programs. *J Comput Chem.* 2005;26(16):1668-88.

23. Hopkins CW, et al. Long-Time-Step Molecular Dynamics through Hydrogen Mass Repartitioning. *J Chem Theory Comput.* 2015;11(4):1864-74.

24. Abraham M, et al. GROMACS: High performance molecular simulations through multi-level parallelism from laptops to supercomputers,. *SoftwareX,.* 2015;Volumes 1–2,(ISSN 2352-7110):Pages 19-25.

25. Tian C, et al. ff19SB: Amino-Acid-Specific Protein Backbone Parameters Trained against Quantum Mechanics Energy Surfaces in Solution. *J Chem Theory Comput.* 2020;16(1):528-52.

26. Izadi S, et al. Building Water Models: A Different Approach. *J Phys Chem Lett.* 2014;5(21):3863-71.

27. Joung IS, and Cheatham TE, 3rd. Determination of alkali and halide monovalent ion parameters for use in explicitly solvated biomolecular simulations. *J Phys Chem B.* 2008;112(30):9020-41.

28. Hess B. P-LINCS: A Parallel Linear Constraint Solver for Molecular Simulation. *J Chem Theory Comput.* 2008;4(1):116-22.

29. Essmann U, et al. A smooth particle mesh Ewald method. *The Journal of Chemical Physics.* 1995;103(19):8577-93.

30. Berendsen HJC, et al. Molecular dynamics with coupling to an external bath. *The Journal of Chemical Physics.* 1984;81(8):3684-90.

31. Nosé S. A molecular dynamics method for simulations in the canonical ensemble. *Molecular Physics.* 1984;52(2):255–68.

32. Hoover WG. Canonical dynamics: Equilibrium phase-space distributions. *Phys Rev A Gen Phys.* 1985;31(3):1695-7.

33. Parrinello M, and Rahman A. Polymorphic transitions in single crystals: A new molecular dynamics method. *Journal of Applied Physics.* 1981;52(12):7182-90.

34. Barlow KA, et al. Flex ddG: Rosetta Ensemble-Based Estimation of Changes in Protein-Protein Binding Affinity upon Mutation. *J Phys Chem B.* 2018;122(21):5389-99.

35. Lyskov S, and Gray JJ. The RosettaDock server for local protein-protein docking. *Nucleic Acids Res.* 2008;36(Web Server issue):W233-8.

36. Barton WA, et al. Crystal structures of the Tie2 receptor ectodomain and the angiopoietin-2-Tie2 complex. *Nat Struct Mol Biol.* 2006;13(6):524-32.

37. Moore JO, et al. Dimerization of Tie2 mediated by its membrane-proximal FNIII domains. *Proc Natl Acad Sci U S A.* 2017;114(17):4382-7.

38. Leppanen VM, et al. Structural basis of Tie2 activation and Tie2/Tie1 heterodimerization. *Proc Natl Acad Sci U S A.* 2017;114(17):4376-81.

39. Schumacher S, et al. Structural insights into integrin alpha(5)beta(1) opening by fibronectin ligand. *Sci Adv.* 2021;7(19).

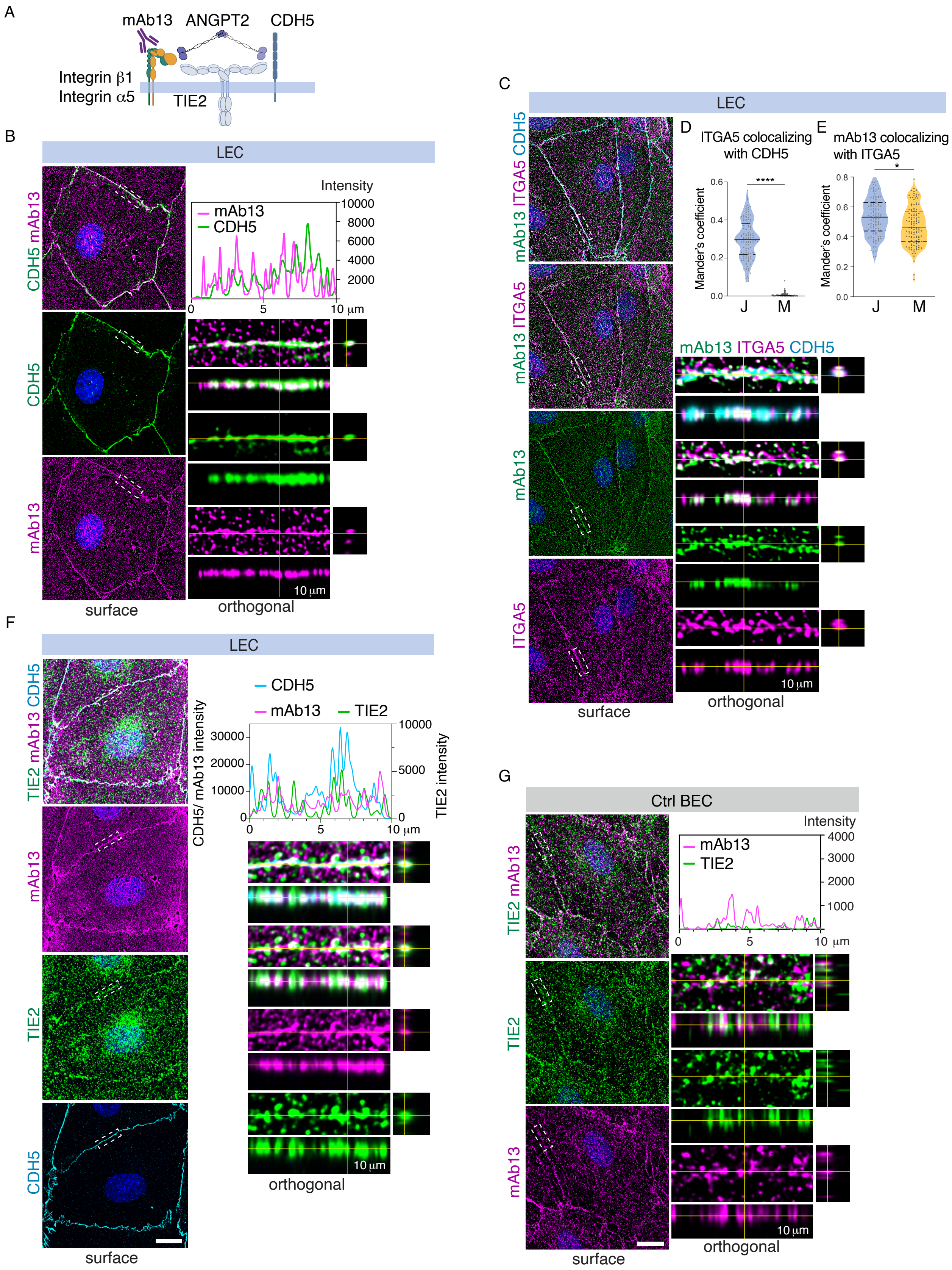
A
mAb13
ANGPT2
CDH5
Integrin β1
Integrin α5
TIE2
B
LEC
CDH5 mAb13
CDH5
mAb13
Intensity
mAb13
CDH5
10000
8000
6000
4000
2000
0
5
10 µm
10 µm
surface
orthogonal
C
LEC
mAb13 ITGA5 CDH5
mAb13 ITGA5
mAb13
ITGA5
mAb13 ITGA5 CDH5
10 µm
surface
orthogonal
D
ITGA5 colocalizing with CDH5
Mander's coefficient
****
J
M
E
mAb13 colocalizing with ITGA5
Mander's coefficient
*
J
M
F
LEC
TIE2 mAb13 CDH5
mAb13
TIE2
CDH5
CDH5
mAb13
TIE2
CDH5/ mAb13 intensity
TIE2 intensity
30000
20000
10000
0
10000
5000
0
0
5
10 µm
10 µm
surface
orthogonal
G
Ctrl BEC
TIE2 mAb13
TIE2
mAb13
Intensity
mAb13
TIE2
4000
3000
2000
1000
0
5
10 µm
10 µm
surface
orthogonal

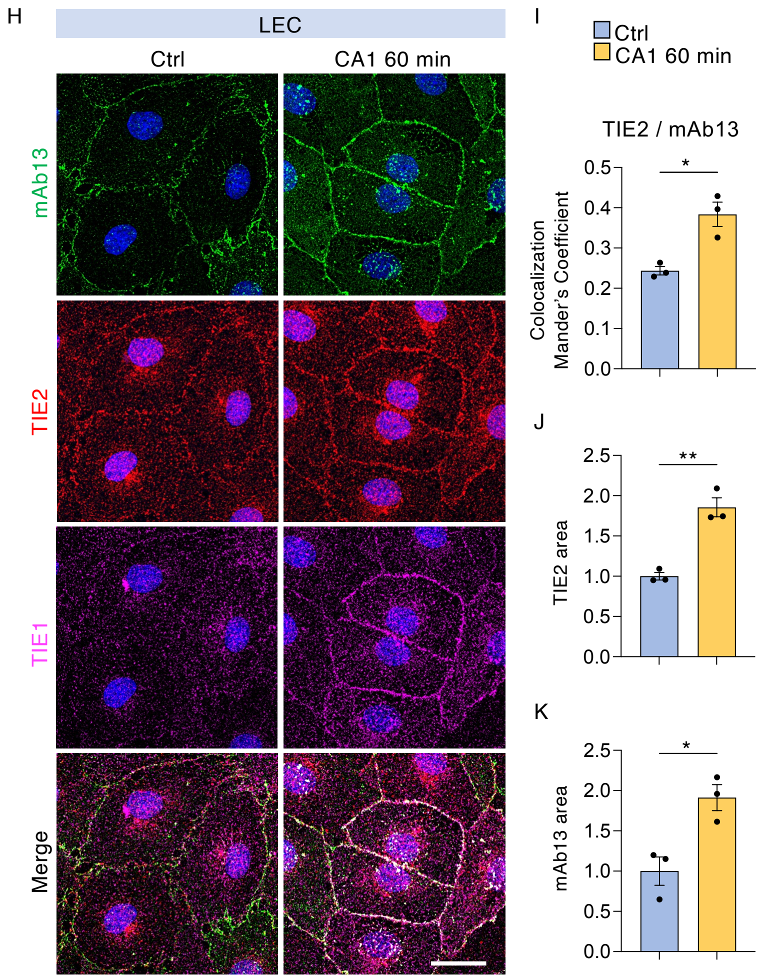

Supplemental Figure 1. Inactive β1-integrin colocalizes with VE-cadherin and α5-integrin in EC junctions. (**A**) Schematic representation of inactive β1-integrin with mAb13 antibody, α5-integrin (ITGA5), angiopoietin-2 (ANGPT2), TIE2 and VE-cadherin (CDH5). (**B**) Surface staining of human dermal lymphatic endothelial cells (LEC) for VE-cadherin and inactive β1-integrin. Quantification of Mander's overlap coefficient (MOC) for mAb13 / CDH5 colocalization is presented in Figure 1C. (**C**-**E**) Surface staining of α5-integrin, inactive β1-integrin and VE-cadherin in LECs (**C**). MOC for ITGA5 / CDH5 colocalization (**D**) and for mAb13 / ITGA5 colocalization (**E**) in junctions (J) and membrane (M) outside of junctions. A representative experiment is shown, 150 regions of interest quantified per sample. (**F**) Surface staining of LECs for CDH5, mAb13 and TIE2 (quantification shown in Figure 1H). (**G**) Surface staining for mAb13 and TIE2 in control (Ctrl) treated BECs (quantification shown in Figure 1E-G). (**H**-**K**) Staining of inactive β1-integrin (mAb13), TIE2 and TIE1 on cell surface in control or COMP-Ang1 (CA1)-stimulated LECs (**H**). MOC for TIE2 / mAb13 colocalization (**I**), and TIE2 and mAb13 area in junctions in Ctrl vs CA1 stimulated cells (**J** and **K**). n=3 independent experiments. Confocal Z-stack projections shown (**B, C, F, G, H**). Pixel intensity profile along the junction (horizontal line in xy ortogonal view) (**B, F, G**). Scale bars 10 μm (**B, C, F, G**; magnified area corresponds to 10 μm), 20 μm (**H**). Kruskal-Wallis test with Dunn's multiple comparisons test (**D** and **E**). Two-tailed t test (**I**-**K**). Data are mean ± SEM.

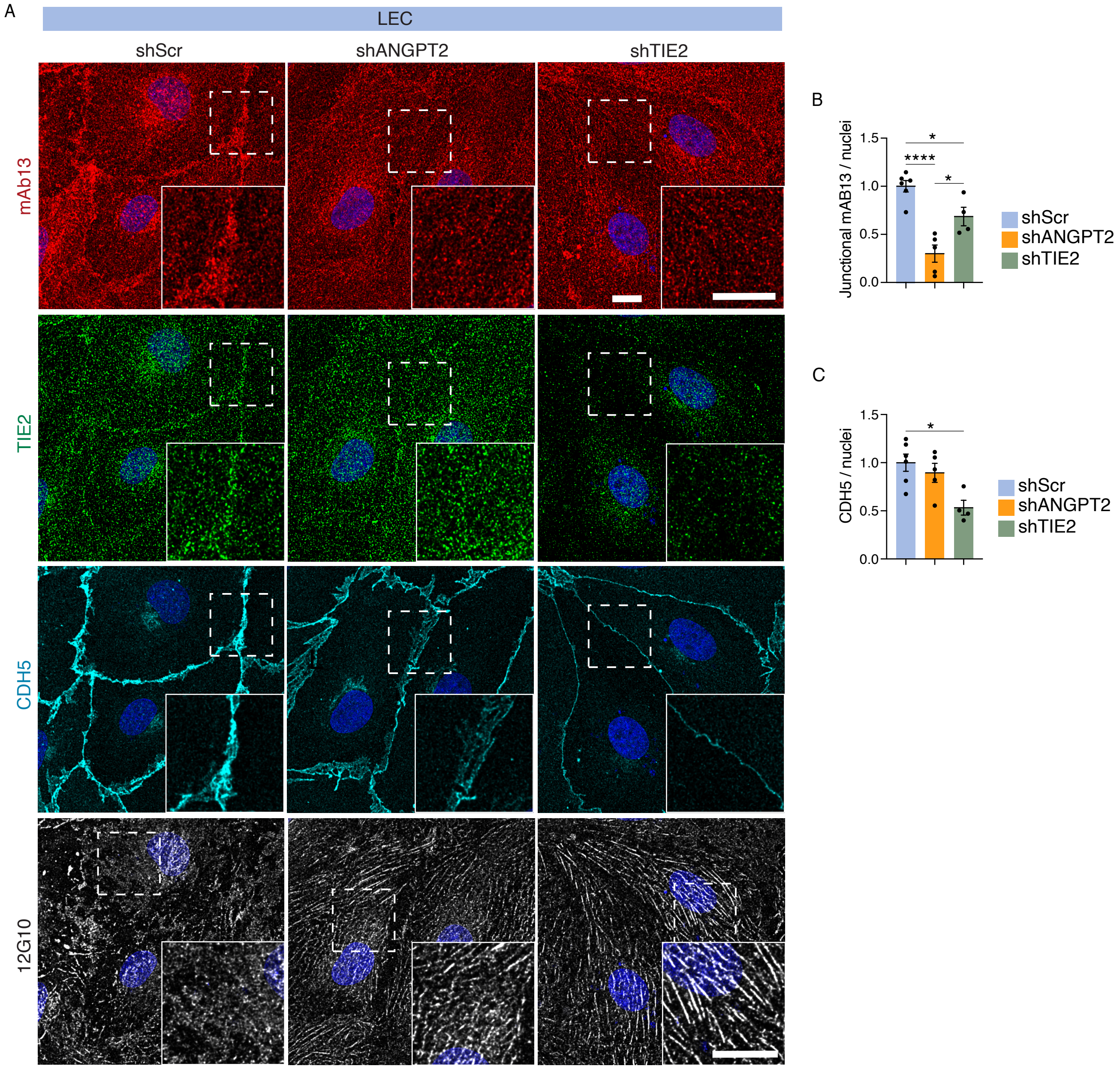

Supplemental Figure 2. Silencing of ANGPT2 or TIE2 decreases inactive β1-integrin in cell junctions. (**A**-**C**) LECs silenced for ANGPT2 (shANGPT2), TIE2 (shTIE2) or control (shScr) were stained for TIE2, CDH5, inactive (mAb13) and active β1-integrin (12G10) (**A**). Quantification of junctional mAb13 (**B**) and CDH5 (**C**) in LEC junctions. n=6 independent experiments for shScr, n=5 for shANGPT2, n=4 for shTIE2. Scale bars: 20 μm (**A**). Confocal Z-stack projections shown. One-way ANOVA with Tukey's post-hoc test (**B** and **C**). Data are mean ± SEM; *P < 0.05, ***P < 0.001.

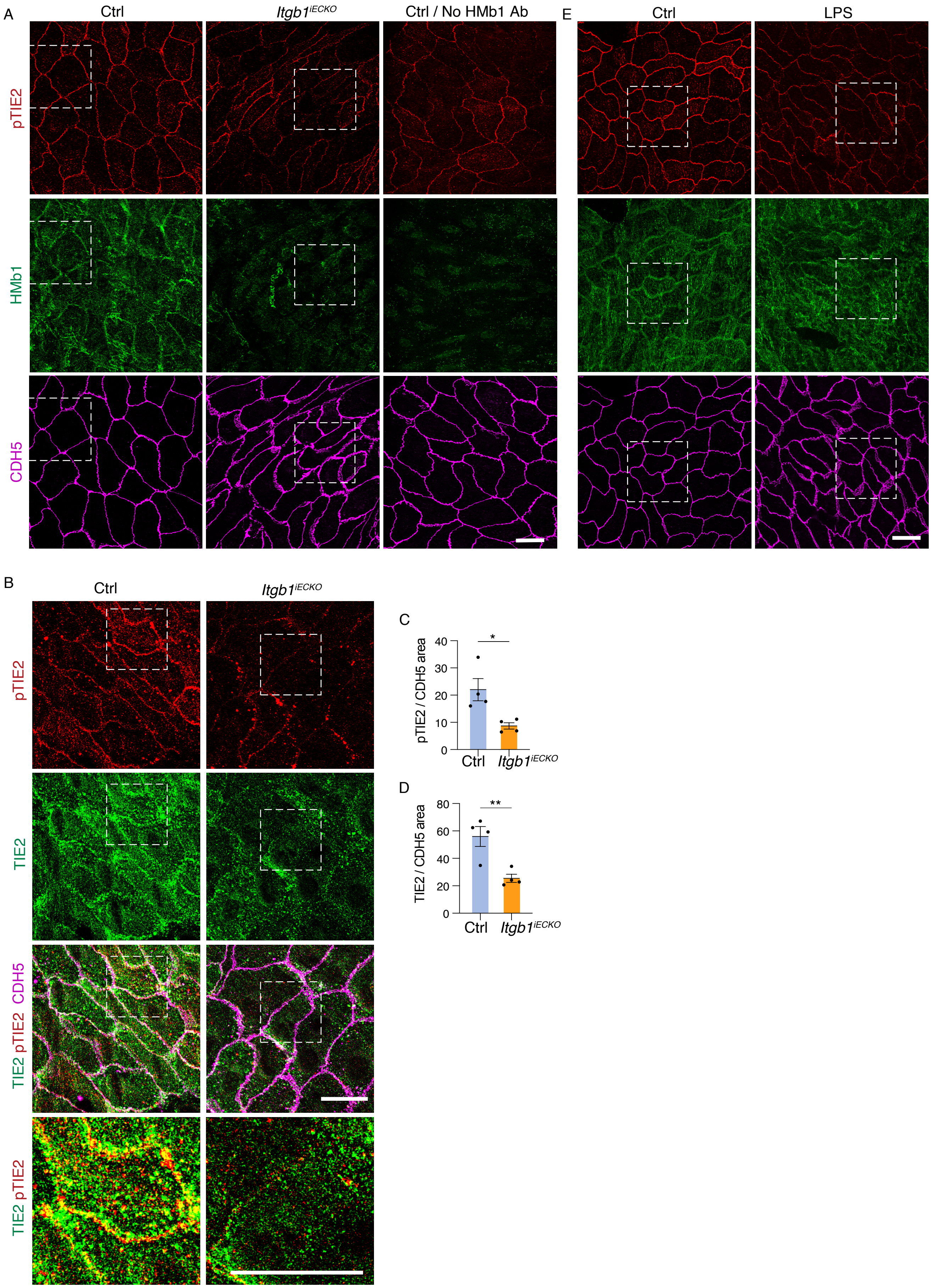

A
Ctrl
Itgb1iECKO
Ctrl / No HMb1 Ab
pTIE2
HMb1
CDH5
E
Ctrl
LPS
B
Ctrl
Itgb1iECKO
pTIE2
TIE2
TIE2 pTIE2 CDH5
TIE2 pTIE2
C
pTIE2 / CDH5 area
40
30
20
10
0
*
Ctrl
Itgb1iECKO
D
TIE2 / CDH5 area
80
60
40
20
0
**
Ctrl
Itgb1iECKO

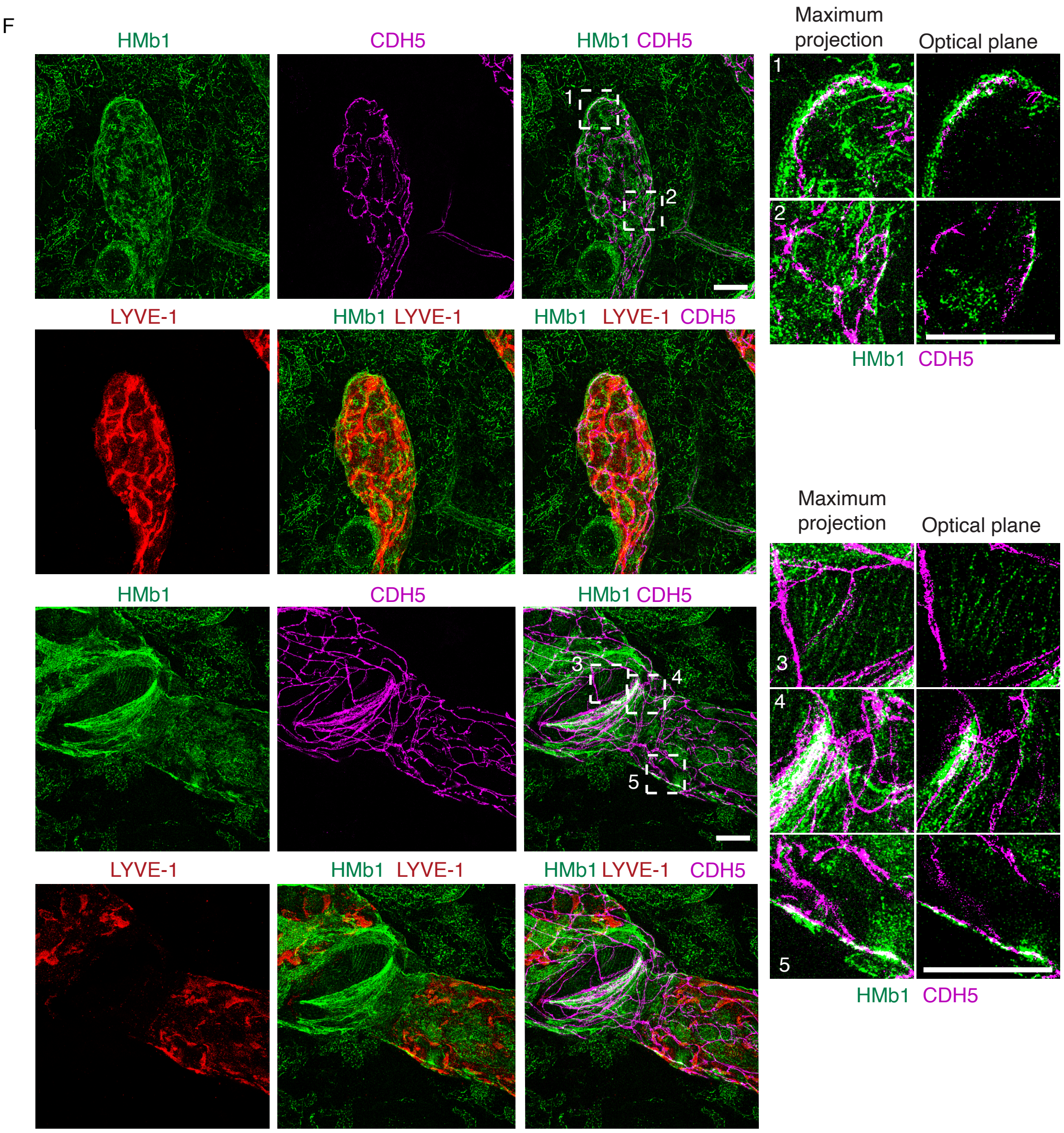

Supplemental Figure 3. Staining of pY992-TIE2 and β1-integrin in inferior vena cava from mice after β1-integrin deletion and endotoxemia, and in dermal lymphatic vessels. (**A-D**) Vena cava from *Itgb1*^{iECKO} (12 day deletion in adult mice) and control mice were stained for β1-integrin (HMb1), pY992-TIE2 (pTIE2), TIE2 and VE-cadherin (CDH5). Staining with only secondary antibody is shown as control (No HMb1 (**A**)). Quantification of total TIE2 and pTIE2 per CDH5 (**C** and **D**). (**E**) Vena cava from control mice and after LPS administration were stained for inactive β 1-integrin (HMb1), pTIE2 and CDH5. (**F**) Dermal lymphatic vessels in mouse ear were stained for β1-integrin (HMb1), LYVE-1 and VE-cadherin (CDH5). Scale bars: 20 μm (**A, B, E, F**). Confocal Z-stack projections, and optical planes, are shown as indicated. Two-tailed t test (**C** and **D**). Data are mean ± SEM; *P < 0.05, **P < 0.01. Magnified views of the images presented in A and E are shown in Figure 2B and G.

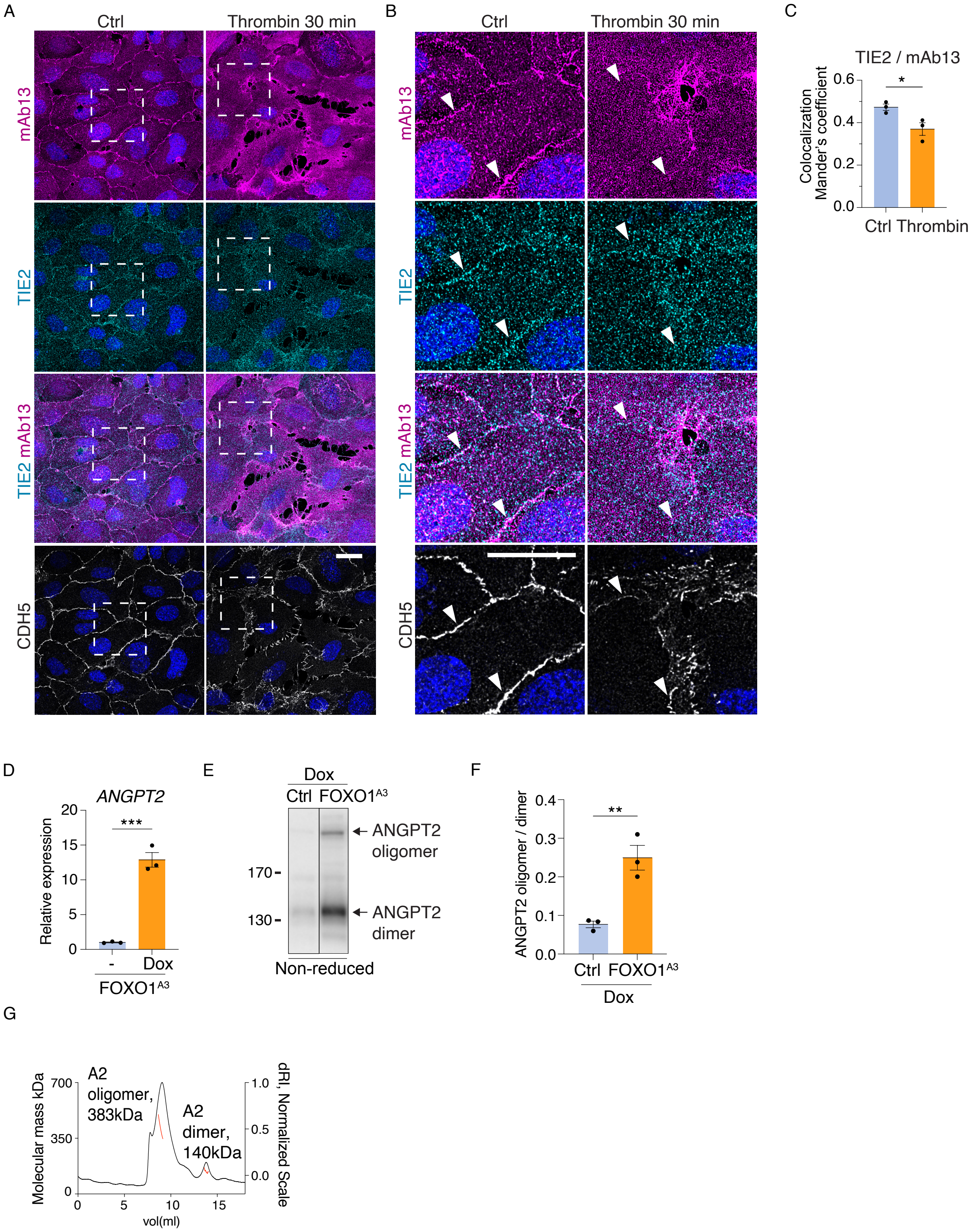

Supplemental Figure 4. Effect of thrombin on TIE2 and inactive β1-integrin colocalization in EC junctions and analysis of ANGPT2 oligomer. (**A** and **B**) LECs were stimulated with thrombin, and stained for cell surface TIE2, inactive β1-integrin (mAb13) and VE-cadherin (CDH5). Region marked in **A** is magnified in (**B**). (**C**) Mander's overlap coefficient (MOC) for TIE2 / mAb13 colocalization in junctions. (**D**) Doxycycline (Dox) -induced expression of a constitutively nuclear FOXO1^{A3} mutant in HUVEC increased *ANGPT2* mRNA expression. RT-qPCR analysis of transfected HUVEC lysates. (**E** and **F**) Immunoblotting of conditioned media from Dox-induced FOXO1^{A3} and control HUVECs under non-reducing conditions (**E**). Lanes were run on the same gel but were noncontiguous. Quantification of ANGPT2 (A2) oligomer to dimer ratio (**F**). (**G**) Affinity purified full-length recombinant ANGPT2 (produced in CHO cells) was subjected to SEC-MALS using a Superdex 200 Increase 10/300 column, resulting in oligomeric (Peak 1) and dimeric (Peak 2) ANGPT2 species. Representative image of two independent experiments. Measured molecular weights are indicated. Confocal Z-stack projections shown. Scale bars: 20 μm (**A** and **B**). 300 regions of interest per sample (**C**). n = 3 independent experiments. Two-tailed unpaired t-test (**C**, **D** and **F**). Data are mean ± SEM. $P < 0.05$*, <0.01**, <0.001***.

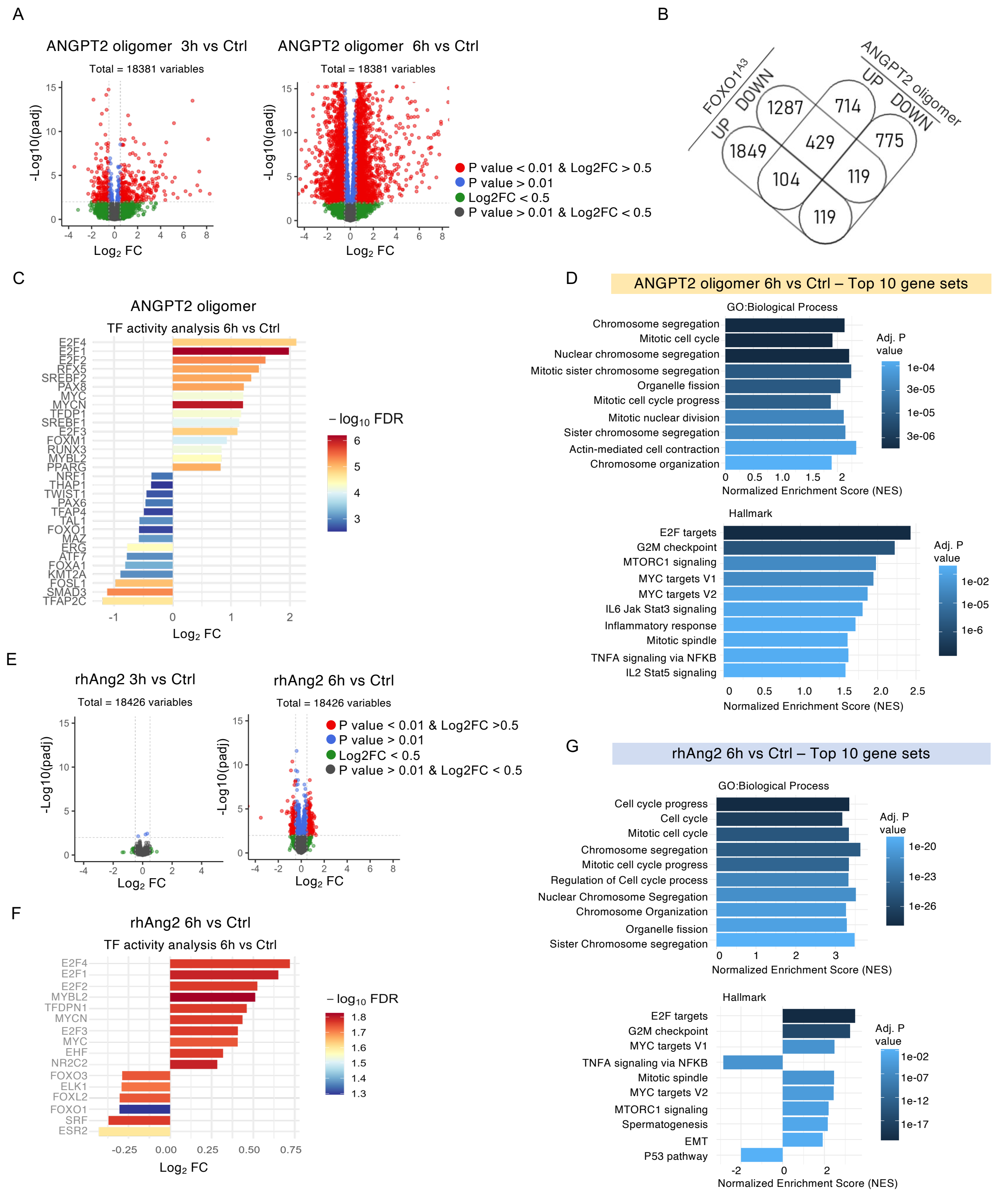

Supplemental Figure 5. ANGPT2 oligomer-induced gene expression in LECs. (**A**-**G**) Bulk mRNA sequencing of LECs stimulated for 3 hours or 6 h with ANGPT2 oligomer (**A**-**D**) or rhAng2 (**E**-**G**). Volcano plots of differentially expressed genes (DEGs) at 3 h and 6 h after ANGPT2 oligomer (**A**) or rhAng2 (**E**) stimulation vs control. Genes with adjusted p-value < 0.01 and absolute $\log_2$ fold change (FC) > 0.5 were considered significantly differentially expressed. The dashed line denotes the significance threshold. (**B**) Venn diagram illustrating the overlap of DEGs between HUVECs expressing constitutively nuclear FOXO1^{A3} mutant at 16 hours (reanalysis of GSE128634) and ANGPT2 oligomer–stimulated LECs relative to control (Ctrl) (6h, own data set). DEGs were identified using DESeq2 with an adjusted p-value < 0.00001 and absolute $\log_2$ FC > 0.5, according to Kraft et al. (1). (**C**, **F**) Virtual Inference of Protein-Activity by Enriched Regulon (VIPER) analysis using bulk RNASeq data from LECs stimulated with oligomeric ANGPT2 (**C**) or rhAng2 (**F**) for 6 h (absolute $\log_2$ fold change > 0.25; adjusted p-value < 0.05 (adj P value for FOXO1 is 0.0508 in F), ranked by adj P value, visualization of maximum of top 15 candidates. (**D**-**G**) Gene set enrichment analysis of ANGPT2 oligomer (**D**) or rhAng2 (**G**) stimulated LECs for 6 hours vs control. Top 10 enriched gene sets from Gene Ontology (GO) Biological Process terms and Hallmark gene sets are shown, ranked by adjusted P value and encoded by a color gradient. To control the false discovery rate (FDR), the Benjamini–Hochberg procedure was applied to the VIPER score comparisons across TFs (**C**, **F**). TF, transcription factor

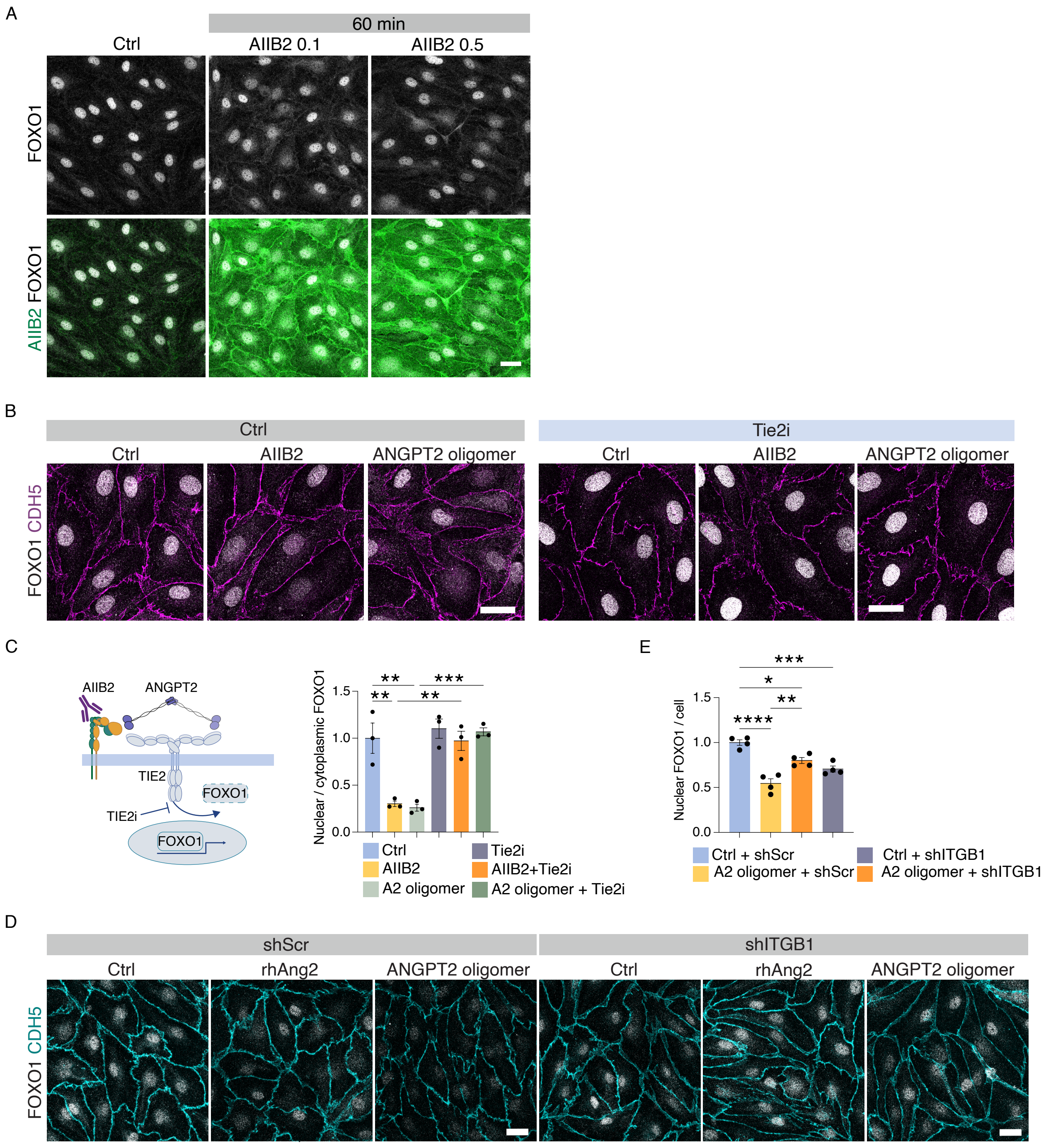

Supplemental Figure 6. Effect of AIIB2 antibody, TIE2 inhibitor and β1-integrin silencing on FOXO1. (**A**) LECs stimulated with AIIB2 antibody (ligand blocking β1-integrin antibody) for 60 min were stained for FOXO1 and AIIB2 (secondary antibodies only). Quantification of nuclear to cytoplasmic FOXO1 ratio normalized to nuclei is presented in Figure 4E. (**B**) Staining of FOXO1 and VE-cadherin (CDH5) in LECs treated with AIIB2 or ANGPT2 (A2) oligomer ± TIE2 inhibitor (TIE2i, BAY-826). (**C**) Quantification of nuclear vs cytoplasmic FOXO1 ratio. (**D**) Staining of FOXO1 and VE-cadherin (CDH5) in LECs silenced for Scramble control (shScr) or β1-integrin (shITGB1) and subsequently treated with rhAng2 (R&D) or A2 oligomer. (**E**) Quantification of nuclear vs cytoplasmic FOXO1 ratio. Scale bars: 25 µm (**A, B, D**). Confocal Z-stack projections shown. One-way ANOVA with Tukey's post hoc test (**C, E**). n = 3 (**C**) and 4 (**E**) independent experiments. Data are mean ± SEM; *P < 0.05, **P < 0.01, ***P < 0.001, ****P < 0.0001.

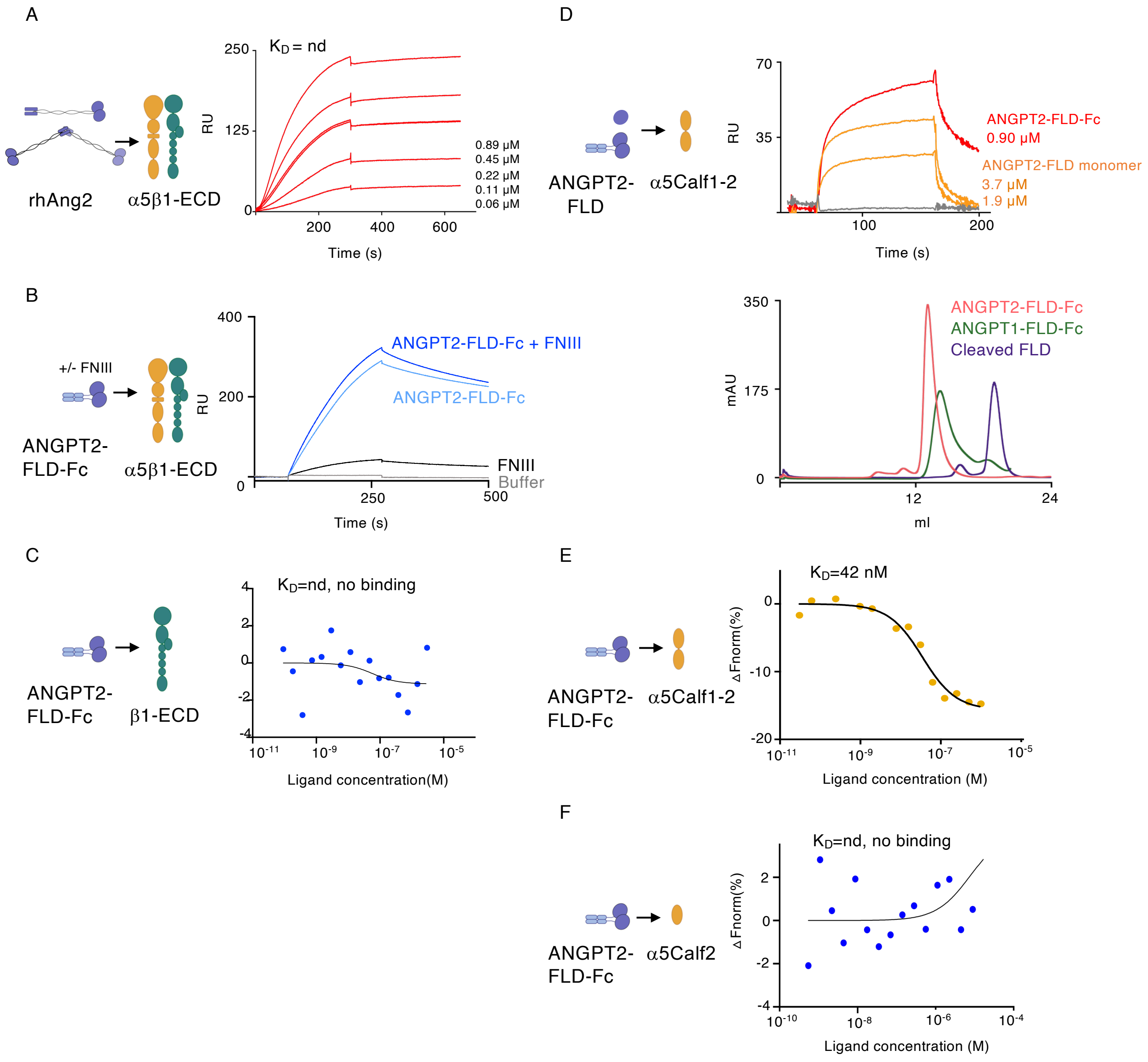

Supplemental Figure 7. Binding of ANGPT2 to α5β1-integrin ectodomains. (**A**) Binding of rhAng2 to immobilized α5β1-ectodomain (α5β1-ECD) in SPR in the presence of 2 mM $MnCl_2$ and 2 mM $MgCl_2$. (**B**) Binding of ANGPT2-FLD-Fc (0.04 μM) to immobilized α5β1-ECD in the presence or absence of fibronectin type III repeats 7-10 (FNIII) (0.003 μM), as indicated. The binding of FNIII to α5β1-ECD induces a slight increase in resonance units (RU) (demonstrating binding), when analyzed alone or in combination with ANGPT2-FLD-Fc. (**C**) MST binding assay of labelled ANGPT2-FLD-Fc to β1-ECD shows no binding. (**D**) Binding of proteolytically cleaved, monomeric ANGPT2-FLD and dimeric ANGPT2-FLD-Fc to immobilized α5Calf1-2. SEC elution profile of dimeric ANGPT2-FLD-Fc, and Factor Xa cleaved monomeric ANGPT2 FLD, as well as ANGPT1-FLD-Fc, using Superdex 200 Increase 10/300. (**E**) Binding of fluorescently labelled α5Calf1-2 domain to ANGPT2-FLD-Fc in MST with average affinity of K_D 42 nM (n=3 independent experiments). (**F**) MST binding assay of labelled ANGPT2-FLD-Fc to α5Calf-2 shows no binding. Representative binding curves of experiments repeated at least three times are shown. SPR, surface plasmon resonance; MST, microscale thermophoresis.

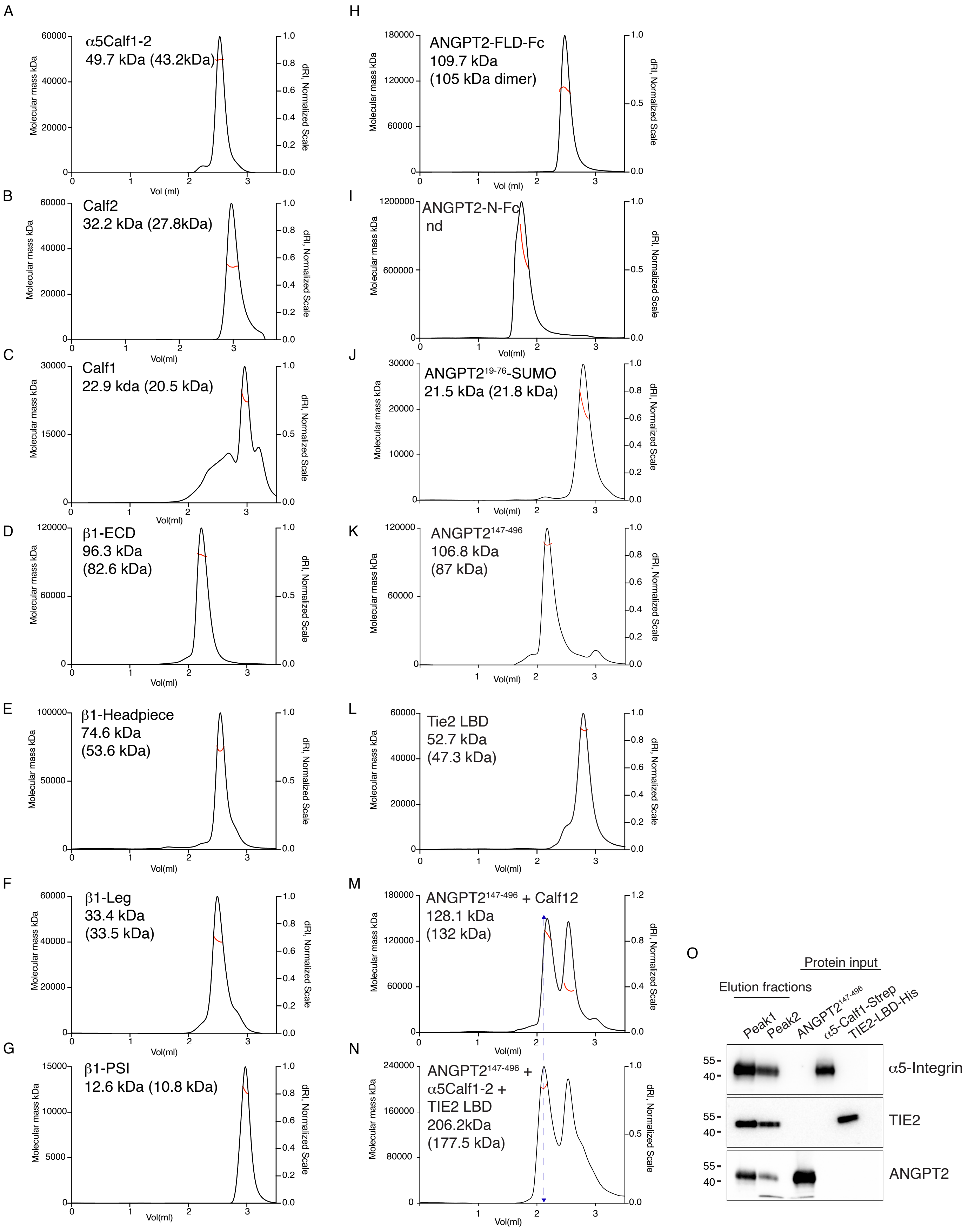

Supplemental Figure 8. SEC-MALS analysis of recombinant proteins and protein complexes. (**A**-**L**) Recombinant proteins, as indicated, were purified from insect cell conditioned media using affinity chromatography and subsequently using size-exclusion chromatography, and analysed in SEC-MALS using BioSEC 300 4.6x300. Molecular mass and refractive index in arbitary units (dRI) are plotted as a function of elution volume. Determined and theoretical (in parentheses) molecular masses are shown. nd, not determined. (**M** and **N**) For analysis of protein complexes, proteins were mixed in equal ratios and analyzed in SEC-MALS using BioSEC 300 4.6x300. The difference in elution volumes for the double (**M**) and trimeric complexes (**N**) is indicated by a dashed line, corresponding to difference in the observed and theoretical molecular masses. (**O**) Western blot analysis of elution fractions of the peak 1 and peak 2 in SEC-MALS in **N** with single protein controls, confirming trimeric complex formation. Proteins elute also in peak2, but as lower molecular mass complexes. Molar mass calculated across the peak (red line).

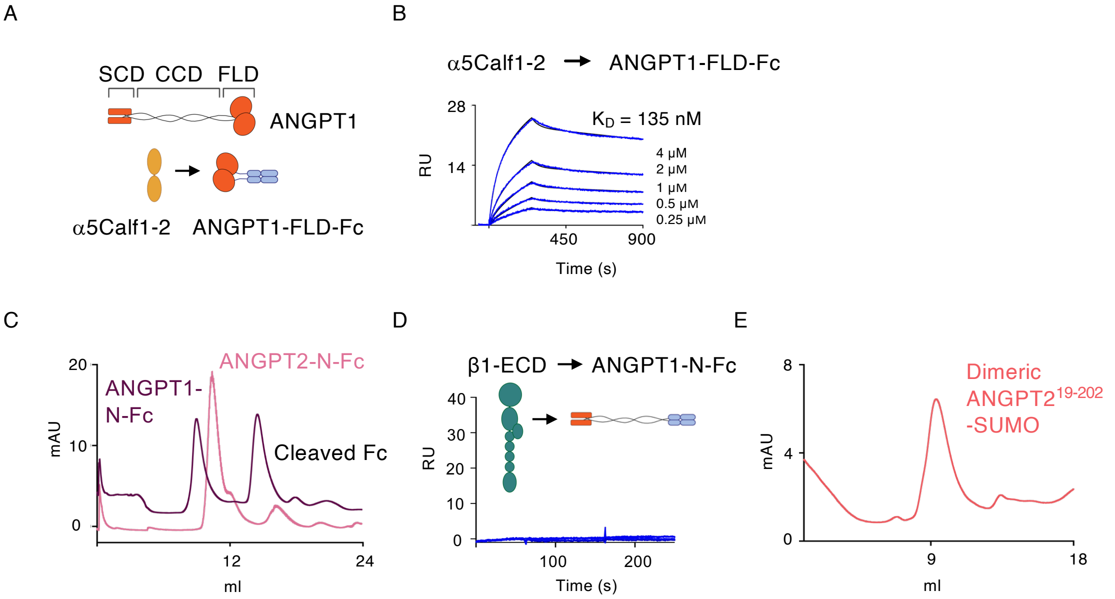

Supplemental Figure 9. Interaction of ANGPT1-FLD with Calf1-2 in SPR and SEC elution profiles of N-terminal ANGPT proteins. (**A** and **B**) Binding of α5Calf1-2 to immobilized ANGPT1-FLD-Fc in SPR in indicated concentrations. K_D 135 nM. n= 3 independent experiments. (**C**) SEC elution profile for dimeric ANGPT1-N-Fc and ANGPT2-N-Fc, shown is also cleaved Fc, using Superdex 200 Increase. (**D**) Assessment of binding of β1-integrin ectodomain (β1-ECD) at concentrations of 0.01, 0.02, 0.04, 0.08 and 0.17 μM to immobilized ANGPT1 N-terminal domain (ANGPT1-N-Fc) using SPR. No binding is detected at any of the concentrations. (**E**) Insect cell -purified dimeric ANGPT2$^{19\text{-}202}$-SUMO analyzed using size-exclusion chromatography (SEC), using Superdex 200 Increase 10/300 column' (bed volume: 24 mL). Representative of three independent experiments is shown. SPR, surface plasmon resonance.

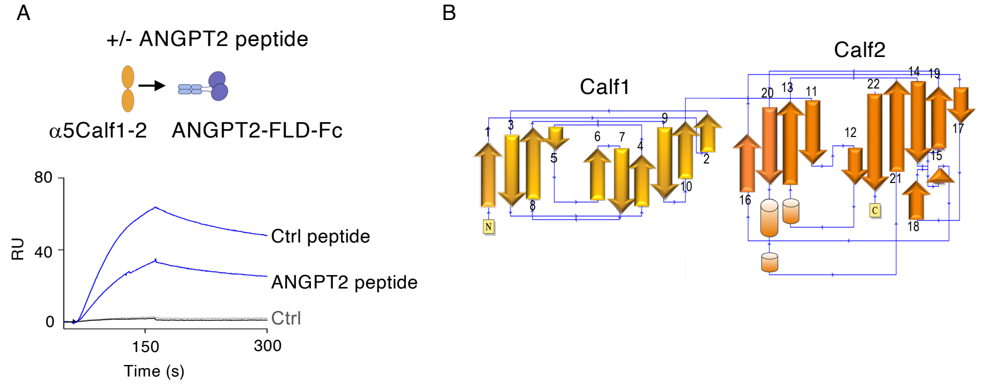

Supplemental Figure 10. (**A**) Binding of α5Calf1-2 (1.7 μM) to immobilized ANGPT2-FLD-Fc in the presence of an ANGPT2 (residues E352-Q366 in human ANGPT2) or control scrambled peptide (8.7 μM each) in SPR. A representative experiment is shown. n=3 independent experiments. Peptides were mixed with α5Calf1-2 before injection. E352-Q366 peptide inhibits α5Calf1-2 binding to ANGPT2. (**B**) Topological presentation of α5Calf1-2, based on crystal structure in Figure 6A. Calf1 domain is an all-β structure with 10 β-strands separated by 10 loops, whereas the Calf2 domain consists of 12 β-strands separated by 8 loops and 2 short α-helices. SPR, surface plasmon resonance.

A

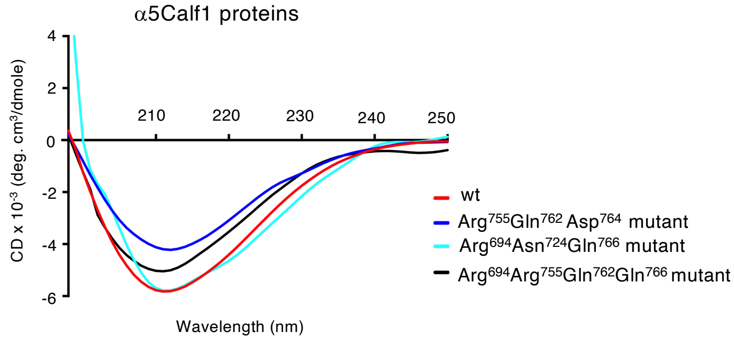

B

```
        N724                               R755      Q762 D764 Q766
ITGA5   NQSRLLVCDLGNPMKAGASLWGGLRFTVPHLRDTKKTIQFDFQI 767
ITGAV   NQTRQVVCDLGNPMKAGTQLLAGLRFSVHQQSEMDTSVKFDLQI 747
ITGA2B  NETRVVLCELGNPMKKNAQIGIAMLVSVGNLEEAGESVSFQLQI 754
ITGA8   NVTRMVVCDLGNPMVSGTNYSLGLRFAVPRLEKTNMSINFDLQI 762
        * :* ::*:*****  .:.   .: .:* .  .   ::.*::**
```

Supplemental Figure 11. (**A**) Circular dichroism spectra of wildtype and mutant α5Calf1 proteins. Amino acid substitutions were created in α5Calf1, and mutant proteins were purified from insect cells and analysed using circular dichroism. All α5Calf1 mutants exhibited secondary structural elements and attained beta-pleated sheet conformation, indicating that the mutants retained structural integrity comparable to wild-type α5Calf1. (**B**) Sequence alignment of αCalf1 domains of selected human α-integrins using Clustal Omega.

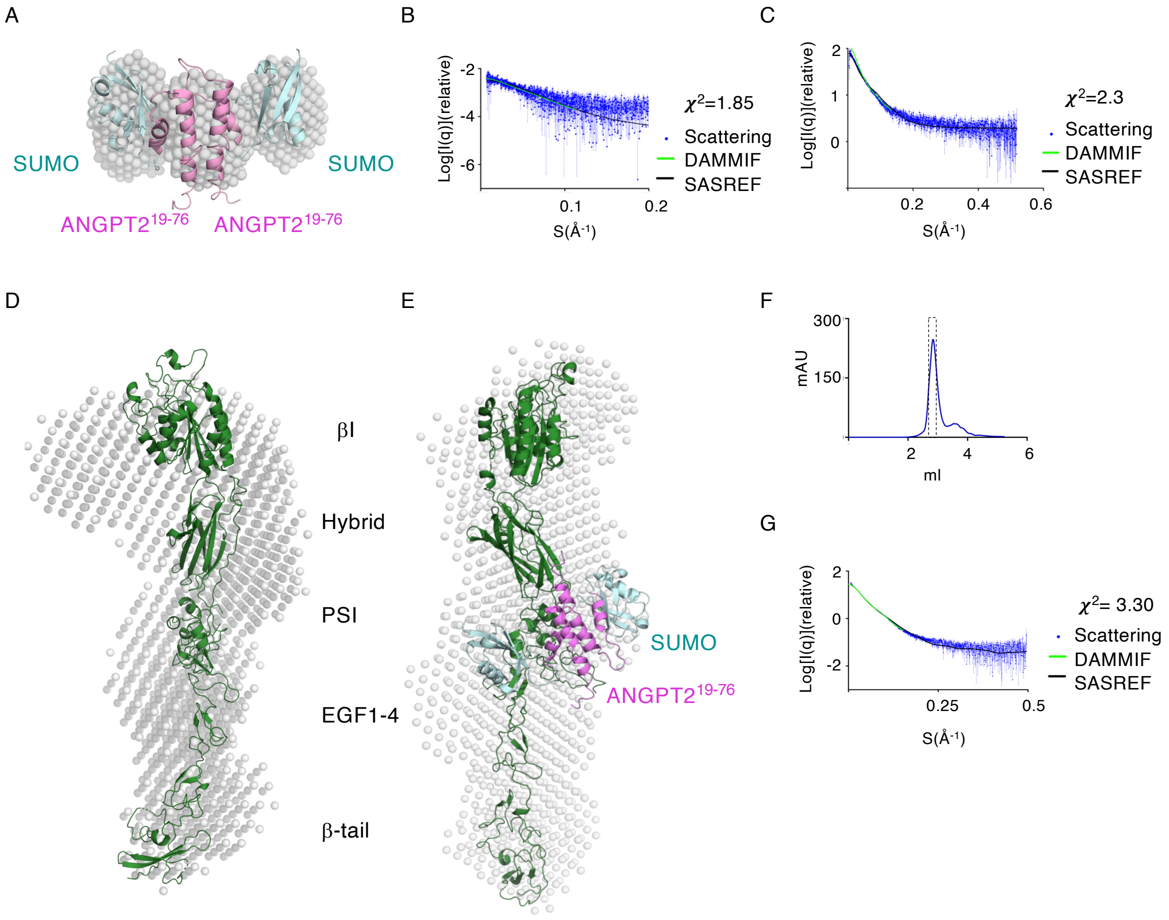

Supplemental Figure 12. SAXS analysis of β1-integrin ectodomain with ANGPT2$^{19-76}$ -SUMO. (**A** and **B**) SAXS analysis of ANGPT2$^{19-76}$-SUMO. Ab initio (determined using DAMMIF) and homology models (rigid-body refined using SASREF) (**A**). Fit of ab initio (green) and homology model (black) with SAXS data (blue) (**B**). (**C** and **D**) SAXS analysis of β1-integrin ectodomain (β1-ECD). Ab initio and homology models (**D**) and fit of ab initio (green) and homology model (black) with SAXS data (blue) (**C**). (**E**-**G**) SEC-SAXS analysis of ANGPT2$^{19-76}$ -SUMO in complex with β1-ECD. SEC-SAXS elution profile (**F**), overlay of Ab initio with homology models (**E**), and scattering intensities overlaid with the fit from SASREF and DAMMIF (**G**). β1-integrin domains are marked.

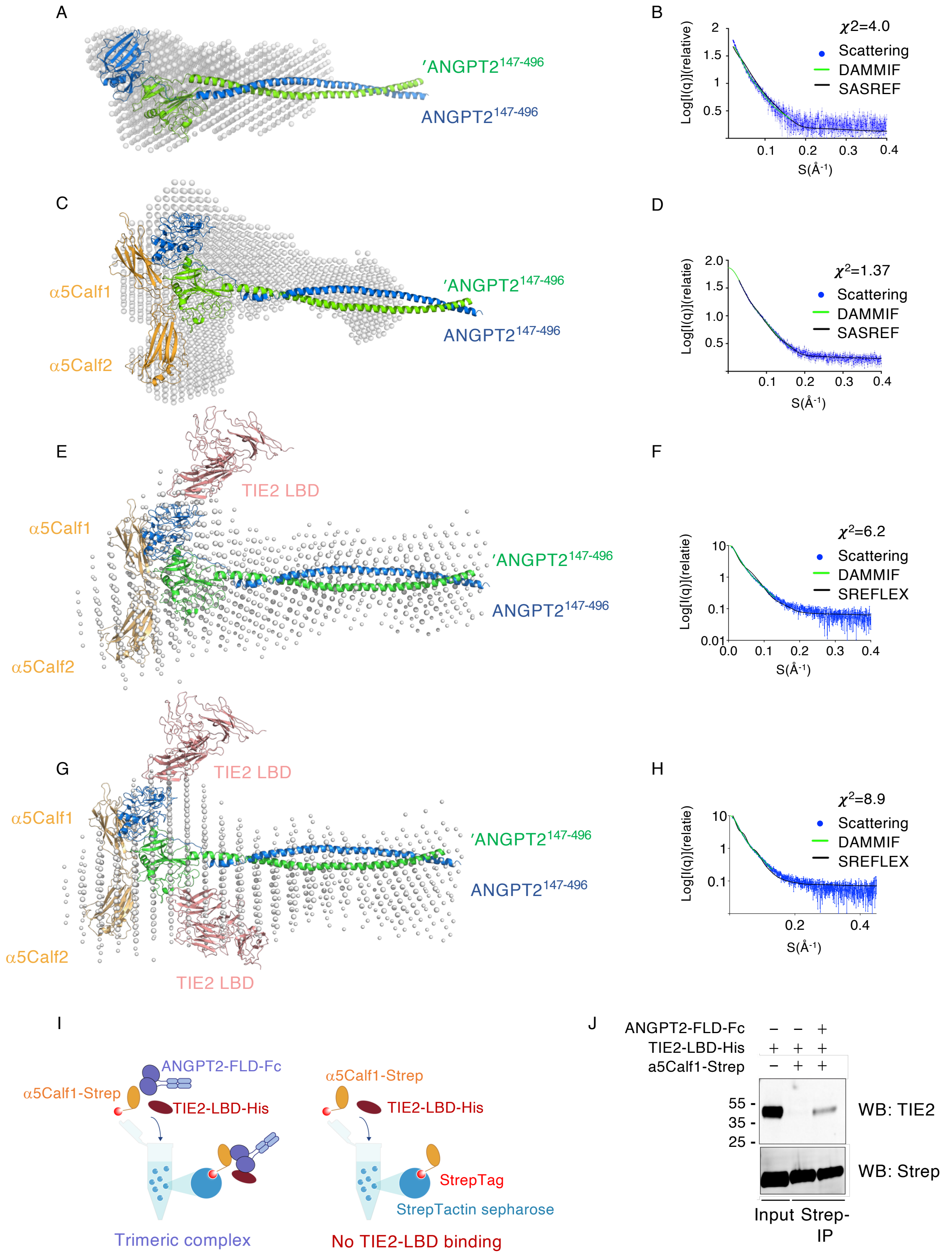

Supplemental Figure 13. SAXS and co-immunoprecipitation analysis of ANGPT2 complexes with α5-Calf1-2 and TIE2. (**A, B**) SAXS analysis of ANGPT2$^{147-496}$ (blue and green monomers). Rigid body modeling was performed using SASREF, based on Leppänen et al. (15) and superimposed with *ab initio* model calculated using DAMMIF (**A**). Fit of ab initio (green) and homology model (black) with SAXS data (blue) for ANGPT2$^{147-496}$ (**B**). (**C, D**) SAXS analysis (in batch mode) of ANGPT2$^{147-496}$ in complex with α5Calf1-2. Ab initio (DAMMIF) and homology models (SASREF) are shown (**C**). Fit of ab initio (green) and homology model (black) with SAXS data (blue) (**D**). (**E-H**) SEC-SAXS ab initio models of ANGPT2$^{147-496}$, α5Calf1-2 and TIE2 LBD complex, superimposed with homology models containing one (**E**) or two TIE2 ligand binding domains (LBDs) (**G**). The ab initio models were calculated using DAMMIF, and the homology models were rigid-body refined using SREFLEX. Fits of ab initio (green) and homology model (black) with SAXS data (blue) are shown for models with one (**F**) or two TIE2 LBDs (**H**) in the complex. (**I, J**) Coimmunoprecipitation analysis of pull-down for α5Calf1-Strep (Twin-Streptag) using StrepTactin sepharose. Note that TIE2 LBD does not bind α5Calf1-Strep, but co-immunorecipitates in the presence of ANGPT2-FLD-Fc, indicating trimeric complex formation. n=3 independent experiments. Representative experiment is shown.

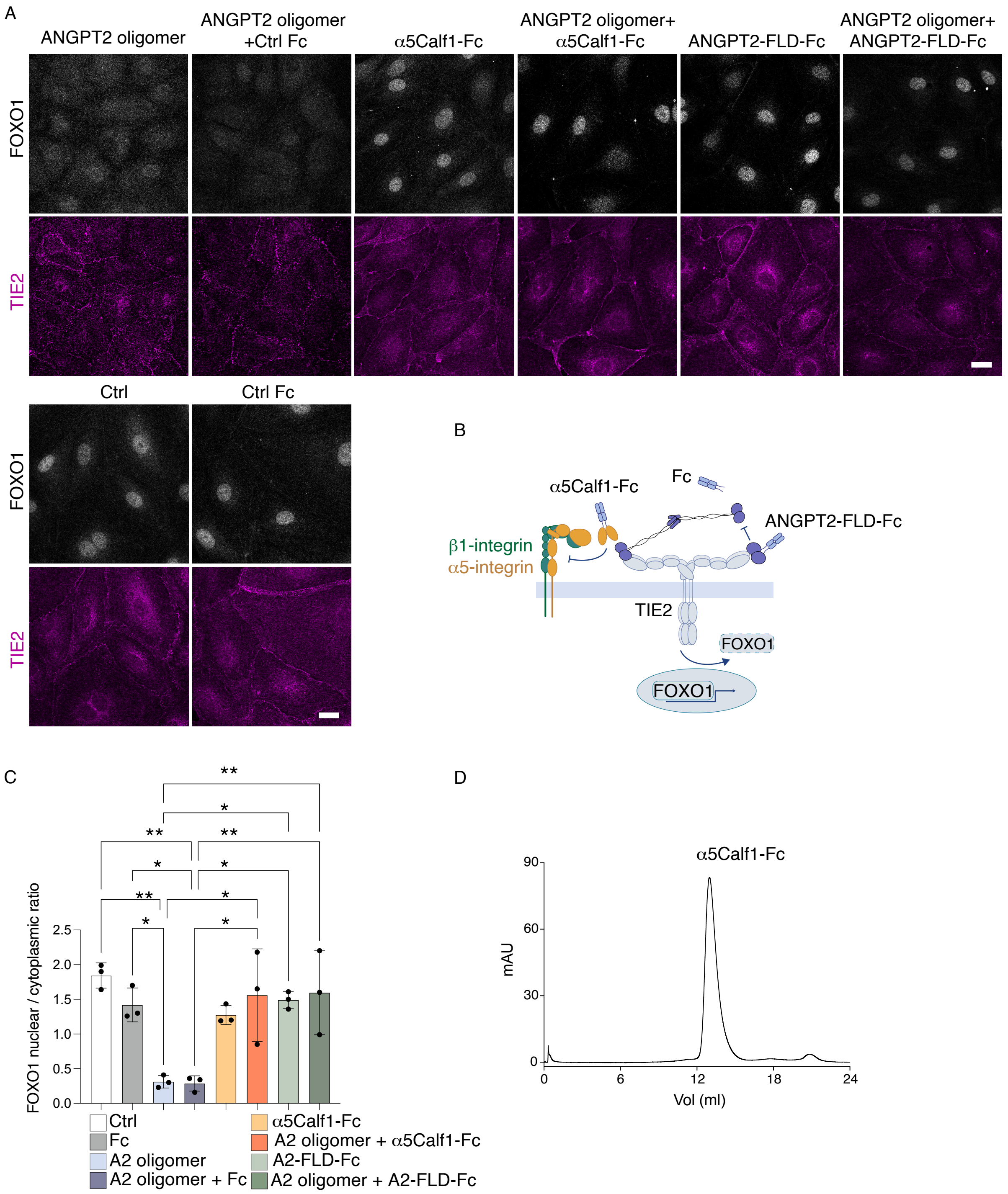

Supplemental Figure 14. α5Calf1-Fc inhibits oligomeric ANGPT2-induced FOXO1 nuclear exclusion. (**A-B**) Staining of FOXO1 and TIE2 in LECs treated with ANGPT2 (A2) oligomer in the presence of control Fc (Fc) or Fc fusion of α5Calf1 (α5Calf1-Fc) or ANGPT2 FLD (A2-FLD-Fc). (**C**) Quantification of nuclear vs cytoplasmic FOXO1 ratio. (**D**) α5Calf1-Fc and A2-FLD-Fc were purified from insect cell conditioned media using affinity chromatography and subsequently using size-exclusion chromatography (SEC). SEC elution profile for α5Calf1-Fc is shown using Superdex 200 Increase 10/300 column (bed volume: 24 mL). SEC-MALS analysis for A2-FLD-Fc is shown in Supplemental Figure **8H**. Scale bar: 10 μm (**A**). Confocal Z-stack projections shown. n = 3 independent experiments, one-way ANOVA with Tukey's post hoc test. Data are mean ± SD. *$P < 0.05$, **$P < 0.01$, ***$P < 0.001$.

Supplemental Table 1. Description of recombinant proteins used. Proteins were expressed using baculovirus or mammalian expression vectors* and produced and purified from insect cells or CHO cells*, respectively.

**Description**	**Recombinant protein**	**Amino acid residues** (according to human protein sequence)	**N-terminal tag**	**C-terminal tag**
α5-integrin Calf1 and Calf2 domains	α5Calf1-2	I640-G992	6xhis	Twin-streptag
α5-integrin Calf1 domain	α5Calf1	I640-E787	6xhis	Twin-streptag
α5-integrin Calf1 domain	α5Calf1	I640-E787	None	IgG-Fc
α5-integrin Calf2 domain	α5Calf2	A788-G992	6xhis	Twin-streptag
β1-integrin ectodomain	β1-ECD	Q21-D728	6xhis	Twin-streptag
β1-integrin headpiece	β1-head	N25-E476	6xhis	Twin-streptag
β1-integrin leg piece	β1-leg	C475-D729	6xhis	Twin-streptag
β1-integrin PSI domain	β1-PSI	Q21-P82, C36A	Flag-tag	Twin-streptag
CHO cell - produced full length ANGPT2*	ANGPT2	Y19-F496	none	Twin-streptag
ANGPT2 fibrinogen-like + coiled coil domains	$ANGPT2^{147-496}$	A147-F496	6xhis	none
ANGPT2 fibrinogen-like domain	ANGPT2-FLD	K275-F496	None	IgG-Fc
ANGPT2 N-terminal domain	$ANGPT2\text{-}N^{19-248}$	Y19-Q248	None	IgG-Fc
ANGPT2 superclustering + coiled coil domain	$ANGPT2^{19-202}$	Y19-K202	6xHis-SUMOStar	Twin-streptag
ANGPT2 superclustering domain	$ANGPT2^{19-76}$	Y19-S76	6xHis-SUMOStar	Twin-streptag
TIE2 ligand binding domain	TIE2-LBD	A22-P224	None	6xHis
ANGPT1 fibrinogen-like domain	ANGPT1-FLD	S255-D497	None	IgG-Fc
ANGPT1 N-terminal domain	$ANGPT1\text{-}N^{16-262}$	H16-V262	None	IgG-Fc
α5-integrin Calf1 domain with $R^{694}Q^{766}N^{724}$ substitutions	RQN-α5Calf1	I640-E787	6xhis	Twin-streptag
α5-integrin Calf1 domain with $R^{755}Q^{762}D^{764}$ substitutions	RQD-α5Calf1	I640-E787	6xhis	Twin-streptag
α5-integrin Calf1 domain with $R^{694}R^{755}Q^{762}Q^{766}$ substitutions	RRQQ-α5Calf1	I640-E787	6xhis	Twin-streptag

Supplemental Table 2. Summary of theoretical MWs and MWs determined using SEC-MALS and SAXS (KDa).

Protein / protein complex	**Theoretical**	**SEC-MALS** (BioSEC 300)	**SAXS $M_{wt.}$** (Batch mode)	**SEC-SAXS M_{wt}** (BioSEC 300)	**SEC-MALS** (S200 Increase)	**SEC S200 Increase**
α5Calf1-2	43.2	49.7	50.1			
α5Calf1	20.5	22.9				
α5Calf2	27.8	32.2				
α5Calf1-Fc	42.4 (monomer) 84.4 (dimer)					SFig. 14D
β1-ECD	82.6	96.3	86.3			
β1-head	53.3	74.6				
β1-leg	33.5	33.4				
β1-PSI	10.8	12.6				
ANGPT2 peak1 (oligomer)	58.5 (monomer) 117.0 (dimer)				383.4	
ANGPT2 peak2 (dimer)	58.5 (monomer) 117.0 (dimer)				140.9	
ANGPT2$^{147-496}$	43.5 (monomer) 87.0 (dimer)	106.8	70.6		90.6	
ANGPT2-FLD-Fc	52.5 (monomer) 105.0 (dimer)	109.7				SFig. 7D
ANGPT2-N$^{19-248}$-Fc	53.8 (monomer) 107.6 (dimer)	nd				SFig. 9C
ANGPT2$^{19-202}$-SUMO	36.3 (monomer) 72.6 (dimer)					SFig. 9E
ANGPT2$^{19-76}$-SUMO	21.8 (monomer) 43.6 (dimer)	21.5	34.8			
ANGPT2$^{19-76}$(SCD)-SUMO β1-ECD complex	126.2			124.4		
ANGPT2$^{19-76}$(SCD)-SUMO β1-Head complex	96.9			89.5		
TIE2-LBD	47.3	52.7	51.8		48.8	
ANGPT2$^{147-496}$ + α5Calf1-2 Complex Peak1	130.2	128.1	114.6	125.5		
ANGPT2$^{147-496}$ + α5Calf1-2 + TIE2-LBD Complex Peak1	177.5	206.2		165.6		
ANGPT1-FLD-Fc	56.0 (monomer) 112.0 (dimer)					SFig. 7D
ANGPT1-N$^{16-262}$-Fc	55.8 (monomer) 111.6 (dimer)					SFig. 9C
RQN-α5Calf1	20.5					
RQD-α5Calf1	20.5					
RRQQ-α5Calf1	20.5					

Supplemental Table 3. Diffraction data collection and refinement statistics for crystal structures.

	α5Calf1-2 complex with ANGPT2-FLD peptide	α5Calf1-2
PDB ID	9HMI	9HMH
Wavelength	0.976	1.000
Space group	P 21 21 21	P 21 21 21
Unit cell	a=49.98, b=69.23, c=137.83, alpha=90.00, beta=90.00, gamma=90.00	a=50.02, b=71.44, c=121.27, alpha=90.00, beta=90.00, gamma=90.00
Resolution range	48.84 - 2.81	46.24 - 1.92
Total reflections	12200	33875
Unique reflections	12200	33875
Wilson B-factor	93.66	38.62
Reflections in refinement	12200	33797
Reflections in free set	610	1995
Rwork	0.20	0.2250
Rfree	0.26	0.2860
FSC average	0.6940	0.9306
RMSD bonds	0.0164	0.0110
RMSD angles	2.7540	2.0190
Ramachandran favoured (%)	87.0	95.9
Ramachandran allowed (%)	9.0	3.8
Ramachandran outliers (%)	3.0	0.3
Rotamer outliers	13.8	6.1
Clash score	17.5	8.4
Overall number of atoms (non-H)	2923	
in macromolecules	2715	
in ligands	70	
in solvent	138	
Average B-factor	90.2	
for macromolecules	112.7	
for ligands	140.7	
for solvent	107.5	

Supplemental Table 4. **Modelling of amino acid substitutions in the Calf1 domain of α5-Integrin** (#). Amino acid residues were selected based on the co-crystal structure of ANGPT2 peptide and α5Calf1-2 and molecular dynamics simulation of α5Calf1-2 - ANGPT2-FLD interaction. The effect of amino acid substitution to binding energy (mutation) and statistical error (DDG error) is shown.

#	residue	mutation	DDG error	#	residue	mutation	DDG error	#	residue	mutation	DDG error
766	R	5.212	0.263	762	G	1.403	1.435	724	V	3.845	0.791
766	K	3.567	0.363	762	S	1.177	1.277	724	I	2.988	0.659
766	V	3.120	0.829	762	N	0.606	0.786	724	P	2.255	0.182
766	I	1.464	0.499	762	A	0.084	1.386	724	G	2.160	0.120
766	T	1.390	1.087	762	Q	0.000	0.000	724	A	2.151	0.166
766	C	0.679	0.693	762	F	-0.267	1.070	724	E	2.036	0.191
766	L	0.550	1.910	762	D	-0.486	1.183	724	D	1.975	0.128
766	E	0.303	0.196	762	W	-0.667	0.874	724	T	1.877	0.351
766	M	0.253	1.066	762	I	-0.704	0.875	724	M	1.791	0.541
766	W	0.043	1.146	762	P	-0.713	0.561	724	C	1.786	0.150
766	Q	0.000	0.000	762	C	-0.820	0.609	724	Y	1.748	0.760
766	N	-0.157	0.280	762	H	-0.958	0.607	724	F	1.728	0.994
766	P	-0.161	0.837	762	E	-1.599	0.997	724	L	1.726	0.789
766	D	-0.247	0.712	762	K	-1.765	0.785	724	Q	1.357	0.194
766	A	-0.548	0.230	762	T	-1.831	0.382	724	S	1.251	0.134
766	G	-0.692	0.439	762	Y	-2.468	0.968	724	W	1.201	0.337
766	S	-1.454	0.672	762	V	-2.474	0.411	724	H	1.155	0.253
766	F	-3.603	1.596	762	L	-2.689	1.020	724	K	1.148	0.326
766	H	-4.233	0.706	762	R	-3.390	0.284	724	R	0.145	0.324
766	Y	-7.820	0.344	762	M	-3.940	0.178	724	N	0.000	0.000
764	I	5.230	0.437	755	C	5.251	1.699	694	P	8.002	0.398
764	R	5.021	0.172	755	E	5.146	1.532	694	K	5.508	1.018
764	F	3.851	0.224	755	K	4.334	1.873	694	I	4.898	0.431
764	Y	3.686	0.234	755	P	4.150	1.613	694	A	4.859	0.426
764	C	3.592	0.629	755	T	3.528	1.564	694	V	4.428	0.326
764	V	3.093	1.389	755	Q	2.660	1.849	694	L	4.385	0.360
764	M	2.797	0.369	755	H	2.610	1.700	694	N	4.328	0.566
764	H	2.539	0.832	755	V	2.392	1.736	694	S	4.321	0.160
764	N	2.301	0.149	755	I	1.075	1.710	694	M	4.246	0.340
764	E	1.902	0.645	755	S	0.035	0.214	694	H	4.150	0.502
764	K	1.881	0.176	755	R	0.000	0.000	694	T	4.126	0.126
764	G	1.797	0.216	755	G	-0.453	0.162	694	Q	3.794	0.379
764	L	1.554	0.397	755	D	-0.637	0.244	694	G	3.774	0.338
764	A	1.510	0.267	755	A	-0.641	0.280	694	F	3.629	0.226
764	T	1.444	0.216	755	N	-0.733	0.543	694	C	3.549	0.497
764	Q	1.215	0.260	755	M	-1.152	1.396	694	Y	3.363	0.229
764	W	0.827	1.356	755	L	-1.774	1.976	694	W	2.373	0.274
764	S	0.359	0.241	755	F	-6.791	1.689	694	D	2.325	0.444
764	D	0.000	0.000	755	W	-11.428	0.367	694	E	2.063	1.045
764	P	-0.471	1.332					694	R	0.000	0.000

Supplemental Table 5. Summary of SAXS experiments.

Protein / protein complex	SAXS / SEC-SAXS	**R_g (Å)**	**D_{max} (Å)**	**V_p (Å^3)**	**SAXS. $M_{wt.}$ (kDa)**	**Seq. $M_{wt.}$ (theoretical kDa)**
ANGPT2$^{147–496}$	SAXS	47.5	138.8	118436	70.6	43 (monomer), 86 (dimer)
α5Calf1-2	SAXS	41.1	129.3	71216	50.1	43.2
TIE2-LBD	SAXS	27.7	73.75	114764	51.8	47.13
ANGPT2$^{147–496}$ - α5Calf1-2 Complex	SAXS	48.5	158.2	194796	114.6	130.2
ANGPT2$^{147–496}$ - α5Calf1-2 Complex	SEC-SAXS	53.7	178.5	246238	125.5	130.2
ANGPT2$^{147–496}$ - α5Calf1-2 - TIE2-LBD Complex	SEC-SAXS	61.1	195	374222	165.6	177.5
ANGPT2$^{19-76}$(SCD)-SUMO	SAXS	29.2	67.5	59102	34.8	21.8 (monomer) 43.6 (dimer)
β1-ECD	SAXS	48.5	213.4	146701	86.3	82.6
ANGPT2$^{19-76}$(SCD)-SUMO β1-ECD complex	SEC-SAXS	52.2	186.7	222798	124.4	126.2
ANGPT2$^{19-76}$(SCD)-SUMO β1-Head complex	SEC-SAXS	33.8	102.2	139769	89.5	96.9

Supplemental Table 6. Summary of surface plasmon resonance experiments.

Figure item	Analyte	Chip type	Ligand	Control	Ligand well (RU)	Control well (RU)
5 C	ANGPT2-FLD-Fc	C1	α5β1-ECD	VEGFR2	1050	715
5 D	α5β1-ECD	CM3	ANGPT2-N-Fc	BSA	2445.7	1577.3
5 E	ANGPT2-FLD-Fc	C1	α5Calf1-2+BSA	BSA	290.4	457.1
5 F	α5Calf1	C1	ANGPT2-FLD-Fc	IgG-Fc	5914	2633
5 H	β1-ECD	CM3	ANGPT2-N-Fc	IgG-Fc	1988.4	2202.5
5 I	β1 Headpiece/Leg piece	CM5	ANGPT2-N-Fc	BSA	4619.7	4669.1
7 F	$ANGPT2^{147–496}$	C1	α5Calf1-2	BSA	261	221
S7 A	rhAng2	C1	α5β1-ECD	VEGFR2	1050	715
S7 B	FNIII/ANGPT2-FLD-Fc	C1	α5β1-ECD	VEGFR2	1050	715
S7 D	ANGPT2-FLD(-Fc)	C1	α5Calf1-2+BSA	BSA	290.4	457.1
S9 B	α5Calf1-2	CM3	ANGPT1-FLD-Fc	BSA	1148	566
S9 D	β1-ECD	CM3	ANGPT1-N-Fc	IgG-Fc	2713	1346
S10 A	α5Calf1-2+/-$ANGPT2^{352-366}$	CM3	ANGPT2-FLD-Fc	IgG-Fc	5914	2633
