# Supplementary material for: Inactive β1-integrin acts as a junctional scaffold for angiopoietin/TIE2/FOXO1 signaling
Source: J Clin Invest. 2026 Jun 15;136(12):e190552. doi: 10.1172/JCI190552 (PMC13262729; doi:10.1172/JCI190552)

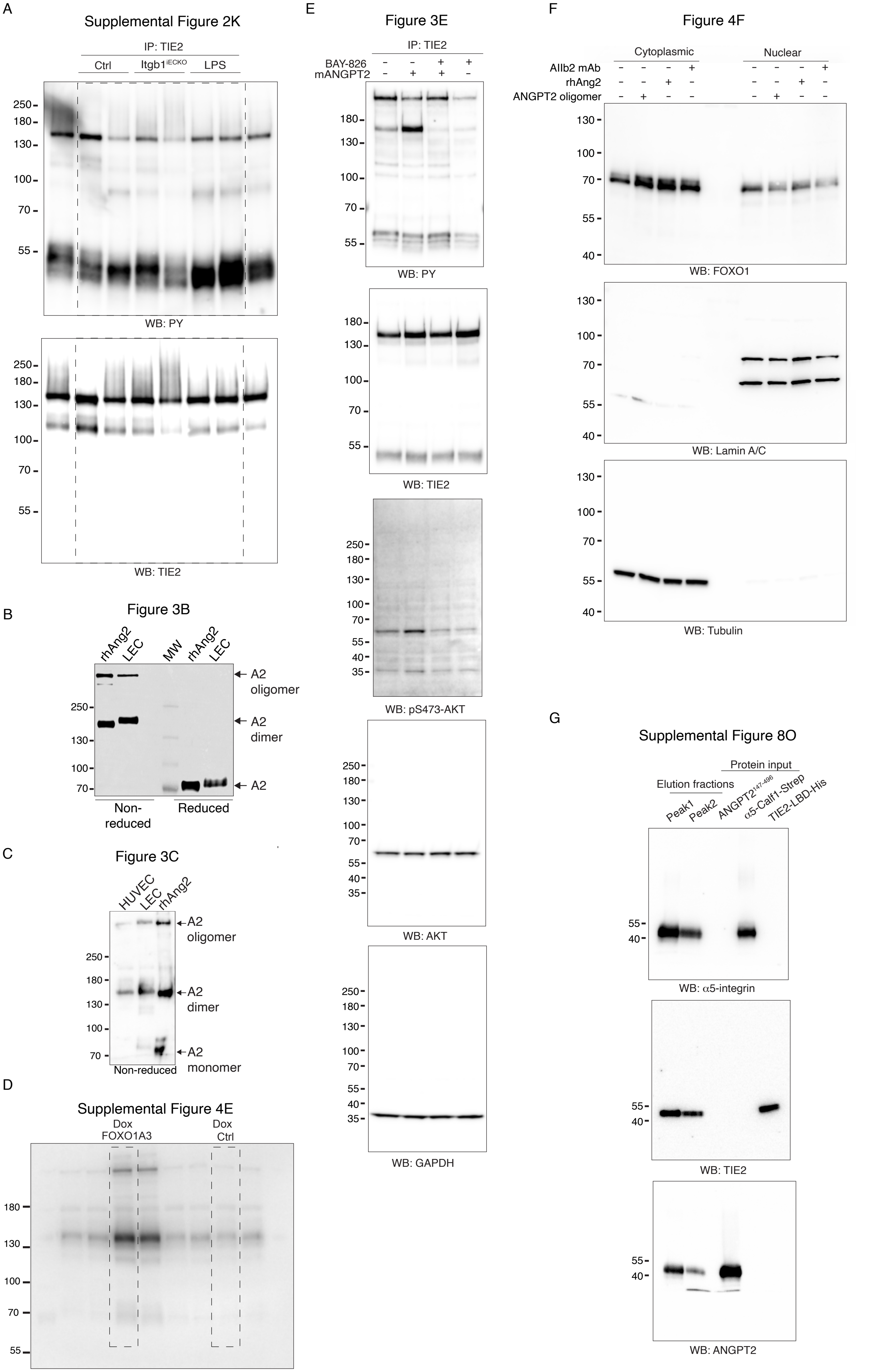

Supplemental Figure 15. Complete unedited Western blots for (**A**) Supplemental Figure 2K (**B**) Figure 3B, (**C**) Figure 3C, (**D**) Supplemental Figure 4E, (**E**) Figure 3E, (**F**) Figure 4F, (**G**) Supplemental Figure 8O, (**H**) Supplemental Figure 13J.

H

Supplemental Figure 13J

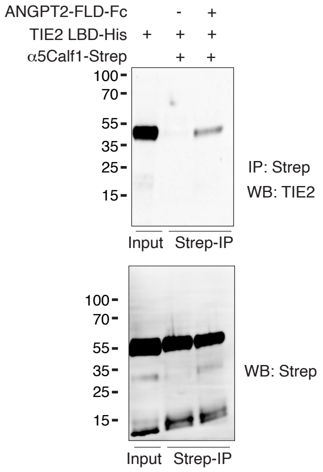

Supplement: Unedited blot and gel images [file jci-136-190552-s310.pdf]
